# Therapeutic methods and effect on keloid and hypertrophic scars: a systematic review

**DOI:** 10.3389/fmed.2026.1702697

**Published:** 2026-03-11

**Authors:** Yuhang Shen, Lirong Yang, Dayong Feng, Chunhui Wang, Zhiyong Bai, Xi Wang, Jingwen Wang, Yuening Feng, Ayue An

**Affiliations:** 1 Department of Anorectal, Wangjing Hospital, China Academy of Chinese Medical Sciences, Beijing, China; 2 Department of Surgery, Central University of Finance and Economics (Shahe Campus) School Hospital, Beijing, China

**Keywords:** bleomycin, cryotherapy, fluorouracil, hypertrophic scar, imiquimod, interferons, keloid, lasers

## Abstract

**Background:**

Keloids and hypertrophic scars are fibroproliferative disorders with high recurrence rates, lacking a definitive treatment standard. This review systematically evaluates current therapies and their effectiveness in treating keloid and hypertrophic scars.

**Method:**

The inclusion criteria were based on the population, intervention, comparator, outcomes, and study design (PICOS) framework. Electronic searches through April 2025 across databases such as PubMed, EMBASE, Cochrane Library, and Web of Science used keywords such as ‘keloid’, ‘occlusive dressings’, and ‘imiquimod’, among others. Meanwhile, we used the keywords *‘Antigens, CD’* and *‘MicroRNAs’* to search for molecular mechanisms associated with keloid and hypertrophic scars. The Risk of Bias 2 (RoB2) and Methodological Index for Non-Randomized Studies (MINORS) checklists were used to assess the quality of the included studies and potential bias.

**Results:**

This study synthesizes findings from 162 studies, exploring a range of treatments including monotherapies and combination therapies, such as local corticosteroid injections, optical therapy, radiation therapy, 5-fluorouracil (5-FU) therapy, bleomycin therapy, verapamil therapy, excision surgery, cryotherapy, and topical treatments, as well as various multi-drug regimens. It also examines innovative therapies such as stem cells and RNA microneedles. Technological developments continue to expand the range of available interventions. Treatment strategies increasingly emphasized combination therapies that integrate intralesional corticosteroids, surgical excision, laser modalities, and radiotherapy, demonstrating superior outcomes compared with single-modality approaches, particularly in reducing recurrence, prolonging therapeutic benefit, and improving patient prognosis.

**Conclusion:**

Sole treatments and inadequate therapy are major risk factors for recurrence. Anti-fibroblast growth strategies are crucial, aside from physical interventions. Despite the lack of an established gold standard, corticosteroid and excision therapies remain critical benchmarks for evaluating new treatments.

## Introduction

Hypertrophic scars and keloids represent pathological skin conditions resulting from abnormal wound healing mechanisms (1). Keloids develop at sites of trauma or infection and are more common in areas of the skin with increased tensile tension and frequent activity (such as the torso, upper arm/shoulder (deltoid area), and knees) (2, 3). The clinical features of keloid are raised but smooth surface scars, hard on palpation, and hypopigmentation and hyperpigmentation. It may be accompanied by symptoms such as itching and pain (3). Because of its potential psychological and physical adverse effects, patients’ quality of life may be impaired (4).

The combination of cells and chemicals promotes and completes the normal tissue repair process. During normal scar healing, collagen production and degradation are usually balanced. In keloids and hypertrophic scars, this balance is disrupted, with increased collagen synthesis and decreased collagen degradation (5). Collagen accumulates in the lesion, leading to excessive scar tissue. Hypertrophic scars and keloid formation are associated with patient age, family history, pregnancy, injury site, secondary trauma, post-injury infection, burn, and tension suture (6, 7). Genetic predisposition and cutaneous injury are major contributors to keloid pathogenesis (6). The incidence is 4.5 to 16% in blacks and Hispanics (6), with lighter skin tone being a protective factor, affecting whites ranging from 5 to 37% (8). Recurrence of keloid and hypertrophic scars after treatment is a major clinical challenge (9).

Clinically and pathologically, there are some differences between keloids and hypertrophic scars (7). Keloids are clinically characterized by raised lesions that extend beyond the original wound margins and do not spontaneously regress (10). The onset time is several months after the injury, and the growth appears to be gradual and undefined. Although keloids vary in size and shape, most are associated with itching and burning. Hypertrophic scars are generally growths less than 1 cm wide that grow in the original skin lesion area. It usually occurs about 4 weeks after the original injury and grows strongly over several months, resolving itself within a year (7).

No universally accepted treatment standard exists, and the effectiveness of available interventions remains controversial (9). Combined interventions are the current routine option for both types of scar treatment (1, 11). Although evidence of superiority is inconclusive, combined modalities frequently exhibit synergistic or complementary effects or are applied sequentially at different treatment stages. Principal therapeutic approaches include pharmacotherapy, pressure therapy, surgical excision, radiotherapy, and light-based therapies (12). Corticosteroids are considered first-line drug therapy for the treatment of hypertrophic scars and keloids, and are the most commonly used option for intralesional administration (11). Other intrafocal drug treatment options include bleomycin (6) and 5-fluorouracil (5-FU) (10). Topical drug forms include gels, creams, sprays, or flexible gel tablets (6). Cryosurgery and scar removal are currently available surgical procedures that may be useful. Cryotherapy techniques lead to scar tissue necrosis by reducing blood flow (6, 13). Scar excision includes the removal of the core of the scar or the overall removal of the scar (11). Light source therapy includes intense pulsed light (IPL) therapy and laser therapy. IPL is designed to promote vascular ischemia and interfere with collagen production (14), whereas laser therapy reduces scar volume by targeted tissue ablation (6, 13).

Despite numerous therapeutic options, diagnosis remains challenging, and recurrence rates remain high. Clinical diagnostic accuracy for keloids has been reported at approximately 81% (15). The recurrence rate in patients treated with surgical resection alone is 70 to 100% (16). With an in-depth understanding of the pathological mechanisms of keloid, the range of related treatment methods is increasingly enriched. For example, the important role of vitamin D in the synthesis and degradation of collagen in the treatment of skin fibrosis diseases such as scleroderma (17); Intralesional corticosteroid injection, cryotherapy, pressure therapy, radiotherapy, laser therapy, onion extract, topical imiquimod cream, bleomycin, interferon and photodynamic therapy were used to inhibit collagen synthesis in keloid tissue (18); The sHLA-E profile may inform trials evaluating systemic agents, such as monoclonal antibodies, in combination with local therapies (19).

Given the multiplicity of interventions and the absence of a definitive gold standard, a systematic review that synthesizes indications, methodologies, and clinical efficacy of available therapies is warranted.

## Methods

### Research design

The present systematic review was conducted according to the PRISMA (Preferred Reporting Items for Systematic Reviews and Meta-analyses statement) (20).

### Search strategy and data sources

We searched PubMed, EMBASE, Cochrane and Web of Science with a pre-designed search strategy in April 2025 to retrieve all relevant clinical trials, using the following search strategy: [*‘keloid’ and (‘occlusive dressings’ or ‘imiquimod’ or ‘steroids’ or ‘mitomycin’ or ‘fluorouracil’, ‘interferons’ or ‘bleomycin’ or* ‘surgical procedures, operative’ *or ‘cryotherapy’, ‘radiotherapy’ or ‘lasers, dye’ or ‘platelet-rich plasma’)*], as well as other relevant key words (Supplementary Table 1). Additional searches using the terms *‘Antigens, CD’* and *‘MicroRNAs’* were conducted to identify studies addressing the molecular mechanisms associated with keloid and hypertrophic scars. The detailed search strategy for all databases is reported in Supplementary Table 1. The reference lists of relevant articles and reviews were screened for additional eligible studies, and manual searches were performed as necessary. Each study was assessed by two independent reviewers, and disagreements were resolved by discussion with a third reviewer.

### Inclusion and exclusion criteria

Our selection criteria were generated based on the PICOS principle as follows.

Inclusion criteria:

P: Patients with a clinical diagnosis of keloid or hypertrophic scar;

I: Intervention groups or control groups received any anti-keloid treatment;

C: No restriction on the intervention of control groups;

O: Studies detailed the treatment strategy or molecular mechanisms for keloid, and provided outcome indicators such as clinical remission rate, scar area improvement, and scar score scale to evaluate the treatment effect;

S: Single-arm or controlled design for treatment-related studies and no restrictions on molecular mechanisms-related studies;

Language: studies published in English.

Exclusion criteria:

- Ineligible study design, such as case series, observational studies, commentary, conference abstracts;
- Essential data were absent from studies, although authors were emailed to obtain it;
- Older duplicate reports published by the same team based on the same group of participants;
- Studies included ineligible participants, such as participants with hypertrophic scars or dermatofibrosarcoma protuberans.

### Data extraction

Data were extracted into a pre-designed Excel spreadsheet. Paired independent reviewers extracted study data and resolved disagreements by discussion or consultation with a third reviewer. For studies with missing data, we contacted the authors to request the necessary information as the remaining data were publicly available as reported in this study. The characteristics of the included studies are summarized as follows: name of the first author, year of publication, study country, study design, clinical diagnosis, sample size, mean age, intervention of experimental group, intervention of controlled group, radiotherapy parameter, follow-up period, treatment evaluation tool, treatment evaluation outcomes (experimental group and controlled group), and main conclusion.

### Quality assessment

Based on the different study designs of the included studies, we selected two different scales for quality assessment. The RoB2 (21) was selected to assess the risk of bias and quality of evidence of the 48 included RCTs. The MINORS (22) was used to evaluate the potential bias and quality of the remaining 101 non-randomized trials. Quality assessment was conducted by two investigators.

**Figure 1:**
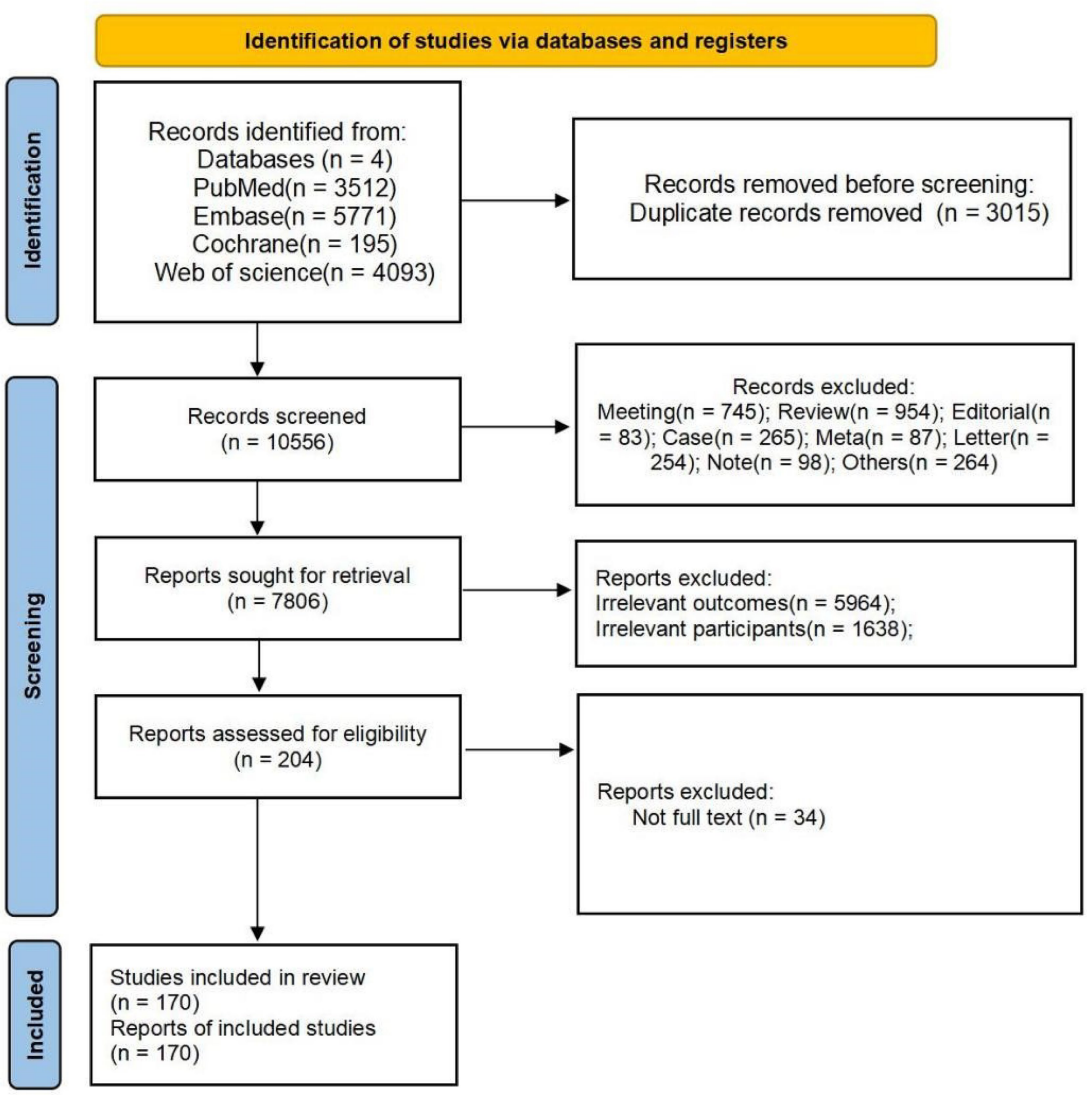
Flowchart of the study selection.

RoB2 assessed bias across domains, including the randomization process, deviations from intended interventions, missing outcome data, measurement of the outcome, and selection of the reported result. MINORS consists of a total of 12 programs, requiring a study with a clear purpose, inclusion of consecutive patients, prospective data collection, selection of endpoints appropriate for the purpose of the study, unbiased evaluation of the endpoints, matching the primary endpoint for the follow-up period, follow-up loss less than 5%, and prospective calculation of the sample size. Articles 9 through 12 provide additional criteria for evaluating control studies, including selection of the gold standard as intervention for the control group, baseline equivalence between groups, and statistical analysis consistent with the study design. Each MINORS item is scored 0–2 (0 = not reported, 1 = reported inadequately, 2 = reported adequately), yielding a maximum score of 24 for comparative studies (Figure 1).

## Results

### Results of study selection

In total, 13,571 articles were identified in electronic and manual searches. However, 3,015 articles were excluded for duplication. A total of 2,750 studies were excluded because their study designs did not fulfill the established inclusion criteria. This exclusion comprised 745 meeting abstracts, 954 reviews, 83 editorials, 265 case reports, 87 meta-analyses, 254 letters, 98 notes, and 264 studies of other designs. After excluding 5,964 studies for irrelevant results and 1,638 studies with inapplicable participants, a total of 204 studies were screened for full-text evaluation. Ultimately, 162 studies concentrated on treatment methodologies, while eight studies examined mechanisms of action (17–19, 23–27); both categories were included in the systematic review.

### Study characteristics

The basic characteristics of the 162 included full-text studies are presented in Table 1. The main conclusions of these studies were summarized in Table 2. One hundred sixty-two studies were conducted in 30 countries, most of them were performed in China (*n* = 23), Egypt (*n* = 19), the USA (*n* = 16), India (*n* = 14), Iran (*n* = 14), and Korea (*n* = 13). Some studies have been published in Japan (*n* = 6), Thailand (*n* = 6), Germany (*n* = 5), Netherlands (*n* = 5), France (*n* = 4), Spain (*n* = 4), Turkey (*n* = 4), Pakistan (*n* = 3), Brazil (*n* = 3), England (*n* = 3), Israel (*n* = 3), Singapore (*n* = 3), Iraq (*n* = 2), and Italy (*n* = 2). The remaining 10 studies were conducted Canada, Greece, Chile, Finland, Ghana, Malaysia, Indonesia, Mexico and Denmark. Study designs comprised 75 cohort studies, 52 randomized controlled trials (RCTs), 25 retrospective studies, and 10 prospective studies. Of the 162 studies, 112 targeted the population with keloids, 17 included patients with hypertrophic scars, 28 included people with keloids and hypertrophic scars, and 5 studied included patients with keloids or hypertrophic scars. A total of 9,510 patients with keloid and/or hypertrophic scars were included in our analysis, with sample sizes ranging from 6 to 612 per study. Most patients were young adults with a mean age of 30.69 years, and the mean age of patients included in every single study ranged from 12 to 60.18 years. The follow-up period of the included studies varied widely, ranging from 1 month to 10 years. Due to the diversity of treatment modalities included in the studies, study outcome measures also varied greatly, and most studies only assessed the efficacy of treatment regimens and/or the impact on recurrence rates (*n* = 88). Twenty-four of the included studies assessed scars at each stage using the Vancouver Scar Scale (VSS) (28). Furthermore, a limited number of studies employed single scar assessment scales, including Manchester Scar Scale (MSS), Modified Vancouver Scar Scale (MVSS), Patient and Observer Scar Assessment Scale (POSAS), Patient Scar Assessment Scale (PSAS), Stony Brook Scar Evaluation Scale (SBSES), and Japan Scar Workshop Scar Scale (JSS), or utilized a combination of multiple outcome indicators to evaluate the efficacy of treatment.

**Table 1:** Characteristics of the included studies.

Study	Country	Study design	Patients	Sample size	Age	Intervention of the experimental group	Intervention of the controlled group	Follow up
Daoud 2019 (137)	USA	RCT	Large (>100 cm^2^) hypertrophic scars	23 s	\	Combined Intense Pulsed Light (IPL) With Fractional CO_2_-Laser	Fractional CO_2_-Laser	6 months
Manuskiatti 2021 (58)	USA	RCT	Abdominal hypertrophic scars	19	34	Laser+steroid	Laser + petrolatum	6 months
Son 2014 (57)	Korea	Cohort study	Keloids or hypertrophic scars	12	34.5	578 nm Copper Bromide Laser Combined with Intralesional Corticosteroid Injection	\	1 month
Lee 2015 (76)	Korea	Cohort study	Keloids or hypertrophic scars	30	23.9	6-MeV electron beam	\	27.4 months
Ahuja 2013 (92)	India	RCT	Keloids or hypertrophic scars	40	\	Intralesional verapamil hydrochloride (concentration 2.5 mg/mL)	Intralesional triamcinolone acetonide (concentration 40 mg/mL)	\
Martin-Garcia 2005 (108)	USA	Cohort study	Earlobe Keloid	6	20.17	Imiquimod 5% Cream	\	2 years
Al-Mohamady 2016 (138)	Egypt	RCT	Keloids or hypertrophic scars	20	22.6	595-nm PDL laser	Nd: YAG laser	1 month
Tawfic 2020 (71)	Egypt	RCT	Hypertrophic Scars and Keloids	30	25.97	Combined: Fractional and Nd YAG	Nd: YAG alone/Fractional laser alone	1 month
Kant 2018 (93)	Netherlands	Retrospective study	Hypertrophic and keloid scars	58	28.1	Triamcinolone and verapamil	\	\
Li 2020 (139)	China	Prospective study	Hypertrophic scars and keloids	21	28.43	Intralesional 1,470 nm bare-fiber diode laser	\	\
Wen 2021 (140)	China	Retrospective study	Keloids	100	28	Hypofractionated radiotherapy	\	4.92 years
Hatamipour 2009 (141)	Iran	RCT	Keloids	50	\	combined topical silicone and 5-Fluorouracil	Topical silicone	1 year
Saha 2012 (142)	India	RCT	Keloids	44	33.75	5-FU (50 mg/mL)	Triamcinolone acetonide (40 mg/mL)	1 year
Daurade 2020 (143)	France	Retrospective study	Keloids	38	\	Combining surgical excision and high-dose-rate brachytherapy	\	6 months
Chen 2020 (111)	China	Cohort study	Keloids	40	27.1	Continuous tension reduction	\	2 years
Wang 2020 (67)	China	Cohort study	Keloids	41	27.4	combined Ultrapulse Fractional Carbon Dioxide Laser and Topical Triamcinolone	\	2 years
Levenberg 2020 (144)	Israel	Cohort study	Keloids	21	\	Intralesional 5FU and corticosteroids	\	3 months
Khattab 2019 (145)	Egypt	RCT	Keloids	40	31.55	Intralesional verapamil alone 2.5 mg/mL.	Combination of PDL and intralesional verapamil alone 2.5 mg/mL	6 months
Bonnardeaux 2019 (119)	Canada	Cohort study	Keloids	21	\	Surgical excision combined with fully ablative carbon dioxide laser therapy and triamcinolone injections	\	18 months
Seo 2011 (117)	Korea	Cohort study	Keloids and hypertrophic scars	9	44.11	Topical and intralesional mitomycin C (1 mg/mL)	\	6 months
Arnault 2009 (146)	France	Cohort study	Keloids	55	\	Iridium 192* brachytherapy	\	\
Yii 1996 (147)	England	Cohort study	Keloids and hypertrophic scars	41	\	Cynthaskin and topical steroid	\	\
Bijlard 2017 (148)	Netherlands	Cohort study	Keloids	238	\	High-Dose-Rate Brachytherapy	\	\
Kaushal 2020 (149)	India	RCT	Keloids	60	33.57	Combined intralesional radiofrequency and intralesional triamcinolone acetonide	Intralesional triamcinolone acetonide alone	\
Sabry 2020 (150)	Egypt	RCT	Hypertrophic scars and keloids	20	12.5	combined laser and intralesional injection of botulinum toxin A	Intralesional injection of botulinum toxin A	\
Bischof 2007 (128)	Germany	Cohort study	Keloids	47	36.6	6-MeV electron beam radiotherapy	\	5.8 years
Francisco 2013 (151)	Spain	Cohort study	Keloids	10	25.7	Combination of bleomycin and triamcinolone acetonide	\	\
Cho 2010 (5)	Korea	Cohort study	Keloids and hypertrophic scars	12	23.8	1,064-nm Q-switched Nd: YAG laser with low fluence	\	3 months
Chopinaud 2014 (98)	France	Retrospective study	Keloids	10	\	Intralesional Cryosurgery	\	\
Clavere 1997 (152)	France	Cohort study	Keloids	39	\	Iridium 192 brachytherapy	\	\
Abedini 2018 (91)	Iran	RCT	Keloids and hypertrophic scars	50	\	Intralesional verapamil (2.5 mg/mL)	Intralesional corticosteroids (40 mg/mL)	3 months
Copcu 2004 (94)	Turkey	Cohort study	Keloids	21	20.61	Combination of Surgery and Intralesional Verapamil Injection	\	2 years
Careta 2013 (120)	Brazil	Cohort study	Keloids	12	20.08	Shaving, Cryosurgery, and Intralesional Steroid Injection	\	\
Asilian 2006 (3)	Iran	RCT	Keloids	69	24.73	Combination of Triamcinolone, 5-Fluorouracil, and Pulsed-Dye Laser	Intralesional triamcinolone acetonide (10 mg/mL)/TAC (40 mg/mL) + 5-FU (50 mg/mL)	3 months
Behera 2016 (63)	India	RCT	Keloids	60	\	Carbon Dioxide Laser	Cryotherapy	1 year
Carvalhaes 2015 (121)	Brazil	Cohort study	Earlobe keloids	46	\	Triamcinolone injections, surgical resection, and local pressure	\	2 years
Espana 2001 (153)	Spain	Cohort study	Keloids and hypertrophic scars	13	23.85	Bleomycin	\	\
Weshahy 2012 (154)	Egypt	Cohort study	Keloids and hypertrophic scars	22	\	Combined intralesional cryosurgery and intralesional steroid injection	\	4 months
Manjunath 2021 (81)	India	Cohort study	Keloids	50	\	Surgical Excision and Adjuvant High-dose Rate Brachytherapy	\	6 months
Bijlard 2018 (101)	Netherlands	RCT	Keloids	26	33.78	Intralesional cryotherapy	Excision with corticosteroid injections or brachytherapy	\
Alhamzawi 2021 (68)	Iraq	Cohort study	Keloids	24	24.25	Fractional Carbon Dioxide Laser with Intralesional 5-Fluorouracil (50 mg/mL)	\	\
Erol 2008 (14)	Turkey	Cohort study	Keloids and hypertrophic scars	109	\	Intense Pulsed Light	\	\
Monteiro 2022 (155)	India	RCT	Keloids	30	\	Intralesional 5 Fluorouracil (50 mg/mL)	Combination of 5 Fluorouracil (50 mg/mL) with Triamcinolone Acetonide (40 mg/mL)	6 months
Escarmant 1993 (82)	USA	Cohort study	Keloids	544	24	Iridium 192 interstitial irradiation after surgical excision	\	6 years
Annabathula 2017 (156)	India	Cohort study	Keloids	15	\	Fractional Carbon Dioxide, Long Pulse Nd: YAG and Pulsed Dye Laser	\	\
Berman 2020 (157)	USA	Retrospective study	Keloids	61	38.9	Superficial Radiation Therapy	\	2 years
Li 2022 (158)	China	RCT	Keloids	60	29.49	Excision followed by 5-FU and betamethasone intralesional injections	5-FU and betamethasone intralesional injections/excision followed by radiotherapy	8–12 months
Barragan 2022 (159)	Spain	Cohort study	Keloids	51	46	Interstitial high-dose-rate brachytherapy	\	4 years
Jiang 2015 (73)	Germany	Prospective study	Keloids	24	\	Interstitial high-dose-rate brachytherapy	\	\
Jiang 2017 (74)	Germany	Cohort study	Keloids	29	\	Interstitial high-dose-rate brachytherapy	\	4.14 years
Guix 2001 (160)	Spain	Cohort study	Keloids	169	\	Interstitial high-dose-rate brachytherapy	\	7 years
Hafkamp 2017 (161)	Netherlands	Prospective study	Therapy-resistant keloids	24		Interstitial high-dose-rate brachytherapy	\	2.21 years
Meymandi 2014 (69)	Iran	Cohort study	Keloids	86	\	Intense Pulsed Light Method Along With Corticosteroid Injection	\	\
Stephanides 2011 (162)	England	Cohort study	Refractory Keloids	99	\	Intralesional triamcinolone (10 mg or 40 mg/dL) and pulsed dye laser	\	\
Choi 2020 (31)	Korea	Cohort study	Auricular keloid	18	26.5	Triamcinolone acetonide intralesional injection	\	2 years
Darougheh 2007 (163)	Iran	RCT	Keloids and hypertrophic scars	40	24.3	Intralesional triamcinolone acetonide	Combination of TAC and 5-fluorouracil	3 months
Davari 2012 (65)	Iran	RCT	Keloids and hypertrophic scars	7	30.29	Pulsed dye laser	9 weeks after suture removal	\
Muneuchi 2009 (32)	Japan	Cohort study	Keloids	94	42	Intralesional injection of triamcinolone acetonide	\	5 years
Kim 2015 (164)	Korea	Cohort study	Keloids and hypertrophic scars	52	\	intense pulsed light device and intralesional corticosteroid injection	\	\
Kim 2022 (40)	Korea	Retrospective study	Hypertrophic cesarean section scars	35	34.91	pulsed dye laser therapy combined with intralesional triamcinolone injection	\	7.74 months
Moravej 2022 (87)	Iran	RCT	Keloids and hypertrophic scars	60	33.95	Intralesional bleomycin (1.5 mg/mL)	Intralesional triamcinolone (20 mg/mL)	\
Saki 2019 (36)	Iran	RCT	Keloids	15	\	Intralesional Triamcinolone Acetonide	Intralesional Verapamil	6 months
George 2005 (133)	Greece	Cohort study	Keloids	20	30.05	Intralesional 5-fluorouracil	\	2 years
Hye 2015 (70)	Korea	Cohort study	Hypertrophic Thyroidectomy Scar	67	37	Copper bromide laser and intralesional triamcinolone injection (2.5 mg/mL or 5 mg/mL)	\	\
Maemoto 2020 (134)	Japan	Cohort study	Keloids	59	\	Electron beam radiation therapy	\	10 years
Berman 2002 (109)	USA	Cohort study	Keloids	12	\	Imiquimod 5% cream	\	6 months
Chen 2017 (124)	China	RCT	Keloids	39	26.73	Intralesional injection of diprospan (2 mg betamethasone disodium phosphate and 5 mg betamethasone dipropionate in 1 mL)	Intralesional injection of diprospan with a combination of 0.5 mL 5-fluorouracil (25 mg/mL)/diprospan + 5-FU + 1,064-nm Nd: YAG laser	\
Ogawa 2002 (165)	Japan	Retrospective study	Keloids and hypertrophic scars	147	\	Electron beam radiation therapy	\	18 months
Friedman 2020 (166)	Israel	RCT	Surgical scars	11	47.2	Erbium glass, 1,540 nm laser	not treated	1 year
Aljodah 2021 (167)	Iraq	Cohort study	Recurrent Auricular Keloid	41	29	Combination of Surgical Excision and Perioperative Corticosteroid Injection (40 mg/mL)	\	13.04 months
Park 2012 (168)	Korea	Cohort study	Facial Keloids	15	34	Intralesional Steroid Injections (20 mg/mL)	\	18 months
Acosta 2016 (33)	Chile	prospective study	Keloids	21	12	Intralesional Triamcinolone (40 mg/mL)	\	5.58 years
Ogawa 2014 (169)	Japan	Retrospective study	Auricular Keloid	57	\	Surgical excision and radiotherapy	\	18 months
Ollstein 1981 (80)	USA	Cohort study	Keloids	40	\	Combined surgical excision and immediate X-ray therapy	\	2 years
Reinholz 2020 (41)	Germany	Cohort study	Keloids	25	28.68	Intralesional 5-fluorouracil (50 mg/mL) in combination with triamcinolone acetonide (40 mg/mL)	\	1 year
SONG 2018 (112)	China	RCT	Keloids	240	\	hyperbaric oxygen therapy	Surgical excision and radiotherapy	\
Song 2014 (170)	Korea	Cohort study	Intractable keloids	12	32	Single-fraction radiotherapy	\	20 months
Burusapat 2021 (171)	Thailand	RCT	Auricular Keloid	34	25.52	Immediate Triamcinolone Acetonide Injection	Delayed Triamcinolone Acetonide Injection	6 months
Shen 2015 (172)	China	Cohort study	Keloids	568	\	Hypofractionated electron-beam radiation	\	40 months
Ragoowansi 2002 (171)	England	Cohort study	Keloids	80	\	Surgical Excision and immediate single-fraction radiotherapy	\	5 years
Hewedy 2020 (54)	Egypt	RCT	Keloids	40	29.05	Intralesional triamcinolone acetonide (20 mg/mL) and Platelet rich plasma	TA (20 mg/mL) alone	3 months
Hietanen 2018 (39)	Finland	RCT	Keloids	43	42	Intralesional triamcinolone		6 months
Weshay 2015 (173)	Egypt	Cohort study	Keloids	18	32.78	Combination of Radiofrequency and Intralesional Steroids (10 mg/mL)	\	5 years
Agbenorku 2000 (122)	Ghana	Cohort study	Keloids	120	\	A triple therapy comprising the use of steroid injections and cream (triamcinolone acetonide), surgery, and silicone gel strip/sheet pressure application	\	13 months
Ahmad 2017 (174)	Pakistan	Cohort study	Keloids	51	22.82	Iridium-192 high-dose rate surface mould brachytherapy	\	33 months
Saray 2005 (175)	Turkey	Cohort study	Keloids and hypertrophic scars	14	32.57	Dermojet injections of bleomycin (1.5 IU/mL)	\	19 months
Tawaranurak 2022 (42)	Thailand	RCT	Keloids	22	43.7	Treated with fractional CO_2_ laser + topical triamcinolone	Intralesional TA	1 year
Sruthi 2017 (75)	India	Cohort study	Keloids	13	\	Single-fraction radiation	\	32.67 months
Son 2020 (77)	USA	Cohort study	Keloids	15	43.87	A single dose of low-energy superficial X-ray radiation	\	6 months
Shaarawy 2014 (113)	Egypt	RCT	Keloids	24	29.29	Intralesional botulinum toxin type A	Intralesional steroid	7 months
Song 2018 (112)	China	Cohort study	Keloids	108	\	Intralesional triamcinolone acetonide injection	\	\
Ramadan 2021 (90)	Egypt	RCT	Keloids and hypertrophic scars	40	\	Pulsed Nd: YAG laser and intralesional bleomycin	Pulsed Nd: YAG laser only	\
Suwanchinda 2022 (115)	Thailand	RCT	keloids and hypertrophic scars	18		Cold atmospheric-pressure plasma	Untreated	1 month
Luo 2023 (89)	China	Cohort study	Treating refractory keloids and hypertrophic scars	86	\	The combined application of bleomycin and triamcinolone	\	2–5 years
Neinaa 2021 (114)	Egypt	RCT	Keloids	60	25	Intralesional injection of botulinum toxin type-A (5 IU/injection point)	Intralesional injection of platelet rich plasma (0.1 mL/injection point)/intralesional injection of triamcinolone acetonide (20 mg/session)	\
Nor 2016 (110)	Malaysia	RCT	Keloids	21	29	Either daily topical clobetasol propionate 0.05% cream under occlusion with a silicone dressing	Monthly intralesional triamcinolone injection	3 months
Noruri 2003 (176)	USA	RCT	Surgical scars	11	60.18	585-nm pulsed dye laser	Not treated	1 month
Khalid 2018 (45)	Pakistan	RCT	Ear keloids	60	31.82	Intralesional 5-FU/triamcinolone acetonide injections	Radiotherapy	6 months
Khan 2019 (88)	Pakistan	RCT	Keloids	164	32.5	Intralesional bleomycin	Intralesional triamcinolone	6 months
Khedr 2019 (177)	Egypt	RCT	Hypertrophic scars	50	16.64	Nd: YAG laser	combined intense pulsed light and radiofrequency	3 months
Stern 1989 (66)	USA	Prospective study	Earlobe Keloids	18	\	Carbon dioxide laser excision	\	\
Stewart 2006 (96)	USA	Retrospective study	Head and neck keloids	10	21	The combination of surgical excision with the application of topical mitomycin-C	\	8 months
Leeuwen 2014 (100)	Netherlands	Prospective study	Keloids	27	\	Intralesional Cryotherapy	\	1 year
Viani 2009 (178)	Brazil	Retrospective study	Keloids	612	25	Strontium 90 brachytherapy	\	61 months
Manuskiatti 2021 (58)	Thailand	RCT	Hypertrophic scars	21	35.5	Thermomechanical fractional injury-assisted topical corticosteroid (40 mg/mL)	Corticosteroid injection (40 mg/mL)	6 months
Erlendsson 2022 (47)	Denmark	RCT	Hypertrophic scars	20	\	A pneumatic jet injection with 5-fluorouracil and triamcinolone acetonide	5-FU + TAC	1 month
Wang 2020 (179)	China	Cohort study	Keloids	58	33	Combined surgical excision and electron external beam radiation	\	22 months
Davison 2009 (180)	USA	Retrospective study	Keloids and hypertrophic scars	102	\	5-FU + steroid with excision	5FU + steroid without excision/steroid treatment with excision	6 years
Dai 2021 (181)	China	Retrospective study	Keloids	50	41	Combination of ablative fractional carbon dioxide laser and platelet-rich plasma	Ablative fractional carbon dioxide laser	6 months
Ang 2013 (61)	Singapore	Retrospective study	Earlobe keloids	16	20	Carbon dioxide laser ablation	Cold steel debulking surgery	2 years
Nishi 2022 (104)	India	Cohort study	Keloids	170	\	combination of cryotherapy with intralesional corticosteroid	A combination of fractional CO_2_ laser followed by topical corticosteroids	\
Sharma 2021 (51)	India	Cohort study	Small keloids	40	\	Intralesional 5-FU and triamcinolone acetonide	Combination of intralesional bleomycin and triamcinolone acetonide	\
Abdel-Meguid 2014 (99)	Egypt	Cohort study	Keloids	23	26.21	Intralesional cryosurgery	Contact cryosurgery	\
Lv 2021 (182)	China	Prospective study	Hypertrophic scars	68	40.76	Ablative fractional CO_2_ laser surgery	Conventional surgery	\
Meymandi 2016 (62)	Iran	Cohort study	keloids and hypertrophic scars	166	31.7	Intense pulsed light	Cryotherapy	\
Deng 2021 (123)	Japan	RCT	Keloids	31	24	Intralesional triamcinolone (5 ml 1%) and 5-fluorouracil (0.6 ml 2.5%) injections and Strontium-90 brachytherapy	Intralesional triamcinolone (5 mL 1%) and 5-fluorouracil (0.6 mL 2.5%) injections	14 months
Yosipovitch 2009 (103)	Singapore	Cohort study	Keloids	10	25.9	Cryotherapy and steroid injection	Cryotherapy alone/Steroid injection	\
Emad 2010 (84)	Iran	Prospective study	Keloids	28	28.85	Surgical excision and radiotherapy	Cryotherapy and intralesional steroid	\
Hoang 2016 (79)	USA	Retrospective study	Keloids	128	\	Interstitial high-dose rate brachytherapy	Excision alone/external beam radiotherapy	3.5 years
Berman 1997 (116)	USA	Cohort study	Keloids	124	\	Interferon alfa-2b	Excision alone/injection with triamcinolone acetonide	\
Albalat 2021 (37)	Egypt	Cohort study	Keloids or hypertrophic scars	160	32.25	Intralesional triamcinolone (concentration 40 mg/mL)	Intralesional 5-fluorouracil (concentration 250 mg/5 mL)/intralesional verapamil (concentration 2.5 mg/mL)/intralesional platelet-rich plasma (2.4 mL)	\
Payapvipapong 2014 (183)	Thailand	Cohort study	Keloids and hypertrophic scars	26	34.43	Intralesional triamcinolone acetonide (10 mg/mL)	Intralesional bleomycin (1 mg/mL)	3 months
Rasaii 2018 (49)	Iran	RCT	Keloids	20	23.3	Intralesional triamcinolone (40 mg/mL) in combination with botulinium toxin A (20 mg/mL)	Intralesional triamcinolone (40 mg/mL) alone	1 month
Disphanurat 2023 (184)	Thailand	RCT	Keloids and hypertrophic scars	20	34.25	Triamcinolone acetonide-loaded dissolving microneedle patch	Drug-free DMN patch	1 month
Dina 2021 (107)	Egypt	Cohort study	Keloids	30	26.4	combined fractional ablative 2,940 nm Er: YAG laser and topical application of steroid cream	Intralesional corticosteroid injection	\
Wittenberg 1999 (59)	USA	RCT	Hypertrophic scars	20	48.9	585-nm flashlamp-pumped pulsed-dye laser	Silicone gel sheeting	4 months
Srivastava 2018 (48)	India	RCT	Keloids	60	27.93	Intralesional triamcinolone acetonide (40 mg/mL) and 5-fluorouracil (50 mg/mL)	Intralesional triamcinolone acetonide (40 mg/mL)/intralesional 5-fluorouracil (50 mg/mL)	6 weeks
Sunil 2018 (185)	India	RCT	Keloids	60	30.85	Fractional CO_2_ laser	Intralesional triamcinolone (40 mg/mL)/intralesional verapamil (2.5 mg/ mL)	\
Zouboulis 2020 (102)	Germany	RCT	Small keloids	40	23.5	Combined liquid nitrogen contact cryosurgery with intralesional corticosteroids	Liquid nitrogen contact cryosurgery	3 years
Stromps 2013 (186)	Israel	Retrospective study	Refractory keloids	64	34.68	Intralesional cryotherapy combined with postoperative silicone gel sheeting	Intralesional cryotherapy alone	1 year
Alexander 2018 (55)	India	Cohort study	Keloids and hypertrophic scars	50	\	Fractional CO_2_ laser with intralesional steroid	Intralesional steroid alone	\
Sabry 2019 (64)	Egypt	RCT	Keloids and hypertrophic scars	30	19.75	CO_2_ laser and topically applied 5-FU	CO_2_ laser and topically applied verapamil hydrochloride/ablative fractional CO_2_ laser monotherapy	\
Sadeghinia 2012 (35)	Iran	RCT	Keloids	40	\	Intralesional triamcinolone acetonide	5-FU tattooing	11 months
Gamil 2019 (187)	Egypt	Cohort study	Keloids	50	28.11	Combined intralesional triamcinolone acetonide with botulinum toxin type A	Intralesional triamcinolone acetonide/intralesional botulinum toxin type A	1 month
Dogahe 2023 (52)	Iran	Cohort study	Keloids	43	36.09	Intralesional triamcinolone (40 mg/mL) and verapamil (2.5 mg/mL)	Intralesional triamcinolone alone (40 mg/mL)	3 months
Shin 2019 (43)	Korea	Cohort study	Keloids and hypertrophic scars	38	39.52	Combination of non-ablative fractional laser and intralesional triamcinolone injection	Intralesional triamcinolone injection	\
Sharma 2007 (46)	India	Cohort study	Small keloids	21	\	combination of liquid nitrogen and intralesional triamcinolone acetonide (5 mg/mL)	Liquid nitrogen alone	6 months
Gamil 2018 (188)	Egypt	Cohort study	Acne keloidalis nuchae	30	36.87	Er: YAG laser	Long-pulsed Nd: YAG laser	\
Cicco 2013 (78)	Italy	Cohort study	Keloids	96	\	High-dose-rate interstitial brachytherapy	Low-dose-rate interstitial brachytherapy	2.33 years
Chernoff 2007 (189)	USA	Cohort study	Keloids and hypertrophic scars	30	\	Dermatix gel	Silicone gel sheeting	\
Tsai 2019 (56)	Japan	Retrospective study	Hypertrophic Scars	40	34.2	Combination of 1,064-nm Neodymium-doped Yttrium Aluminum Garnet Laser and Steroid Tape	Steroid tape	6 months
Tawfik 2019 (190)	Egypt	Cohort study	Severe hypertrophic scars	24	26	combined 5-fluorouracil and fractional erbium YAG laser	Topical 5-fluorouracil cream	\
Hou 2023 (191)	China	RCT	Keloids	72	28.75	Punch excision combined with Intralesional steroid injection	Intralesional Steroid Injection alone	\
Liu 2023 (44)	China	Retrospective study	Keloids	29	35.38	combined Pulsed Dye Laser and triamcinolone acetonide	Triamcinolone acetonide	\
Lee 2008 (53)	Korea	Cohort study	Keloids	19	24.6	Triamcinolone acetonide intralesional injection + Interferon-*α*	Triamcinolone acetonide intralesional injection	\
Zawahry 2015 (192)	Egypt	Cohort study	Hypertrophic burn scars	11	30.8	Fractional CO_2_ laser	Untreated	3 months
Meseci 2019 (105)	Turkey	Prospective study	Postcesarean scars	61	31.28	Topical corticosteroid ointment	Untreated	6 months
Qiao 2017 (97)	China	Cohort study	Earlobe Keloid	160	\	Surgery combined with lucortriticod and electron irradiation group		1 year
Francesca 2010 (78)	Italy	Cohort study	Facial Scars	30	37	Self-drying silicone gel		\
Zhou 2023 (193)	China	Retrospective study	Hypertrophic Scars	155	29.7	Ablative fractional carbon dioxide laser +1 g triamcinolone external application	Ablative fractional carbon dioxide laser + 40 mg/mL triamcinolone intralesional injection	1 month
Zhang 2023 (194)	China	RCT	Hypertrophic Scars	101	32	Combined CO_2_ fractional laser and narrowband intense pulsed light	Intense pulsed light	3 months
Li 2024 (195)	China	Prospective study	Hypertrophic Scars	118	\	Keloid-cross-flap surgery and radiotherapy	Keloid-cross-flap surgery and compression therapy	3 months
Pazyar 2024 (196)	Iran	RCT	Keloids	22	35.23	Intralesional vitamin D injection	Intralesional triamcinolone injection	3 months
Khan 2025 (85)	Pakistan	Retrospective study	Keloids	17	41.5	Surgical excision and radiotherapy	\	2 years
Jiang 2024 (197)	China	Retrospective study	Keloids	12	24.42	fractional carbon dioxide laser + 5-aminolevulinic acid photodynamic therapy	\	6 months
Hu 2023 (83)	China	Retrospective study	Keloids	15	\	Surgical resection, ultra-reduced tension suture incision, and superficial radiation therapy	\	6 months
Yang 2025 (198)	China	Retrospective study	Keloids	67	\	Surgical resection + injection of triamcinolone and 5-fluorouracil + radiation therapy	\	>1 year
Park 2024 (199)	Korea	Retrospective study	Keloids	111	35.18	Intralesional triamcinolone + ND: YAG laser	Intralesional Triamcinolone	1 year
Harsono 2023 (30)	Indonesia	RCT	Keloids	24	29.38	Intralesional injection of umbilical cord Mesenchymal stem cells	Intralesional injection of triamcinolone acetonide	4 months
Qiu 2025 (200)	China	Retrospective study	Hypertrophic Scars	42	26.59	Manual fractional technology with CO_2_ laser combined with transdermal triamcinolone acetonide and 5-fluorouracil	\	6 months
Aristides 2024 (201)	Mexico	Retrospective study	Keloids	22	24.3	combined continuous wave and repetitive fractionated CO_2_ laser	\	6 months
Lim 2024 (118)	Singapore	RCT	Keloids	32	43.6	Small interfering RNA microneedle patches	Silicone sheets	2 months

**Table 2:** Main conclusion of the included studies.

Study	Intervention of the experimental group	Intervention of the controlled group	Conclusion
Daoud 2019 (137)	Combined Intense Pulsed Light (IPL) With Fractional CO_2_-Laser	Fractional CO_2_-laser	The experimental group had statistically significant improvement in both color and texture
Abdel-Meguid 2014 (99)	Intralesional cryosurgery	Contact cryosurgery	Intralesional cryosurgery is superior to contact cryosurgery in terms of efficacy and safety
Abedini 2018 (91)	Intralesional verapamil (2.5 mg/mL)	Intralesional corticosteroids (40 mg/mL)	Did not support verapamil’s capability in the treatment of keloid or hypertrophic scars
Acosta 2016 (33)	Intralesional Triamcinolone (40 mg/mL)	\	Triamcinolone acetonide is highly effective for the treatment of pediatric keloids. There is no relationship between clinical response and the factors evaluated, such as lesion location, etiology, and age of the keloid.
Agbenorku 2000 (122)	A triple therapy comprising the use of steroid injections and cream (triamcinolone acetonide), surgery and silicone gel strip/sheet pressure application	\	This is a tedious and time intensive procedure for both physician and patient. A quicker and more readily available method should be sought
Ahmad 2017 (174)	Iridium-192 high-dose rate surface mould brachytherapy	\	10 Gy in a single fraction is therefore the most convenient and cost effective dose regimen for the management of keloid scars in developing countries like Pakistan
Ahuja 2013 (92)	Intralesional verapamil hydrochloride (concentration 2.5 mg/mL)	Intralesional triamcinolone acetonide (concentration 40 mg/mL)	Verapamil can flatten the raised scars, with an extremely low cost and fewer adverse effects
Albalat 2021 (37)	Intralesional triamcinolone (concentration 40 mg/mL)	Intralesional 5-fluorouracil (concentration 250 mg/5 mL)/intralesional verapamil (concentration 2.5 mg/mL)/intralesional platelet-rich plasma (2.4 mL)	Intralesional verapamil was reported to be the most effective therapy, and platelet-rich plasma was effective as intralesional triamcinolone acetonide with no serious side effects; 5-fluorouracil was less effective in treating keloids.
Alexander 2018 (55)	Fractional CO_2_ laser with intralesional steroid	Intralesional steroid alone	Combination therapy with FCL and ILS was superior in efficacy when compared to ILS alone, in the treatment of keloids and HTS
Alhamzawi 2021 (68)	Fractional Carbon Dioxide Laser with Intralesional 5-Fluorouracil (50 mg/mL)	\	Combination therapy with an FCO_2_ laser and intralesional 5-FU showed a promising effect in the treatment of resistant keloids, with an acceptable safety profile and low recurrence rate.
Aljodah 2021 (167)	Combination of Surgical Excision and Perioperative Corticosteroid Injection (40 mg/mL)	\	Perioperative corticosteroid injections combined with surgical excision of auricular keloids are still a valid option in recurrent cases.
Al-Mohamady 2016 (138)	595-nm PDL laser	Nd: YAG laser	Pulsed-dye laser and long-pulsed Nd: YAG laser treatments for keloid and hypertrophic scars provide significant improvement with no significant difference between the modalities.
Ang 2013 (61)	The carbon dioxide laser ablation	Cold steel debulking surgery	Both the CO_2_ laser ablation and cold steel surgery were equally useful in reducing the size of the earlobe keloids, but were not effective in preventing the regrowth of the keloids
Annabathula 2017 (156)	Fractional Carbon Dioxide, Long Pulse Nd: YAG and Pulsed Dye Laser	\	Lasers may have a synergistic effect when combined with other modalities of treatment, but cannot be used as monotherapy in the treatment of keloids.
Arnault 2009 (146)	Iridium 192* brachytherapy	\	The technique is efficient in preventing keloid recurrence and in treating the functional signs, but at the expense of an unaesthetic result
Asilian 2006 (3)	Combination of Triamcinolone, 5-Fluorouracil, and Pulsed-Dye Laser	Intralesional triamcinolone acetonide (10 mg/mL)/TAC (40 mg/mL) + 5-FU (50 mg/mL)	The TAC + 5-FU + PDL combination appears to be the best approach for treating keloid and hypertrophic scars.
Barragan 2022 (159)	Interstitial high-dose-rate brachytherapy	\	The treatment of keloid scars with perioperative interstitial high-dose-rate brachytherapy achieved excellent results, with a recurrence rate of only 4.9% and excellent cosmetic outcomes
Behera 2016 (63)	Carbon dioxide laser	Cryotherapy	Both CO_2_ laser and cryotherapy, when combined with ILTA, were found to be equally effective in treating keloids.
Berman 1997 (116)	Interferon alfa-2b	excision alone/injection with triamcinolone acetonide	Postoperative TAC injections do not reduce the number of keloid recurrences. However, injection of keloid excision sites with IFN-c ~ 2b offers a therapeutic advantage over keloid excision.
Berman 2002 (109)	Imiquimod 5% cream	\	The recurrence rate of excised keloids treated with postoperative imiquimod 5% cream was lower than the recurrence rates previously reported in the literature.
Berman 2020 (157)	Superficial Radiation Therapy	\	SRT with a BED value of 30 Gy delivered to keloidectomy excision sites immediately following excision was well-tolerated and resulted in markedly fewer long-term recurrences than reported following keloidectomy alone.
Bijlard 2017 (148)	High-Dose-Rate Brachytherapy	\	After excision of resistant keloids, high-dose-rate brachytherapy with a biological equivalent dose of approximately 20 Gy is recommended, on the basis of low recurrence and complication rates
Bijlard 2018 (101)	Intralesional cryotherapy	Excision with corticosteroid injections or brachytherapy	Intralesional cryotherapy is inferior to keloid excision followed by brachytherapy for resistant keloids. In primary keloids, intralesional cryotherapy reduced keloid volume
Bischof 2007 (128)	6-MeV electron beam radiotherapy	\	Postoperative electron radiotherapy is well-tolerated and very effective in preventing keloid recurrence
Bonnardeaux 2019 (119)	Surgical excision combined with fully ablative carbon dioxide laser therapy and triamcinolone injections	\	The need for multimodal therapy with combined methods in order to achieve long-term remission
Burusapat 2021 (171)	Immediate triamcinolone acetonide injection	Delayed triamcinolone acetonide injection	Immediate TA injection is an acceptable option for the treatment of auricular keloids. Here, it was associated with a lower recurrence rate than delayed injection and resulted in no complications.
Careta 2013 (120)	Shaving, cryosurgery, and intralesional steroid injection	\	Shaving associated with cryosurgery seems to be a useful treatment for large keloid scars
Carvalhaes 2015 (121)	Triamcinolone injections, surgical resection, and local pressure	\	The combination of infiltration TCN month to 20 mg/mL (1.2 mg to 2.0 mg per mm3 TCN injury), surgical excision, and pressure application device is effective for the treatment of keloid ear lobe.
Chen 2017 (124)	Intralesional injection of diprospan (2 mg betamethasone disodium phosphate and 5 mg betamethasone dipropionate in 1 mL)	Intralesional injection of diprospan with a combination of 0.5 mL 5-fluorouracil (25 mg/mL)/diprospan + 5-FU + 1,064-nm Nd: YAG laser	The combination of diprospan + 5-FU + Nd: YAG was the most efficacious therapy for keloid scars.
Chen 2020 (111)	Continuous tension reduction	\	The technique of continuous tension reduction could be used as an alternative method to prevent keloid recurrence under the condition of without radiotherapy
Chernoff 2007 (189)	Dermatix gel	Silicone gel sheeting	Dermatix is a useful treatment for abnormal scarring.
Cho 2010 (5)	1,064-nm Q-switched Nd: YAG laser with low fluence	\	QS Nd: YAG laser with low fluence may be used for the treatment of keloids and hypertrophic scars.
Choi 2020 (31)	Triamcinolone acetonide intralesional injection	\	TA ILI after intralesional excision can be effective for the management of auricular keloids. A low recurrence rate, similar to that of postoperative radiation therapy, was obtained with an effective surgical procedure and minimal postoperative treatment.
Chopinaud 2014 (98)	Intralesional Cryosurgery	\	Intralesional cryosurgery is an effective treatment for keloids
Cicco 2013 (78)	High-dose-rate interstitial brachytherapy	Low-dose-rate interstitial brachytherapy	Postoperative brachytherapy is an effective treatment for keloids.
Clavere 1997 (152)	Iridium 192 brachytherapy	\	Iridium 192 brachytherapy is an effective treatment for keloids
Copcu 2004 (94)	Combination of Surgery and Intralesional Verapamil Injection	\	Surgical excision with W-plasty or skin grafting and intralesional verapamil injection may be a good alternative in the treatment of keloids.
Dai 2021 (181)	Combination of ablative fractional carbon dioxide laser and platelet-rich plasma	Ablative fractional carbon dioxide laser	PRP is an effective adjunct for AFCL in the treatment of hypertrophic burn scars and that the combination of PRP and AFCL proved to be more useful than AFCL alone.
Darougheh 2007 (163)	Intralesional triamcinolone acetonide	Combination of TAC and 5-fluorouracil	The overall efficacy of TAC + 5-FU was comparable with TAC, but the TAC + 5-FU combination was more acceptable to patients and produced better results.
Daurade 2020 (143)	Combining surgical excision and high-dose-rate brachytherapy	\	Extralesional excision combined with postoperative high-dose-rate brachytherapy seems to be one of the most effective invasive protocols to treat and prevent keloids
Davari 2012 (65)	Pulsed dye laser	Nine weeks after suture removal	The pigmentation and erythema values following early treatment were higher than those following late treatment or no treatment, and the elasticity values were lowest following late treatment.
Davison 2009 (180)	5-FU + steroid with excision	5-FU + steroid without excision/steroid treatment with excision	Combination 5-FU/triamcinolone is superior to intralesional steroid therapy in the treatment of keloids.
Deng 2021 (123)	Intralesional triamcinolone (5 mL 1%) and 5-fluorouracil (0.6 mL 2.5%) injections and Strontium-90 brachytherapy	Intralesional triamcinolone (5 mL 1%) and 5-fluorouracil (0.6 mL 2.5%) injections	Strontium-90 brachytherapy as an adjuvant radiation could effectively reduce the recurrence of small keloids after intralesional triamcinolone and 5-fluorouracil injections.
Dina 2021 (107)	Combined fractional ablative 2,940 nm Er: YAG laser and topical application of steroid cream	Intralesional corticosteroid injection	The use of ablative fractional laser-assisted delivery of topical steroid can offer a safer and better aesthetic treatment option
Disphanurat 2023 (184)	Triamcinolone acetonide-loaded dissolving microneedle patch	Drug-free DMN patch	DMN patches were effective in the transdermal drug delivery of TAC for the treatment of HTSs
Dogahe 2023 (52)	Intralesional triamcinolone (40 mg/mL) and verapamil (2.5 mg/mL)	Intralesional triamcinolone alone (40 mg/mL)	The combination of verapamil and triamcinolone provides a more effective treatment for keloids, thereby highlighting the potential of verapamil in scar reduction
Emad 2010 (84)	Surgical excision and radiotherapy	Cryotherapy and intralesional steroid	Although cryotherapy combined with intralesional steroids was associated with more side effects and higher relapse rates, it could be a good choice for small and newly formed keloids.
Erlendsson 2022 (47)	A pneumatic jet injection with 5-fluorouracil and triamcinolone acetonide	5-FU + TAC	A single PJI injection containing 5-FU and TAC can significantly improve the height and pliability of HTS.
Erol 2008 (14)	Intense Pulsed Light	\	IPL is effective not only in improving the appearance of hypertrophic scars and keloids regardless of their origin, but also in reducing the height, redness, and hardness of scars.
Escarmant 1993 (82)	Iridium 192 interstitial irradiation after surgical excision	\	The effectiveness of the method linking surgical excision and Iridium 192 interstitial irradiation shows the importance of the sterile conditions of the treatment.
Espana 2001 (153)	Bleomycin	\	Bleomycin seems to be a useful treatment for keloid scars
Francesca 2010 (78)	Self-drying silicone gel		Compared with the base cream, self-drying silicone gel has the advantage to dry quickly and allow the subsequent application of sunscreens and other cosmetics.
Francisco 2013 (151)	Combination of bleomycin and triamcinolone acetonide	\	The best results were obtained in keloids over 1 cm^2^ or when divided into 1 cm2 square areas.
Friedman 2020 (166)	Erbium glass, 1,540 nm laser	Not treated	a single presurgical laser treatment of the planned incision site is a simple, safe, and painless strategy to substantially improve the final scar appearance.
Gamil 2018 (188)	Er: YAG laser	Long-pulsed Nd: YAG laser	The Er: YAG laser proved to be a potentially effective and safe modality both in the early and late AKN lesions.
Gamil 2019 (187)	Combined intralesional triamcinolone acetonide with botulinum toxin type A	Intralesional triamcinolone acetonide/intralesional botulinum toxin type A	Combined injection of intralesional steroids with BTX-A appears to be superior to either therapy alone and offer the best benefit of safer and more efficacious response with lesser side effects.
George 2005 (133)	Intralesional 5-fluorouracil	\	Intralesional 5-FU may be effective in the treatment of keloids, but recurrence is common.
Guix 2001 (160)	Interstitial high-dose-rate brachytherapy	\	HDR brachytherapy is an effective treatment for keloid scars. It is well tolerated and does not present significant side effects.
Hafkamp 2017 (161)	Interstitial high-dose-rate brachytherapy	\	Plastic surgery followed by a single dose of 13 Gy HDR brachytherapy resulted in 76% local control of keloid with a relatively good cosmetic outcome.
Hatamipour 2009 (141)	Combined topical silicone and 5Fluorouracil	Topical silicone	The modality of combined 5-FU and topical silicone is a sound approach for the prevention of keloids
Hewedy 2020 (54)	Intralesional triamcinolone acetonide (20 mg/mL) and Platelet rich plasma	TA (20 mg/mL) alone	Combining intralesional PRP with TA could yield cosmetically better outcomes in keloid treatment with a lower incidence of TA-induced side effects, especially atrophy and hypopigmentation.
Hietanen 2018 (39)	Intralesional triamcinolone		TAC and 5-FU injections did not differ in their clinical effectiveness in this randomized study
Hoang 2016 (79)	Interstitial high dose rate brachytherapy	Excision alone/external beam radiotherapy	Post-excision RT shows a significant reduction in keloid recurrence compared to excision alone. While the recurrence control rates are not statistically different between EBRT and brachytherapy, keloids treated with EBRT recurred significantly later than those treated by HDR brachytherapy
Hou 2023 (191)	Punch excision combined with intralesional steroid injection	Intralesional Steroid Injection alone	The combination of punch excision and intralesional steroid injection has a notable therapeutic effect on keloids, shortening the treatment course without evident adverse reactions.
Hye 2015 (70)	Copper bromide laser and intralesional triamcinolone injection (2.5 mg/mL or 5 mg/mL)	\	Three to four treatment sessions were required to reduce scar VSS score by 50% when using the combination treatment of CBL and TA ILI.
Jiang 2015 (73)	Interstitial high-dose-rate brachytherapy	\	Brachytherapy may be advantageous in the management of high-risk keloids or as salvage treatment for failure after external beam therapy.
Jiang 2017 (74)	Interstitial high-dose-rate brachytherapy	\	Brachytherapy may be advantageous in the management of high-risk keloids, even after failure of external beam radiotherapy and other treatment procedures
Kant 2018 (93)	Triamcinolone and verapamil	\	Combined therapy of triamcinolone and verapamil results in overall significant scar improvement with a long-term stable result
Kaushal 2020 (149)	Combined intralesional radiofrequency and intralesional triamcinolone acetonide	Intralesional triamcinolone acetonide alone	Thus, both the studied modalities of treatment produced equal efficacy and safety but with less recurrence in the combined group.
Khalid 2018 (45)	intralesional 5-FU/TAC injections	Radiotherapy	Excision and intralesional5-FU/TAC is an effective treatment for keloids on the ears
Khan 2019 (88)	Intralesional bleomycin	Intralesional triamcinolone	Intralesional bleomycin is more efficacious than intralesional triamcinolone acetonide in the treatment of keloids
Khattab 2019 (145)	Intralesional verapamil alone 2.5 mg/mL.	Combination of PDL and intralesional verapamil alone 2.5 mg/mL	Combination therapy with PDL + intralesional verapamil was superior in efficacy when compared to intralesional verapamil alone, in the treatment of keloids
Khedr 2019 (177)	Nd: YAG laser	Combined intense pulsed light and radiofrequency	Both modalities were successful in the treatment of hypertrophic scars; however, a significant improvement in the clinical and histopathological findings was detected with the E-light method.
Kim 2015 (164)	Intense pulsed light device and intralesional corticosteroid injection	\	IPL + corticosteroid injection can improve the appearance of keloids and hypertrophic scars
Kim 2022 (40)	Pulsed dye laser therapy combined with intralesional triamcinolone injection	\	Early intervention using PDL combined with TAILI could prevent the recurrence or progression of hypertrophic CS scarring after surgical scar removal.
Lee 2008 (53)	Triamcinolone acetonide intralesional injection + Interferon-*α*	Triamcinolone acetonide intralesional injection	Intralesional IFN-伪2b is an effective and safe treatment for keloids.
Lee 2015 (76)	6-MeV electron beam	\	Radiotherapy should be initiated within 72 h of surgical excision.
Leeuwen 2014 (100)	Intralesional Cryotherapy	\	Intralesional cryotherapy for the treatment of keloid scars shows favorable results in terms of reduction of volume and complaints of pain and pruritus. However, no complete eradication was obtained in some cases, and recurring scars were observed.
Levenberg 2020 (144)	Intralesional 5FU and corticosteroids	\	Improved appearance of keloids and symptomatic relief were achieved by intralesional administration of combined 5-fluorouracil and corticosteroid through high-pressure jet injections.
Li 2020 (139)	Intralesional 1,470 nm bare-fiber diode laser	\	The intralesional 1,470 nm bare-fiber diode laser significantly improved hypertrophic and keloid scars based on both subjective and objective analyses and supports this type of laser therapy as a safe and effective minimally invasive treatment option.
Li 2022 (158)	Excision followed by 5-FU and betamethasone intralesional injections	5-FU and betamethasone intralesional injections/excision followed by radiotherapy	Excision followed by intralesional low concentrations of 5-FU (12.5 mg/mL) with betamethasone is a safe and sustainable treatment for keloids, with no significant difference from excision followed by radiotherapy.
Liu 2023 (44)	Combined pulsed dye laser and triamcinolone acetonide	Triamcinolone acetonide	Compared with TAC injection alone, PDL dynamically combined with TAC in the treatment of keloid with post-operative recurrence can shorten the relative cure time, reduce the number of TAC injections, and improve the clinical efficacy.
Luo 2023 (89)	The combined application of bleomycin and triamcinolone	\	The combined application of bleomycin and triamcinolone acetonide can effectively cure keloids and hypertrophic scars.
Lv 2021 (182)	Ablative fractional CO_2_ laser surgery	Conventional surgery	CO_2_-AFL surgery significantly improved sleep quality and reduced pain and pruritus in patients with hypertrophic scars.
Maemoto 2020 (134)	electron beam radiation therapy	\	Multiple lesions and irregular shape were risk factors of keloid recurrence after postoperative electron beam radiotherapy.
Manjunath 2021 (81)	Surgical excision and adjuvant high-dose rate brachytherapy	\	Surgical excision with postoperative radiotherapy is best for preventing recurrence.
Manuskiatti 2021a (58)	Laser + steroid	Laser + petrolatum	Fractional laser monotherapy is an effective treatment for hypertrophic scars, and the application of a topical corticosteroid provides no long-term synergistic effect to fractional laser monotherapy.
Manuskiatti 2021b (58)	Thermomechanical fractional injury-assisted topical corticosteroid (40 mg/mL)	Corticosteroid injection (40 mg/mL)	TMFI-assisted topical corticosteroid delivery is an effective treatment for HTS with a lower risk of adverse effects compared with corticosteroid injection
Martin-Garcia 2005 (108)	Imiquimod 5% Cream	\	Imiquimod 5% cream may prove to be a therapeutic alternative for the prevention of recurrences in excised earlobe keloids.
Meseci 2019 (105)	Topical corticosteroid ointment	Untreated	The clinical outcomes in both groups were similar.
Meymandi 2014 (69)	Intense pulsed light method along with corticosteroid injection	\	Intralesional corticosteroid injection + IPL increases the recovery level of hypertrophic and keloid scars.
Meymandi 2016 (62)	Intense pulsed light	Cryotherapy	Both methods were highly successful in curing scars
Monteiro 2022 (155)	Intralesional 5-Fluorouracil (50 mg/mL)	Combination of 5 fluorouracil (50 mg/mL) with triamcinolone acetonide (40 mg/mL)	5-FU, both as a single agent or in combination with steroids, is equally efficacious in reducing the keloid size. The side effects are fewer with the combination group
Moravej 2022 (87)	Intralesional bleomycin (1.5 mg/mL)	Intralesional triamcinolone (20 mg/mL)	Intralesional bleomycin is effective as triamcinolone in the treatment of keloids and hypertrophic scars; however, bleomycin should be used carefully, due to adverse events such as pain, ulceration, and hyperpigmentation.
Muneuchi 2009 (32)	Intralesional injection of triamcinolone acetonide	\	Intralesional injection of triamcinolone acetonide proved to be a potentially effective and safe modality in keloids.
Neinaa 2021 (114)	Intralesional injection of botulinum toxin type-A (5 IU/injection point)	Intralesional injection of platelet rich plasma (0.1 mL/injection point)/intralesional injection of triamcinolone acetonide (20 mg/session)	Both BTX-A and PRP could yield a chance for cosmetically better outcomes in keloid treatment than conventional TAC injection.
Nishi 2022 (104)	Combination of cryotherapy with intralesional corticosteroid	A combination of fractional CO_2_ laser followed by topical corticosteroids	Both regimens showed excellent responses with minimum recurrence rates
Nor 2016 (110)	Either daily topical clobetasol propionate 0.05% cream under occlusion with a silicone dressing	Monthly intralesional triamcinolone injection	Clobetasol propionate 0.05% cream under occlusion with silicone dressing is equally effective and has fewer adverse effects compared to IL triamcinolone.
Noruri 2003 (176)	585-nm pulsed dye laser	Not treated	The 585-nm PDL is effective and safe in improving the quality and cosmetic appearance of surgical scars in skin types I–IV starting on the day of suture removal.
Ogawa 2002 (165)	Electron beam radiation therapy	\	A keloid with a high risk of recurrence should be treated with electron beam radiation doses and posttreatment self-management
Ogawa 2014 (169)	Surgical excision and radiotherapy	\	We recommend surgical removal together with postsurgical radiation therapy consisting of 15-Gy irradiation administered in three fractions over 3 days.
Ollstein 1981 (80)	Combined surgical excision and immediate X-ray therapy	\	Intralesional excision combined with immediate X-ray therapy is effective in treating keloids
	Intralesional steroid injections (20 mg/mL)	\	Surgical excision followed by intra- and postoperative intralesional steroid injection therapy has provided reasonable success at preventing recurrence
Payapvipapong 2014 (183)	Intralesional triamcinolone acetonide (10 mg/mL)	Intralesional bleomycin (1 mg/mL)	No skin atrophy was detected in this study. Intralesional bleomycin is a safe and effective treatment for keloids and hypertrophic scars.
Qiao 2017 (97)	Surgery combined with lucortriticod and electron irradiation group		In the treatment of ear scar, the efficacy of Surgery combined with lucortriticod and electron irradiation group > Surgery combined with local injection of glucocorticoid group = Surgery combined with superficial X-ray group > Surgery group
Ragoowansi 2002 (171)	Surgical excision and immediate single-fraction radiotherapy	\	Extralesional excision of keloid followed by early, single-fraction, postoperative radiotherapy is both simple and effective in preventing recurrence at excision sites.
Ramadan 2021 (90)	Pulsed Nd: YAG laser and intralesional bleomycin	Pulsed Nd: YAG laser only	Long-pulsed Nd-YAG laser combined with intralesional bleomycin could be a promising way for the treatment of keloids or hypertrophic scars.
Rasaii 2018 (49)	Intralesional triamcinolone (40 mg/mL) in combination with botulinum toxin A (20 mg/mL)	Intralesional triamcinolone (40 mg/mL) alone	Intralesional injection of triamcinolone and BTA has been found to have a similar effect on keloidal cosmesis to triamcinolone alone.
Reinholz 2020 (41)	Intralesional 5-fluorouracil (50 mg/mL) in combination with triamcinolone acetonide (40 mg/mL)	\	The results of this study confirm the efficacy and safety of the use of a combination of 5-FU and TAC in a 3:1 ratio in keloids based on objective measurements. Treatments were well tolerated and demonstrated stable results at 12-month FU.
Sabry 2019 (64)	CO_2_ laser and topically applied 5-FU	CO_2_ laser and topically applied verapamil hydrochloride/ablative fractional CO_2_ laser monotherapy	Combined fractional CO_2_ laser and topical 5-FU or verapamil hydrochloride offer a safe therapy for HTSs and keloids
Sabry 2020 (150)	Combined laser and intralesional injection of botulinum toxin A	Intralesional injection of botulinum toxin A	Laser-assisted delivery of Botox is a novel modality of treatment with minimal side effects and promising efficacy.
Sadeghinia 2012 (35)	Intralesional triamcinolone acetonide	5-FU tattooing	5-FU tattooing was more effective than intralesional TAC for treating keloids.
Saha 2012 (142)	5-FU (50 mg/mL)	Triamcinolone acetonide (40 mg/mL)	Triaclcinolone is a better-tolerated and less toxic alternative to 5-FU in the management of keloids
Saki 2019 (36)	Intralesional triamcinolone acetonide	Intralesional Verapamil	Verapamil is not as effective as triamcinolone in the treatment of keloids.
Saray 2005 (175)	Dermojet injections of bleomycin (1.5 IU/mL)	\	Intralesional jet injection of bleomycin is an effective and safe method of treating keloids and hypertrophic scars that are unresponsive to intralesional steroid therapy.
Seo 2011 (117)	Topical and intralesional mitomycin C (1 mg/mL)	\	Topical application of mitomycin C was safe and effective for the treatment of keloids and HTS
Shaarawy 2014 (113)	Intralesional botulinum toxin type A	Intralesional steroid	The current work establishes the possible effective and safe “off-label” use of BTA in such an indication.
Sharma 2007 (46)	Combination of liquid nitrogen and intralesional triamcinolone acetonide (5 mg/mL)	Liquid nitrogen alone	the combination therapy is a better modality of treatment of small keloids compared with liquid nitrogen cryosurgery alone
Sharma 2021 (51)	Intralesional 5-FU and triamcinolone acetonide	Combination of intralesional bleomycin and triamcinolone acetonide	A greater improvement in the signs and symptoms of keloids (with respect to cosmetic problems, restriction of movement, and tenderness) was observed in the patients treated with a combination of intralesional bleomycin and triamcinolone acetonide compared to those treated with a combination of intralesional 5-FU and triamcinolone acetonide
Shen 2015 (172)	Hypofractionated electron-beam radiation	\	Early postoperative radiotherapy with limited hypofractionation could be a good choice for keloid treatment
Shin 2019 (43)	Combination of non-ablative fractional laser and intralesional triamcinolone injection	Intralesional triamcinolone injection	Combination therapy with non-ablative fractional laser and intralesional steroid injection showed better results for the treatment of hypertrophic scars and keloids with fewer treatment sessions, better patient satisfaction, and longer remission periods.
Son 2014 (57)	578 nm copper bromide laser combined with intralesional corticosteroid injection	\	The adjunctive use of 578 nm copper bromide laser decreased the telangiectatic side effects of an intralesional corticosteroid injection by reducing the vascular components of scars.
Son 2020 (77)	A single dose of low-energy superficial X-ray radiation	\	A single 8Gy dose of superficial 50 kV radiation delivered an average of 34 days following keloid excision maybe sufficient to minimize recurrence.
Song 2014 (170)	Single-fraction radiotherapy	\	Surgical excision of the keloid, followed by immediate, single-fraction, high-dose radiotherapy, is both safe and effective in preventing recurrence of therapy-resistant keloids
Song 2018 (112)	Hyperbaric oxygen therapy	Surgical excision and radiotherapy	Adjunctive HBOT effectively reduces the keloid recurrence rate after surgical excision and radiotherapy by improving the oxygen level of the tissue and alleviating the inflammatory process
Song 2018 (112)	Intralesional triamcinolone acetonide injection	\	The treatment efficacy was better when applied during the static stage of pathological scarring rather than the early stage
Srivastava 2018 (48)	Intralesional triamcinolone acetonide (40 mg/mL) and 5-fluorouracil (50 mg/mL)	Intralesional triamcinolone acetonide (40 mg/mL)/intralesional 5-fluorouracil (50 mg/mL)	TAC, 5FU, and their combination are all effective in keloid scars. A combination of TAC + 5FU seems to offer the balanced benefit of faster and more efficacious response with lesser adverse effects when compared to individual drugs
Sruthi 2017 (75)	Single-fraction radiation	\	Radiation as an adjuvant therapy in the postoperative period within 48 h is a cosmetically acceptable, safe, painless, cost-effective treatment with good patient compliance to prevent keloid recurrence.
Stephanides 2011 (162)	Intralesional triamcinolone (10 mg or 40 mg/dl) and pulsed dye laser	\	Pulsed-dye laser with or without intralesional triamcinolone is a moderately effective treatment of keloid scars with a very good side-effect profile and high patient satisfaction.
Stern 1989 (66)	Carbon dioxide laser excision	\	The carbon dioxide laser excision can not effectively cure Earlobe Keloids.
Stewart 2006 (96)	The combination of surgical excision with the application of topical mitomycin-C	\	The topical application of mitomycin-C is an effective therapy for preventing keloid recurrence in the head and neck.
Stromps 2013 (186)	Intralesional cryotherapy combined with postoperative silicone gel sheeting	Intralesional cryotherapy alone	The use of combined intralesional cryosurgery followed by the application of silicone gel sheeting to treat refractory keloids.
Sunil 2018 (185)	Fractional CO_2_ laser	Intralesional triamcinolone (40 mg/mL)/intralesional verapamil (2.5 mg/ mL)	fractional CO_2_ laser and verapamil are as efficient as triamcinolone acetonide (TAC) for treating keloids, except it takes longer for the laser and verapamil to act compared to TAC.
Suwanchinda 2022 (115)	Cold atmospheric-pressure plasma	untreated	CAP technology could be considered an alternative treatment for keloids, offering mild-to-moderate improvement with minimal side effects.
Tawaranurak 2022 (42)	Treated with fractional CO_2_ laser + topical TA	Intralesional TA	The combination of a fractional CO_2_ laser with topical TA was an alternative option for the treatment of keloids without any adverse effects.
Tawfic 2020 (71)	Combined: Fractional and Nd YAG	Nd: YAG alone/Fractional laser alone	Long-pulsed Nd: YAG laser is an effective and safe treatment for hypertrophic scars and keloids. Fractional CO_2_ laser yields better improvement in hypertrophic scars, while in keloids, both fractional CO_2_ and Nd: YAG lasers achieve comparable improvement. Combination in the same session did not add significant additional benefit, and the side-effect profile was higher.
Tawfik 2019 (190)	Combined 5-fluorouracil and fractional erbium YAG laser	Topical 5-fluorouracil cream	Treatment of severe HTS with combined 5-FU and ablative fractional erbium YAG laser is more effective than 5-FU alone.
Tsai 2019 (56)	Combination of 1,064-nm neodymium-doped yttrium aluminum garnet laser and steroid tape	Steroid Tape	Nd: YAG laser treatment effectively decreased the total treatment time of hypertrophic cesarean-section scars.
Viani 2009 (178)	Strontium 90 brachytherapy	\	Excision plus Sr-90 brachytherapy is effective in the eradication of keloids.
Wang 2020 (67)	Combined ultrapulse fractional carbon dioxide laser and topical triamcinolone	\	Combination keloid therapy using UFCL and tropical triamcinolone has overall significant improvement and low recurrence rate with a long-term stable results.
Wang 2020 (179)	Combined surgical excision and electron external beam radiation	\	Surgical excision followed by immediate adjuvant radiation therapy for keloids provides excellent local control of disease as well as cosmetic appearance.
Wen 2021 (140)	Hypofractionated radiotherapy	\	A postoperative hypofractionation with radiation dose of 20Gy in 5 fractions may be effective, easy to accept and safe for keloid patients.
Weshahy 2012 (154)	Combined intralesional cryosurgery and intralesional steroid injection	\	Combined Intralesional cryosurgery and intralesional steroid injection seems to be a useful treatment for keloids scars
Weshay 2015 (173)	Combination of Radiofrequency and intralesional steroids (10 mg/mL)	\	Radiofrequency tissue volume reduction combined with IL steroid is an effective treatment modality for keloids. It is an easy procedure with acceptable cosmetic outcome and less rate of recurrence.
Wittenberg 1999 (59)	585-nm flashlamppumped pulsed-dye laser	Silicone gel sheeting	The improvements in scar sections treated with silicone gel sheeting and pulsed-dye laser were no different from those in control sections.
Yii 1996 (147)	Cynthaskin and topical steroid	\	All patients achieved symptomatic relief of itch and pain
Yosipovitch 2009 (103)	Cryotherapy and steroid injection	Cryotherapy alone/Steroid injection	Combined injection of intralesional steroids with cryotherapy appears to be superior to other current modalities.
Zawahry 2015 (192)	Fractional CO_2_ laser	Untreated	Fractional CO_2_ laser is a safe and effective modality for the treatment of hypertrophic burn scars, with improvement achieved
Zouboulis 2020 (102)	Combined liquid nitrogen contact cryosurgery with intralesional corticosteroids	Liquid nitrogen contact cryosurgery	Cryosurgery without and with intralesional corticosteroids is effective and safe on young,
Zhou 2023 (193)	Ablative fractional carbon dioxide laser +1 g triamcinolone external application	Ablative fractional carbon dioxide laser + 40 mg/mL triamcinolone Intralesional injection	Using the ablative fractional carbon dioxide laser followed by different topical triamcinolone delivery methods is effective and safe for thicker hypertrophic scar improvement.
Zhang 2023 (194)	Intervention of the experimental group	Intervention of the controlled group	The combination of CO_2_ fractional laser and narrowband IPL efficiently improved the appearance and profile of hypertrophic scars, offering a comprehensive and reliable approach for scar therapy.
Li 2024 (195)	Combined Intense Pulsed Light (IPL) with Fractional CO_2_-Laser	Fractional CO_2_-Laser	Keloid-cross-flap surgery could provide favorable morphologic repair of the auricular keloid, and postoperative superficial radiotherapy shows higher compliance and lower recurrence rate compared to compression treatment.
Pazyar 2024 (196)	Intralesional cryosurgery	Contact cryosurgery	It seems that the injection of vitamin D at the site of the lesion helps to treat it, but the effectiveness of the usual triamcinolone treatment was still higher
Khan 2025 (85)	Intralesional verapamil (2.5 mg/mL)	Intralesional corticosteroids (40 mg/mL)	Surgical excision and radiotherapy can not effectively cure Keloids.
Jiang 2024 (197)	Intralesional triamcinolone (40 mg/mL)	\	Fractional CO_2_ laser followed by 5-ALA PDT is a promising method for treating keloids.
Hu 2023 (83)	A triple therapy comprising the use of steroid injections and cream (triamcinolone acetonide), surgery, and silicone gel strip/sheet pressure application	\	Surgical resection, super-subtraction sutures, and superficial radiotherapy are treatment methods with short courses, low recurrence rates, and good safety profiles.
Yang 2025 (198)	Iridium-192 high-dose rate surface mold brachytherapy	\	The sequentially comprehensive treatment based on surgery has a significant curative effect, as well as a low recurrence rate
Park 2024 (199)	Intralesional verapamil hydrochloride (concentration 2.5 mg/mL)	Intralesional triamcinolone acetonide (concentration 40 mg/mL)	Dual therapy involving TAC injection and Nd: YAG laser treatment was more effective than TAC injection alone for managing keloid scars after surgery.
Harsono 2023 (30)	Intralesional triamcinolone (concentration 40 mg/mL)	intralesional 5-fluorouracil (concentration 250 mg/5 mL)/intralesional verapamil (concentration 2.5 mg/mL)/intralesional platelet-rich plasma (2.4 mL)	Intralesional injection of umbilical cord Mesenchymal stem cells is effective in the eradication of keloids.
Qiu 2025 (200)	Fractional CO_2_ laser with intralesional steroid	Intralesional steroid alone	The findings indicate that laser-assisted drug delivery using MFT with a CO_2_ laser demonstrates significant clinical efficacy, a low recurrence rate, and an absence of serious adverse reactions in treating hypertrophic scars.
Aristides 2024 (201)	Fractional carbon dioxide laser with intralesional 5-fluorouracil (50 mg/mL)	\	A combination of two ablative laser delivery modes within a single laser platform provided for effective and safe keloid management and left patients highly satisfied.
Lim 2024 (118)	Combination of surgical excision and perioperative corticosteroid injection (40 mg/mL)	\	This demonstrates the use of transdermal gene-silencing technology in scar inhibition and that siRNA microneedle patches can be effective and safe in reducing scar tissue formation

The treatment options for keloid and hypertrophic scar included in the study could be roughly divided into monotherapy (MT) and combination therapy, of which 80 were monotherapy studies, and 82 were combination therapy (CT) studies (72 studies with double combined therapy and 10 studies with triple combined therapy). From the classification of intervention methods, the treatment methods we retrieved included: Corticosteroid therapy [e.g., triamcinolone acetonide (TA)] (MT = 11; CT = 40), optical therapy (IPL or laser) (MT = 17; CT = 29), radiation therapy (MT = 22; CT = 12), antipyrimidine therapy [5-fluorouracil (5-FU)] (MT = 2; CT = 10), anticancer drug therapy (bleomycin) (MT = 4; CT = 2), calcium channel blocker therapy (verapamil) (MT = 3; CT = 3), surgical resection (MT = 0; CT = 15), topical therapy (gel or cream) (MT = 9; CT = 2), cryosurgery (MT = 4; CT = 5), botulinum toxin (MT = 2; CT = 2), interferon (MT = 1; CT = 1) and other physical therapy (MT = 3; CT = 0). Furthermore, two studies investigated two newer monotherapy approaches: intralesional vitamin D injection (29) and intralesional injection of umbilical cord mesenchymal stem cells (30).

### Study quality

**Figure 2:**
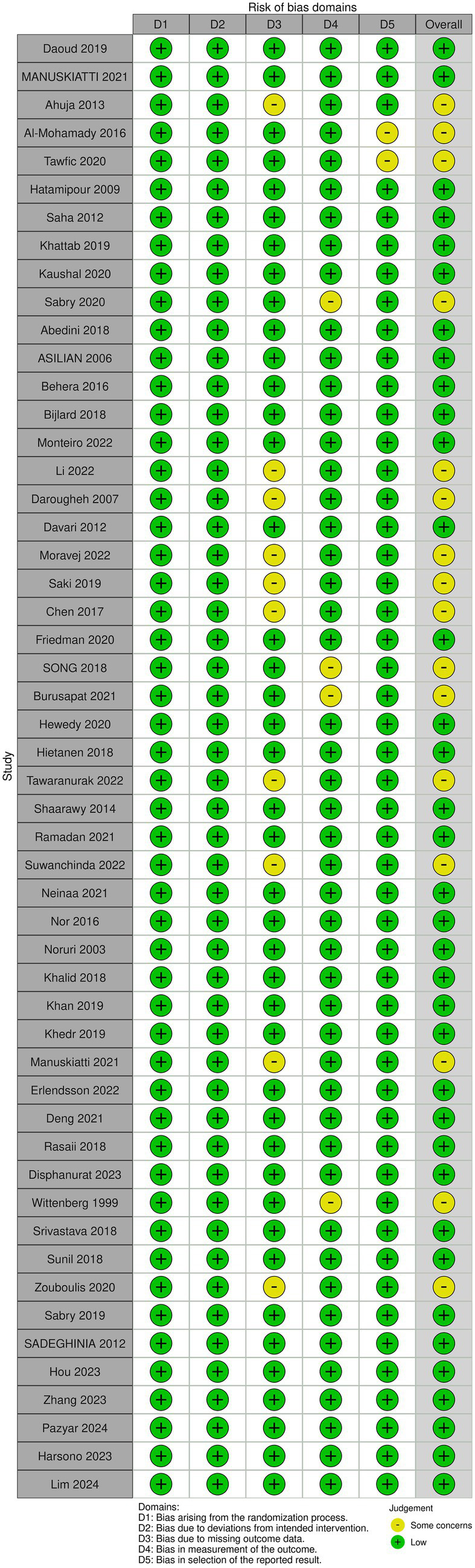
Quality assessment result for included randomized controlled trials (RCT) studies.

Quality assessment of 52 RCTs using RoB2 classified 36 studies as having low risk of bias and 16 studies as having some concerns. Ten of the 16 studies raised concerns due to a substantial proportion of missing outcome variables. Four studies were classified as at risk of bias due to the subjective nature of their outcome measures, while two studies demonstrated potential selective bias in outcome reporting (Supplementary Table 2 and Figure 2).

Of 110 nonrandomized studies assessed with MINORS, 34 were comparative, and 75 were uncontrolled. Among comparative studies, 31 were rated as high quality (≥18) and 3 as moderate quality (14–17). All 75 uncontrolled studies were rated as high quality (≥12). The limitations of controlled studies mirror those of single-arm studies, including improper selection of study endpoints and short follow-up periods (Supplementary Table 3).

### Hormone monotherapy

TA is a topical (skin) steroid drug used to treat different skin conditions (31). TA is currently considered to be the primary intrafocal treatment for keloid and hypertrophic scars (31). The study demonstrated that local injections of TA alone proved to be a potentially effective and safe treatment (32, 33). Eleven of the studies included in this review involved TA monotherapy. Eight of the 11 studies confirmed the efficacy of TA monotherapy, and one small sample study also confirmed that TA has an anti-recurrence effect comparable to radiotherapy (31) and is very effective in the treatment of keloids in children (33). Studies of the timing of administration have shown that the treatment efficacy was better when applied during the static stage of pathological scarring rather than the early stage (34). Advances in delivery, such as TA-loaded dissolving microneedle patches, showed promising results (35) (Table 3).

**Table 3:** Hormone monotherapy.

Study	Intervention of the experimental group	Intervention of the controlled group	Outcomes	Indicators of outcome (experimental group)	Indicators of outcome (controlled group)
Choi 2020 (31)	Triamcinolone acetonide intralesional injection	\	Recurrence rate	0.05	\
Darougheh 2007 (163)	Intralesional triamcinolone acetonide	Combination of TA and 5-fluorouracil	Efficacy	0.2	0.55
Muneuchi 2009 (32)	Intralesional injection of triamcinolone acetonide	\	Efficacy	0.55	\
Saki 2019 (36)	Intralesional Triamcinolone Acetonide	Intralesional Verapamil	VSS	3.1 ± 1.85	0.21 ± 0.56
Acosta 2016 (33)	Intralesional Triamcinolone (40 mg/mL)	\	Size reduction	82.7%	\
Hietanen 2018 (39)	Intralesional triamcinolone		Efficacy	60%	46%
Song 2018 (112)	Intralesional triamcinolone acetonide injection	\	Efficacy	78.2%	\
Albalat 2021 (37)	Intralesional triamcinolone (concentration 40 mg/mL)	Intralesional 5-fluorouracil (concentration 250 mg/5 mL)/intralesional verapamil (concentration 2.5 mg/mL)/intralesional platelet-rich plasma (2.4 mL)	Efficacy	0.75	55%/80%/72%
Payapvipapong 2014 (183)	Intralesional triamcinolone acetonide (10 mg/mL)	Intralesional bleomycin (1 mg/mL)	POSAS improvement rate	0.2754	0.3812
Disphanurat 2023 (184)	Triamcinolone acetonide-loaded dissolving microneedle patch	Drug-free DMN patch	Mean change of POSAS	−7.2(−12.94, −1.46)	--5.2(−12.17, 1.77)
SADEGHINIA 2012 (35)	Intralesional triamcinolone acetonide	5-FU tattooing	Efficacy	\	\

However, results from six comparative studies showing the efficacy of TA alone remain controversial. The results of Darougheh’s study showed that although TA alone was as effective as the combination of 5-FU, the combination treatment was more acceptable to patients and therefore achieved better results (3). A small sample study published in 2019 showed that verapamil was less effective than TA in the VSS score for the treatment of keloids (36). Conversely, a 2021 cohort study of 160 patients concluded that although TA monotherapy had no significant side effects, plaque verapamil was the most effective treatment (patient observer scar assessment scale (POSAS) 29 ± 10.91 vs. 36 ± 12.74) (37). Such differences may be due to differences in outcome measures, with VSS assessing thickness and POSAS using surface area, which also takes into account the patient’s perception of symptoms (38). Second, two RCT studies with similar publication years and similar sample sizes reached opposite conclusions about the efficacy of TA and 5-FU. The results of the Hietanen 2018 (39) study showed no statistically significant difference in 6-month response rates between the 5-FU and TA groups (46 and 60%, respectively). Sadeghinia 2012 (35) found that at 44 weeks of follow-up, erythema, pruritus, height, surface, and induration were improved in both groups, but the improvement was more significant in the 5-FU group (*p* < 0.05). This may be due to the difference in the length of follow-up, and the efficacy and repeatability of TA monotherapeutics need to be further investigated.

### Hormone combination therapy

There were 38 of the included studies that discussed the efficacy of corticosteroid hormones in the treatment of keloid and hypertrophic scars. There were 36 of the included studies that discussed the efficacy of corticosteroid hormones in the treatment of keloid and hypertrophic scars. Similar to hormonal monotherapy regimens, TA was the corticosteroid of choice in most of the studies (*n* = 21), and the remaining 17 studies used a combination regimen of intralesional steroid injections (Table 4).

**Table 4:** Hormone combination therapy.

Study	Intervention of the experimental group	Intervention of the controlled group	Outcomes	Indicators of outcome (experimental group)	Indicators of outcome (controlled group)
manuskiatti 2021 (58)	Fractional Laser + steroid	Laser + petrolatum	Reduction in hypertrophic scars thickness	0.66 ± 0.39	0.69 ± 0.36
Son 2014 (57)	578 nm copper bromide laser combined with intralesional corticosteroid injection	\	PGA	2.08	\
Levenberg 2020 (144)	Intralesional 5FU and corticosteroids	\	VSS improvement rate	0.53	\
Yii 1996 (147)	Cynthaskin and topical steroid	\	Efficacy	0.7562	\
Francisco 2013 (151)	Combination of bleomycin and triamcinolone acetonide	\	Efficacy	0.9729	\
Weshahy 2012 (154)	Combined intralesional cryosurgery and intralesional steroid injection	\	Reduction area of Keloids	0.935	\
Meymandi 2014 (69)	Intense pulsed light method along with corticosteroid injection	\	Efficacy	89.1%	\
Stephanides 2011 (162)	Intralesional triamcinolone (10 mg or 40 mg/dL) and pulsed dye laser	\	Efficacy	76%	\
Kim 2015 (164)	Intense pulsed light device and intralesional corticosteroid injection	\	Improvement in MVSS	0.981	\
Kim 2022 (40)	Pulsed dye laser therapy combined with intralesional triamcinolone injection	\	VSS	3.11 ± 1.52	\
Hye 2015 (70)	Copper bromide laser and intralesional triamcinolone injection (2.5 mg/mL or 5 mg/mL)	\	VSS	\	\
Aljodah 2021 (167)	Combination of surgical excision and perioperative corticosteroid injection (40 mg/mL)	\	Recurrence rate	9.6%	\
Park 2012 (168)	Intralesional steroid injections (20 mg/mL)	\	Recurrence rate	23.5%	\
Reinholz 2020 (41)	Intralesional 5-fluorouracil (50 mg/mL) in combination with triamcinolone acetonide (40 mg/mL)	\	Improvement in POSAS	39%	\
Hewedy 2020 (54)	Intralesional triamcinolone acetonide (20 mg/mL) and platelet rich plasma	TA (20 mg/mL) alone	Patients’ satisfaction	65%	55%
Weshay 2015 (173)	Combination of radiofrequency and intralesional steroids (10 mg/mL)	\	Volume reduction/Recurrence rate	95.42 ± 7.62/10%	\
Tawaranurak 2022 (42)	Treated with fractional CO_2_ laser + topical triamcinolone	Intralesional TA	Efficacy/Recurrence rate	63.6%/9.1%	72.7%/18.2%
Luo 2023 (89)	The combined application of bleomycin and triamcinolone	\	Recurrence rate	6.9–7.9%	\
Khalid 2018 (45)	Intralesional 5-FU/TA injections	Radiotherapy	Efficacy	0.7333	0.4333
Manuskiatti 2021 (58)	Thermomechanical fractional injury-assisted topical corticosteroid (40 mg/mL)	Corticosteroid injection (40 mg/mL)	VSS	2.28 ± 1.7	2.52 ± 1.83
Erlendsson 2022 (47)	A pneumatic jet injection with 5-fluorouracil and triamcinolone acetonide	5-FU + TA	Improvement in VSS	55%	25%
Nishi 2022 (104)	Combination of cryotherapy with intralesional corticosteroid	A combination of fractional CO_2_ laser followed by topical corticosteroids	MSS	15.67	12.56
Sharma 2021 (51)	Intralesional 5-FU and triamcinolone acetonide	Combination of intralesional bleomycin and triamcinolone acetonide	Efficacy	93.33%	93.33%
Yosipovitch 2009 (103)	Cryotherapy and steroid injection	Cryotherapy alone/Steroid injection	Thickness, pain, and itch	7.7 ± 4.2 mm	3.1 ± 2.2 mm/−0.25 ± 1.2 mm
Rasaii 2018 (49)	Intralesional triamcinolone (40 mg/mL) in combination with botulinium toxin A (20 mg/mL)	Intralesional triamcinolone (40 mg/mL) alone	VSS	6.43 ± 0.37	5.52 ± 0.32
Srivastava 2018 (48)	Intralesional triamcinolone acetonide (40 mg/mL) and 5-fluorouracil (50 mg/mL)	Intralesional triamcinolone acetonide (40 mg/mL)/intralesional 5-fluorouracil (50 mg/mL)	VSS	0.3 ± 0.47	0.61 ± 0.45/0.2 ± 0.41
Zouboulis 2020 (102)	Combined liquid nitrogen contact cryosurgery with intralesional corticosteroids	Liquid nitrogen contact cryosurgery	Efficacy	90%	83.3%
Alexander 2018 (55)	Fractional CO_2_ laser with intralesional steroid	Intralesional steroid alone	Efficacy	\	\
Gamil 2019 (187)	Combined intralesional triamcinolone acetonide with botulinum toxin type A	Intralesional triamcinolone acetonide/intralesional botulinum toxin type A	Photographic documentation, the SBSES score, and CDU results	0.673 ± 0.466	1.18 ± 1.44/1.63 ± 2.19
Dogahe 2023 (52)	Intralesional triamcinolone (40 mg/mL) and verapamil (2.5 mg/mL)	Intralesional triamcinolone alone (40 mg/mL)	VSS	1.5 ± 0.6	4.1 ± 1.9
Shin 2019 (43)	Combination of non-ablative fractional laser and intralesional triamcinolone injection	Intralesional triamcinolone injection	Recurrence rate	35.3%	38.1%
Sharma 2007 (46)	Combination of liquid nitrogen and intralesional triamcinolone acetonide (5 mg/mL)	Liquid nitrogen alone	Efficacy	100%	86.7%
Tsai 2019 (56)	Combination of 1,064-nm neodymium-doped yttrium aluminum garnet laser and steroid tape	Steroid Tape	Time of total JSS score <3	16.9 months	24.3 months
Hou 2023 (191)	Punch excision combined with intralesional steroid injection	Intralesional steroid injection alone	VSS and POSAS	\	\
Liu 2023 (44)	Combined pulsed dye laser and triamcinolone acetonide	Triamcinolone Acetonide	VSS	4	5
Lee 2008 (53)	Triamcinolone acetonide intralesional injection + Interferon-α	Triamcinolone acetonide intralesional injection	Efficacy	81.6%	66%
Zhou 2023 (193)	Ablative fractional carbon dioxide laser +1 g triamcinolone external application	Ablative fractional CO_2_ laser + 40 mg/ml triamcinolone intralesional injection	VSS	5.94 ± 24.07	19.77 ± 21.25
Park 2024 (199)	Intralesional triamcinolone + ND: YAG laser	Intralesional triamcinolone	MVSS, POSAS, recurrence rate, and overall patient satisfaction	MVSS: 3.5 ± 0.3; POSAS: 1.9 ± 0.3; Recurrence rate: 4.35%; Overall patient satisfaction:4.5 ± 0.5	MVSS: 3.7 ± 0.5; POSAS: 2.7 ± 0.4; Recurrence rate: 12.5%; Overall patient satisfaction:4.4 ± 0.6

The 21 studies that examined the TA combination included 6 single-arm studies and 11 controlled studies. The results of single-arm studies were consistent, showing that the TA combined regimen could effectively treat keloid and hypertrophic scar. These programs include TA in combination with bleomycin (*n* = 2), TA in combination with pulsed dye laser (PDL)/copper bromide laser (*n* = 5), and TA in combination with 5-FU (*n* = 1). Among them, Kim 2022 (40) confirmed that early intervention of PDL combined with TA can prevent recurrence or progression after hypertrophic scar resection. The results of the 5-FU combined TA cohort study measured the ratio of drug combinations, and the combination treatment was well tolerated at a ratio of 3:1 and stable at 1 year (41). The selection of TA combination regimen in controlled studies is relatively rich, and all studies confirm the superiority of the combination regimen over monotherapy. The included TA combination regimen includes laser combination (fractional CO_2_ laser, non-ablative fractional laser, PDL) (*n* = 3) (42–44), surgical combination (excision and freezing) (*n* = 2) (45, 46), and 5-FU combination (*n* = 2) (47, 48), botulinum toxin type A combination (*n* = 2) (49, 50), bleomycin combination (*n* = 1) (51), verapamil combination (*n* = 1) (52), interferon-*α* combination (*n* = 1) (53), and platelet-rich plasma combination (*n* = 1) (54).

Among the 17 other corticosteroid injection regimens included in the analysis were: surgical combination (excision and freezing) (*n* = 7), optical combination (laser and IPL) (*n* = 6), 5-FU combination (*n* = 1), Cynthaskin combination (*n* = 1), thermomechanical fractional injury-assisted (*n* = 1), and radiofrequency (*n* = 1). Heterogeneity of the study conclusions was observed in the hormone combination laser study. Three of the four combined laser studies confirmed the efficacy of the fractional CO_2_ laser (55), the 1,064 nm neodymium-doped yttrium aluminum garnet laser (Nd: YAG) (56), and the 578 nm copper bromide laser combined with corticosteroid hormones (57), respectively. Son 2014 also proposed that laser therapy reduced the telangiectasia side effects of intrafocal corticosteroid injections (57). However, the results of the RCT study published in 2021 with a follow-up period of 6 months showed that local corticosteroids and monotherapy with fractional laser (4 times, 28 J/cm^2^, pulse width 300 us, and 5% density) have no long-term synergistic effect on the thickness reduction of abdominal hypertrophic scars (58). This disagreement may be due to differences in laser mode, but it should also be considered in clinical practice that laser therapy may be a better choice than intralesional corticosteroid injection for patients with thicker scars.

### Optical monotherapy

The number of studies on optical monotherapy included was 17, and the study time span was large, from 1989 to 2021. With the update of instruments, the treatment depth, treatment density, and energy density of optical therapy continued to increase. The pulse energy density has increased from 6.5 to 8.0 J/cm2 in 1999 (59) to 20–150 mJ/cm^2^ in 2021 (60). However, the available optical therapy methods have considerable limitations, mainly concentrated in all types of laser therapy [pulsed dye laser (PDL), bare-fiber diode laser, Nd: YAG laser, and carbon dioxide laser (CO_2_ laser)] and IPL treatment (14). These optical therapies have been reported to have significant effects on the long-term improvement in the appearance of hypertrophic scars. The results of controlled studies confirmed that laser therapy for keloid and hypertrophic scars performed as well as cryosurgery (61–63), verapamil (64), radiofrequency therapy (37), and silicone gel sheeting (59) in terms of efficacy and anti-recurrence. The pigmentation and erythema values improved significantly after early treatment, and the elasticity values were the lowest after late treatment (65). The only concern was the results of a single-arm study published in 1989 (66) that showed that carbon dioxide laser excision, in either continuous or 0.2 s pulse mode with an average intensity of 12 W, was not effective in treating earlobe keloids. Given the technical capacity and level of evidence at the time of publication, we believe that such results are of limited significance as a guide to clinical practice today (Table 5).

**Table 5:** Optical monotherapy.

Study	Intervention of the experimental group	Intervention of the controlled group	Outcomes	Indicators of outcome (experimental group)	Indicators of outcome (controlled group)
Al-Mohamady 2016 (138)	595-nm Pulsed dye laser	Nd: YAG laser	VSS improvement rate	0.5514	0.6544
Li 2020 (139)	Intralesional 1,470 nm bare-fiber diode laser	\	VSS improvement rate	0.42	\
Cho 2010 (5)	1,064-nm Q-switched Nd: YAG laser with low fluence	\	The modified Vancouver General Hospital Burn Scar Assessment score	5.9	\
Behera 2016 (63)	CO_2_ Laser	Cryotherapy	Efficacy	0.5278	0.6296
Erol 2008 (14)	Intense pulsed light	\	Efficacy	0.925	\
Davari 2012 (65)	Pulsed dye laser	Nine weeks after suture removal	Elasticity values	0.357	0.246
Friedman 2020 (166)	Erbium glass, 1,540 nm laser	Not treated	POSAS	1.55–3	2.28–4.42
Noruri 2003 (176)	585-nm pulsed dye laser	Not treated	Improvement in the VSS	0.54	0.1
Khedr 2019 (177)	Nd: YAG laser	Combined intense pulsed light and radiofrequency	VSS	3.75 ± 1.09	2.08 ± 0.86
Stern 1989 (66)	CO_2_ laser excision	\	Recurrence rate	73.91%	\
ANG 2013 (61)	CO_2_ laser ablation	Cold steel debulking surgery	Recurrence rate	100	100
Lv 2021 (182)	Ablative fractional CO_2_ laser surgery	Conventional surgery	VAS	3.57 ± 1.36	5.82 ± 1.86
Meymandi 2016 (62)	Intense pulsed light	Cryotherapy	Efficacy	89.3%	91.5%
Wittenberg 1999 (59)	585-nm flashlamp-pumped pulsed-dye laser	Silicone gel sheeting	blood flow, volume, and pruritus	\	\
Sunil 2018 (185)	Fractional CO_2_ laser	Intralesional triamcinolone (40 mg/mL)/intralesional verapamil (2.5 mg/ mL)	VSS	0.25 ± 44	0 ± 0/0.05 ± 0.22
Gamil 2018 (188)	Er: YAG laser	Long-pulsed Nd: YAG laser	efficacy	91.8%	88%
Zawahry 2015 (192)	Fractional CO_2_ laser	Untreated	POSAS	20.27 ± 14.7	15.42 ± 8.7

### Optical combination therapy

Research on optical combination therapy has mostly been published between 2019 and 2024, focusing on the combination of new technologies that have gradually matured in recent years. Combination treatment regimens include: IPL combined with laser (*n* = 5), IPL combined with corticosteroid hormones (*n* = 5), laser combined with bleomycin (*n* = 1), 5-FU (*n* = 3), botulinum toxin A (*n* = 1), and corticosteroid hormones (*n* = 12) (Table 6).

**Table 6:** Optical combination therapy.

Study	Intervention of the experimental group	Intervention of the controlled group	Outcomes	Indicators of outcome (experimental group)	Indicators of outcome (controlled group)
Daoud 2019 (137)	Combined Intense Pulsed Light (IPL) With Fractional CO_2_-Laser	Fractional CO_2_-Laser	Average decrease in MSS score	Color: 2.21; Matte vs. shiny: 0.67; Contour: 1.87; Distortion: 1.83; Texture: 1.97	Color: 1.2; Matte vs. shiny: 0.87; Contour: 1.5; Distortion: 1.6; Texture: 1.3
Manuskiatti 2021 (58)	Laser + steroid	Laser + petrolatum	Reduction in hypertrophic scars thickness	0.66 ± 0.39	0.69 ± 0.36
Son 2014 (57)	578 nm Copper Bromide Laser Combined with Intralesional Corticosteroid Injection	\	PGA	2.08	\
Tawfic 2020 (71)	Combined: Fractional and Nd YAG	Nd: YAG alone/Fractional laser alone	VSS and POSAS	VSS: 45.63 ± 21.34; PSASA: 44.92 ± 10.13	VSS: 47.34 ± 19.96; PSASA: 46.5 ± 9.22/VSS: 47.34 ± 19.96; PSASA: 46.5 ± 9.22
Wang 2020 (67)	Combined ultrapulse fractional carbon dioxide laser and topical triamcinolone	\	Patient and observer scores	Observer: 19.17 ± 9.09; patient: 15.15 ± 7.51	\
Sabry 2020 (150)	Combined laser and intralesional injection of botulinum toxin A	Intralesional injection of botulinum toxin A	VSS	0.6 ± 0.2	0.7 ± 0.2
Alhamzawi 2021 (68)	Fractional carbon dioxide laser with intralesional 5-Fluorouracil (50 mg/mL)	\	VSS improvement rate	0.65	\
Annabathula 2017 (156)	Fractional carbon dioxide, long pulse Nd: YAG and pulsed dye laser	\	Efficacy	0.7273	\
Meymandi 2014 (69)	Intense pulsed light method along with corticosteroid injection	\	Efficacy	89.1%	\
Stephanides 2011 (162)	Intralesional triamcinolone (10 mg or 40 mg/dL) and pulsed dye laser	\	Efficacy	76%	\
Kim 2015 (164)	Intense pulsed light device and intralesional corticosteroid injection	\	Improvement in MVSS	0.981	\
Kim 2022 (40)	Pulsed dye laser therapy combined with intralesional triamcinolone injection	\	VSS	3.11 ± 1.52	\
Hye 2015 (70)	Copper bromide laser and intralesional triamcinolone injection (2.5 mg/mL or 5 mg/mL)	\	VSS	\	\
Tawaranurak 2022 (42)	Treated with fractional CO_2_ laser + topical triamcinolone	Intralesional TA	Efficacy/Recurrence rate	63.6%/9.1%	72.7%/18.2%
Ramadan 2021 (90)	pulsed Nd: YAG laser and intralesional bleomycin	Pulsed Nd: YAG laser only	Efficacy	1	0.8
Dai 2021 (181)	Combination of ablative fractional carbon dioxide laser and platelet-rich plasma	Ablative fractional carbon dioxide laser	VSS	6.06 ± 1.44	8.16 ± 1.93
Dina 2021 (107)	Combined fractional ablative 2,940 nm Er: YAG laser and topical application of steroid cream	Intralesional corticosteroid injection	VSS	2.07 ± 2.02	2.63 ± 2.09
Alexander 2018 (55)	Fractional CO_2_ laser with intralesional steroid	Intralesional steroid alone	Efficacy	\	\
Sabry 2019 (64)	CO_2_ laser and topically applied 5-FU	CO_2_ laser and topically applied verapamil hydrochloride/ablative fractional CO_2_ laser monotherapy	VSS	5.33 ± 1.6	4.3 ± 1.5/1.89 ± 1.05
Shin 2019 (43)	Combination of non-ablative fractional laser and intralesional triamcinolone injection	Intralesional triamcinolone injection	Recurrence rate	35.3%	38.1%
Tsai 2019 (56)	Combination of 1,064-nm neodymium-doped yttrium aluminum garnet laser and steroid tape	Steroid Tape	Time of total JSS score <3	16.9 months	24.3 months
Tawfik 2019 (190)	Combined 5- fluorouracil and fractional erbium YAG laser	Topical 5-fluorouracil cream	VSS	2.17 ± 1.11	4 ± 1.76
Liu 2023 (44)	Combined pulsed dye laser and triamcinolone acetonide	Triamcinolone acetonide	VSS	4	5
Zhang 2023 (194)	Fractional carbon dioxide laser + 5-aminolevulinic acid photodynamic therapy	\	POSAS	38.58 ± 4.71	44.04 ± 5.07
Jiang 2024 (197)	Intralesional triamcinolone + ND: YAG laser	Intralesional triamcinolone	VSS and VAS	VSS: 9.75 ± 0.43; VAS: 2.5 ± 2.4	\
Park 2024 (199)	Combined continuous wave and repetitive fractionated CO_2_ laser	\	MVSS, POSAS, Recurrence rate, and Overall patient satisfaction	MVSS: 3.5 ± 0.3; POSAS: 1.9 ± 0.3; Recurrence rate: 4.35%; Overall patient satisfaction:4.5 ± 0.5	MVSS: 3.7 ± 0.5; POSAS: 2.7 ± 0.4; Recurrence rate: 12.5%; Overall patient satisfaction:4.4 ± 0.6
Aristides 2024 (201)	Fractional carbon dioxide laser + 5-aminolevulinic acid photodynamic therapy	\	VSS	1.7 ± 2.1	\

Nine single-arm studies have confirmed the preventive and therapeutic effects of laser combined with corticosteroid hormone (67), laser combined with 5-FU (68) and IPL combined hormone (40, 69) on keloid and hypertrophic scar (57). Adjuvant use of the 578 nm CO_2_ laser reduced the telangiectasia side effect of intrafocal corticosteroid injection (57), but the effect was slower and required 3 to 4 courses to reduce the scar VSS score by 50% (70).

The results of 14 controlled studies showed that the treatment effect of laser combined with IPL, laser combined with corticosteroid hormone, laser combined with botulinum toxin A, and laser combined with 5-FU were all better than that of laser or other monotherapy. Notably, the results of a small sample RCT study published in 2020 showed that the combination of the two laser modes (fractional and Nd YAG) in the same course of treatment did not add significant additional benefit and had higher side effects (VSS: 45.63 ± 21.34; PSASA: 44.92 ± 10.13) (71).

### Radiation monotherapy

The radiation therapy regimen was the only regimen in this search in which monotherapy was significantly more than combination therapy. This is mainly because almost all radiation therapy is used as an adjunctive treatment option after surgical resection to aid in the recovery and later management of keloids (Table 7).

**Table 7:** Radiation monotherapy.

Study	Intervention of the experimental group	Intervention of the controlled group	Outcomes	Indicators of outcome (experimental group)	Indicators of outcome (controlled group)
Wen 2021 (140)	Hypofractionated radiotherapy	\	Efficacy	0.848	\
Arnault 2009 (146)	Iridium 192* brachytherapy	\	Recurrence rate	0.236	\
Bijlard 2017 (148)	High-dose-rate brachytherapy	\	Recurrence rate	0.083	\
Lee 2015 (76)	6-MeV electron beam	\	Recurrence rate	0.1891	\
Bischof 2007 (128)	6-MeV electron beam radiotherapy	\	Recurrence rate	0.85	\
Clavere 1997 (152)	Iridium 192 brachytherapy	\	Efficacy	0.63	\
BERMAN 2020 (157)	Superficial Radiation Therapy	\	Efficacy	85.6%	\
Barragan 2022 (159)	Interstitial high-dose-rate brachytherapy	\	Efficacy	71%	\
Jiang 2015 (73)	Interstitial high-dose-rate brachytherapy	\	Efficacy	94%	\
Jiang 2017 (74)	Interstitial high-dose-rate brachytherapy	\	Efficacy	92%	\
Guix 2001 (160)	Interstitial high-dose-rate brachytherapy	\	Recurrence rate	4.7%	\
Hafkamp 2017 (161)	Interstitial high-dose-rate brachytherapy	\	Efficacy/Recurrence rate	76%/24.1%	\
Maemoto 2020 (134)	Electron beam radiation therapy	\	Recurrence rate	68%	\
Ogawa 2002 (165)	Electron beam radiation therapy	\	Recurrence rate	33.33%	\
Song 2014 (170)	Single-fraction radiotherapy	\	Recurrence rate	0%	\
Shen 2015 (172)	Hypofractionated electron-beam radiation	\	Efficacy and Recurrence rate	88.25%/9.59%	\
Ahmad 2017 (174)	Iridium-192 high-dose rate surface mould brachytherapy	\	Efficacy	89.5%	\
Sruthi 2017 (75)	Single-fraction radiation	\	Recurrence rate	16.2%	\
Son 2020 (77)	A single dose of low-energy superficial X-ray radiation	\	Recurrence rate	6.25%	\
VIANI 2009 (178)	Strontium 90 brachytherapy	\	Recurrence rate and efficacy	12.4%/70.6%	\
Hoang 2016 (79)	Interstitial high dose rate brachytherapy	excision alone/external beam radiotherapy	Recurrence rate	0.23	54%/19%
Cicco 2013 (78)	High-dose-rate interstitial brachytherapy	low-dose-rate interstitial brachytherapy	Recurrence rate	38%	30.4%

Of the 22 radiation monotherapy regimens included, 20 were single-arm studies, all of which confirmed the efficacy and safety of radiation therapy in the adjuvant treatment of postoperative keloids and hypertrophic scars. These adjuvant treatment options include: hypofractionated radiotherapy (*n* = 1), Iridium 192* brachytherapy (*n* = 3), high-dose-rate Brachytherapy (*n* = 6), 6-MeV electron beam (*n* = 2), Superficial Radiation Therapy (*n* = 2), electron beam radiation therapy (*n* = 3), single-fraction radiotherapy (*n* = 2), and strontium 90 brachytherapy (*n* = 1).

These studies of monotherapy with radiation have highlighted several key points related to treatment: (1) perioperative high-dose brachytherapy for keloid was effective, with a recurrence rate of only 4.9% (72); (2) Brachytherapy can be used as a remedy after external radiation therapy and other treatment methods have failed (73, 74); (3) Early postoperative radiotherapy is a good choice for keloid treatment (48–72 h) (75, 76); (4) Recent studies have shown that, for example, a single dose of low-energy superficial X-ray radiation (single dose of 50 kV superficial radiation of 8 Gy administered on average 34 days) is sufficient to inhibit keloid recurrence (77).

One of the two controlled studies included in the review supplemented the results of the single-arm study. A 2013 Cicco (78) study found that low-dose-rate interstitial brachytherapy (LDR) and high-dose-rate interstitial brachytherapy (HDR) had similar recurrence rates. However, the HDR treatment regimen provided better symptom relief than the LDR regimen. However, another controlled study contradicted the conclusions of the one-arm study: the results of the Hoang 2016 study (79) showed that keloid recurrence of external beam radiotherapy was significantly later than that of interstitial high dose rate brachytherapy.

### Radiation combination therapy

There are two types of combined treatment of radiotherapy: one is as postoperative adjuvant treatment, and the other is combined with corticosteroid hormones. Radiotherapy regimens used in combination regimens include HDR, Iridium 192 interstitial irradiation, X-ray therapy, single-fraction radiotherapy, and radiofrequency. The greatest benefit of radiation combination therapies is to control the recurrence of keloid and hypertrophic scars. The rate of recurrence control with this protocol as an adjunctive postoperative treatment gradually decreased from 28% in 1981 (combined surgical excision and immediate X-ray therapy) (80) to 0.02% in 2021 (combined surgical excision and adjuvant HDR) (81). The researchers believe that a postoperative sterile environment created by radiotherapy is an important condition for recurrence control (82). Given the significant influence of scar repair on patient satisfaction, recent research has sought to incorporate advancements in plastic surgery into procedures that combine surgical intervention with radiotherapy. For instance, studies have demonstrated that the application of innovative super-tension suture technology to alleviate skin tension can effectively decrease the rate of postoperative recurrence (83). At the same time, the scar treatment effect and tolerance of excision surgery combined with radiotherapy were better than that of cryotherapy combined with corticosteroids (efficacy: 0.818 vs. 0.719) (84). Furthermore, underdeveloped surgical and radiotherapy techniques may significantly influence treatment outcomes. A limited retrospective study conducted on Pakistani patients, published in 2025, revealed that the postoperative recurrence rate following the combination of surgery and radiotherapy was as high as 65% (85) (Table 8).

**Table 8:** Radiation combination therapy.

Study	Intervention of the experimental group	Intervention of the controlled group	outcomes	Indicators of outcome (experimental group)	Indicators of outcome (controlled group)
Manjunath 2021 (81)	Surgical Excision and Adjuvant High-dose Rate Brachytherapy	\	Recurrence rate	0.02	\
Escarmant 1993 (82)	Iridium 192 interstitial irradiation after surgical excision	\	Efficacy	0.8	\
Ogawa 2014 (169)	Surgical excision and radiotherapy	\	Recurrence rate	4.8%	\
Ollstein 1981 (80)	Combined surgical excision and immediate X-ray therapy	\	Recurrence rate	28%	\
Ragoowansi 2002 (171)	Surgical excision and immediate single-fraction radiotherapy	\	Recurrence rate	16%	\
Weshay 2015 (173)	Combination of radiofrequency and intralesional steroids (10 mg/mL)	\	Volume reduction/Recurrence rate	95.42 ± 7.62/10%	\
Emad 2010 (84)	Surgical excision and radiotherapy	Cryotherapy and intralesional steroid	efficacy and tolerability	0.818	0.719
Kaushal 2020 (149)	Combined intralesional radiofrequency and intralesional triamcinolone acetonide	Intralesional triamcinolone acetonide alone	Efficacy	0.9	0.867
Li 2024 (195)	Keloid-cross-flap surgery and radiotherapy	Keloid-cross-flap surgery and compression therapy	Recurrence rate	13.56%	28.81%
Khan 2025 (85)	Surgical excision and radiotherapy	\	Recurrence rate	65%	\

### Antipyrimidine therapy

5-fluorouracil is the first antimetabolic drug synthesized according to certain assumptions and is the most widely used antipyrimidine drug in the clinic. It has good efficacy in digestive tract cancer and other solid tumors, and plays an important role in medical oncology. This treatment mode has fewer cases of single drug use (*n* = 1), and more combined with glucocorticoids (TA) (*n* = 7), which has better, faster efficacy and fewer side effects than glucocorticoids alone (48) (Table 9).

**Table 9:** Antipyrimidine therapy.

Study	Intervention of the experimental group	Intervention of the controlled group	Outcomes	Indicators of outcome (experimental group)	Indicators of outcome (controlled group)
Antipyrimidine monotherapy					
Li 2022 (158)	Excision followed by 5-FU and betamethasone intralesional injections	5-FU and betamethasone intralesional injections/excision followed by radiotherapy	VSS and POAS	0.5516	37.11%/54.11%
Reinholz 2020 (41)	Intralesional 5-fluorouracil (50 mg/mL) in combination with triamcinolone acetonide (40 mg/mL)	\	improvement in POSAS	39%	\
Khalid 2018 (45)	Intralesional 5-FU/TA injections	Radiotherapy	Efficacy	0.7333	0.4333
Erlendsson 2022 (47)	A pneumatic jet injection with 5-fluorouracil and triamcinolone acetonide	5-FU + TA	Improvement in VSS	55%	25%
Sharma 2021 (51)	Intralesional 5-FU and triamcinolone acetonide	Combination of intralesional bleomycin and triamcinolone acetonide	Efficacy	93.33%	93.33%
Srivastava 2018 (48)	Intralesional triamcinolone acetonide (40 mg/mL) and 5-fluorouracil (50 mg/mL)	Intralesional triamcinolone acetonide (40 mg/mL)/intralesional 5-fluorouracil (50 mg/ml)	VSS	0.3 ± 0.47	0.61 ± 0.45/0.2 ± 0.41
Sabry 2019 (64)	CO_2_ laser and topically applied 5-FU	CO_2_ laser and topically applied verapamil hydrochloride/ablative fractional CO_2_ laser monotherapy	VSS	5.33 ± 1.6	4.3 ± 1.5/1.89 ± 1.05
Tawfik 2019 (190)	Combined 5-fluorouracil and fractional erbium YAG laser	Topical 5-fluorouracil cream	VSS	2.17 ± 1.11	4 ± 1.76
Levenberg 2020 (144)	Intralesional 5FU and corticosteroids	\	VSS improvement rate	0.53	\
Monteiro 2022 (155)	Intralesional 5 Fluorouracil (50 mg/mL)	Combination of 5 Fluorouracil (50 mg/mL) with Triamcinolone Acetonide (40 mg/mL)	Efficacy	\	\
Davison 2009 (180)	5-FU + steroid with excision	5FU + steroid without excision/steroid treatment with excision	Reduction area of Keloids	0.92	81%/73%
Antipyrimidine combination therapy					
George 2005 (133)	Intralesional 5-fluorouracil	\	Recurrence rate	0.3	\

There has also been a gradual increase in the number of new joint programs in recent years. Surgical resection combined with 5-FU is an effective treatment for ear keloids (45). The VSS score of laser combined with 5-FU was better than that of laser or 5-FU monotherapy (64, 86). However, the comparative results also showed that patients treated with a combination of bleomycin and triamcinolone within the lesion experienced greater improvements in keloid signs and symptoms (regarding cosmetic problems, mobility limitations, and tenderness) than patients treated with a combination of 5-FU and triamcinolone (51).

### Bleomycin therapy

Bleomycin belongs to the glycopeptide antibiotic family, which has potent antitumor activity against a variety of lymphomas, head and neck cancers, and germ cell tumors. A small number of studies were retrieved, including four monotherapies and two combination therapies (Table 10).

**Table 10:** Bleomycin therapy.

Study	Intervention of the experimental group	Intervention of the controlled group	Outcomes	Indicators of outcome (experimental group)	Indicators of outcome (controlled group)
Bleomycin monotherapy					
Espana 2001 (153)	Bleomycin	\	Efficacy	100	\
Moravej 2022 (87)	Intralesional bleomycin (1.5 mg/mL)	Intralesional triamcinolone (20 mg/mL)	Reduction area of Keloids	0.615	0.655
Saray 2005 (175)	Dermojet injections of bleomycin (1.5 IU/mL)	\	Efficacy	100%	\
Khan 2019 (88)	Intralesional bleomycine	Intralesional triamcinolone	Improvement in POSAS	26 ± 11.91	34 ± 12.28
Bleomycin combination therapy					
Ramadan 2021 (90)	Pulsed Nd: YAG laser and intralesional bleomycin	Pulsed Nd: YAG laser only	Efficacy	1	0.8
Luo 2023 (89)	The combined application of bleomycin and triamcinolone	\	Recurrence rate	6.9–7.9%	\

Bleomycin monotherapy is administered locally and intrafocally, and four studies have confirmed its efficacy. In the comparative study with intralesional triamcinolone, bleomycin had the same effect as TA in reducing scar area (87) and was better than the TA in improving the POSAS score (88). However, its adverse reactions, such as pain, ulceration, and pigmentation, should not be ignored and should be used with caution (87).

Combined administration studies have shown that bleomycin combined with TA can effectively treat keloid and hypertrophic scar, with a recurrence rate of 6.9 to 7.9% (89). Combined intrafocal bleomycin therapy is a promising approach compared to long-pulse Nd-YAG laser monotherapy (efficacy: 100% vs. 80%) (90).

### Verapamil therapy

Verapamil is a calcium channel blocker. It was introduced as a coronary dilator in 1962. In recent years, it has been used to treat hypertension, cardiovascular and cerebrovascular diseases, pulmonary hypertension, and prevent premature birth (91). Intralesional injection was used to treat keloids, and the number of relevant studies was relatively small. We retrieved 2 studies on monotherapy and 4 studies on combination therapy (Table 11).

**Table 11:** Verapamil therapy.

Study	Intervention of the experimental group	Intervention of the controlled group	Outcomes	Indicators of outcome (experimental group)	Indicators of outcome (controlled group)
Verapamil monotherapy					
Ahuja 2013 (92)	Intralesional verapamil hydrochloride (concentration 2.5 mg/mL)	Intralesional triamcinolone acetonide (concentration 40 mg/mL)	VSS	\	\
Abedini 2018 (91)	Intralesional verapamil (2.5 mg/mL)	Intralesional corticosteroids (40 mg/mL)	VSS	11.08 ± 0.94	3.9 ± 1.23
Verapamil combination therapy					
Kant 2018 (93)	Triamcinolone and verapamil	\	Patient, Observer, and POSAS scores	\	\
Copcu 2004 (94)	Combination of Surgery and Intralesional Verapamil Injection	\	Patient satisfaction (1–10)	6.4	\
Dogahe 2023 (52)	Intralesional triamcinolone (40 mg/mL) and verapamil (2.5 mg/mL)	Intralesional triamcinolone alone (40 mg/mL)	VSS	1.5 ± 0.6	4.1 ± 1.9
Khattab 2019 (145)	Intralesional verapamil alone 2.5 mg/mL.	Combination of PDL and intralesional verapamil alone 2.5 mg/ml	VSS and PSS	0.27 ± 0.7	0.13 ± 0.35

The results of monotherapy studies do not support verapamil’s ability to treat keloid and hypertrophic scars alone (VSS: 11.08 ± 0.94 vs. 3.9 ± 1.23) (91), although it is less costly and has fewer side effects than TA (92).

The results of the controlled study showed that the efficacy of verapamil combination therapy was highly recognized (93), and the efficacy of the combination with TA was long-term stable and better than that of TA alone (52). Combined surgery or PDL is also a good choice for clinical treatment, but the number of studies and samples is small, and further research support is needed (94, 95).

### Perioperative adjuvant therapy

Perioperative adjuvant treatment options have been discussed in the above review of various treatment options, and many adjuvant treatment options are considered to be effective in preventing postoperative keloid formation and recurrence. The adjuvant treatment options we searched included: radiotherapy (X-ray therapy, HDR, single-fraction radiotherapy, electron external beam radiation) (*n* = 6), perioperative corticosteroid injection (*n* = 2), verapamil (*n* = 1), and topical mitomycin-C (*n* = 1) (96). Two other controlled studies compared the effectiveness of different combination treatment regimens. Results of a cohort study involving 160 patients with earlobe keloid showed that in the treatment of ear scar, the efficacy of surgery combined with glucocorticoid and electron irradiation group was superior to surgery combined with local injection of glucocorticoid group, equivalent to surgery combined with superficial X-ray group, and superior to surgery group (97) (Table 12).

**Table 12:** Perioperative adjuvant therapy.

Study	Intervention of the experimental group	Intervention of the controlled group	Outcomes	Indicators of outcome (experimental group)	Indicators of outcome (controlled group)
Copcu 2004 (94)	Combination of Surgery and Intralesional Verapamil Injection	\	Patient satisfaction (1–10)	6.4	\
Manjunath 2021 (81)	Surgical Excision and Adjuvant High-dose Rate Brachytherapy	\	Recurrence rate	0.02	\
Aljodah 2021 (167)	Combination of Surgical Excision and Perioperative Corticosteroid Injection (40 mg/mL)	\	Recurrence rate	9.6%	\
Ogawa 2014 (169)	Surgical excision and radiotherapy	\	Recurrence rate	4.8%	\
Ollstein 1981 (80)	Combined surgical excision and immediate X-ray therapy	\	Recurrence rate	28%	\
Ragoowansi 2002 (171)	Surgical Excision and immediate single-fraction radiotherapy	\	Recurrence rate	84%	\
Stewart 2006 (96)	The combination of surgical excision with the application of topical mitomycin-C	\	Efficacy	0.9	\
Wang 2020 (179)	Combined surgical excision and electron external beam radiation	\	Recurrence rate	0.086	\
Emad 2010 (84)	Surgical excision and radiotherapy	Cryotherapy and intralesional steroid	Efficacy and tolerability	0.818	0.719
Hou 2023 (191)	Punch Excision Combined With Intralesional Steroid Injection	Intralesional Steroid Injection alone	VSS and POSAS	\	\
Qiao 2017 (97)	Surgery combined with lucortriticod and electron irradiation group		Efficacy	0.975	52.5%/80%/82.5%

### Intralesional cryosurgery

Intralesional Cryosurgery is a safe and effective new treatment that differs from traditional resection, so we summarized the relevant findings separately. It causes minimal damage to the skin surface by destroying hypertrophic scar tissue (98) (Table 13).

**Table 13:** Intralesional cryosurgery.

Study	Intervention of the experimental group	Intervention of the controlled group	Outcomes	Indicators of outcome (experimental group)	Indicators of outcome (controlled group)
Intralesional cryosurgery monotherapy					
Chopinaud 2014 (98)	Intralesional Cryosurgery	\	Reduction area of Keloids	0.585	\
Bijlard 2018 (101)	Intralesional cryotherapy	Excision with corticosteroid injections or brachytherapy	POSA	\	\
Leeuwen 2014 (100)	Intralesional Cryotherapy	\	Recurrence rate	0.24	\
Abdel-Meguid 2014 (99)	Intralesional cryosurgery	Contact cryosurgery	Efficacy	0.87	0.6
Intralesional cryosurgery combination therapy					
Weshahy 2012 (154)	Combined intralesional cryosurgery and intralesional steroid injection	\	Reduction area of Keloids	0.935	\
Nishi 2022 (104)	Combination of cryotherapy with intralesional corticosteroid	Combination of fractional CO_2_ laser followed by topical corticosteroids	MSS	15.67	12.56
Yosipovitch 2009 (103)	Cryotherapy and steroid injection	Cryotherapy alone/Steroid injection	Thickness, pain, and itch	7.7 ± 4.2 mm	3.1 ± 2.2 mm/−0.25 ± 1.2 mm
Zouboulis 2020 (102)	Combined liquid nitrogen contact cryosurgery with intralesional corticosteroids	liquid nitrogen contact cryosurgery	Efficacy	90%	83.3%
Stromps 2013 (186)	Intralesional cryotherapy combined with postoperative silicone gel sheeting	Intralesional cryotherapy alone	Efficacy	\	\

The results of monotherapy studies suggest that intrafocal freezing is superior to contact freezing in terms of efficacy and safety (99), and can reduce the volume, pain, and pruritus of primary scars (100, 101). However, in the treatment of refractory keloids, the efficacy is not as good as excision surgery combined with radiotherapy (101). Combined therapy studies have shown that the adjuvant effect of hormones is not significant when freezing combined hormones, but the effect is better than monotherapy (102–104).

### Topical therapy

Topical therapy is frequently employed for mild keloid and hypertrophic scar treatment, including facial (105) and postcesarean scars (106). The number of relevant studies searched in this study was limited, mainly monotherapy (*n* = 6), and there was only 1 study of combination therapy (107) (Table 14).

**Table 14:** Topical therapy.

Study	Intervention of the experimental group	Intervention of the controlled group	Outcomes	Indicators of outcome (experimental group)	Indicators of outcome (controlled group)
Topical monotherapy					
Martin-Garcia 2005	Imiquimod 5% Cream	\	Recurrence rate	0.25	\
Berman 2002 (109)	Imiquimod 5% cream	\	Recurrence rate	0%	\
Nor 2016 (110)	Either daily topical clobetasol propionate 0.05% cream under occlusion with a silicone dressing	Monthly intralesional triamcinolone injection	POSAS	26(IQR 18–44)	31(IQR 18–39)
Chernoff 2007 (189)	Dermatix gel	Silicone gel sheeting	elevation of the scars	0.79 mm	1.39 mm
Meseci 2019 (105)	Topical corticosteroid ointment	Untreated	MVSS	2.5(0–11)	4(1–10)
Francesca 2010 (78)	Self-Drying Silicone Gel		POSA	2.48	2.55
Topical combination therapy					
Dina 2021 (107)	Combined fractional ablative 2,940 nm Er: YAG laser and topical application of steroid cream	Intralesional corticosteroid injection	VSS	2.07 ± 2.02	2.63 ± 2.09

Topical therapies studied included 5% imiquimod cream (*n* = 2), 0.05% clobetasol propionate cream (*n* = 1), dermatix gel (*n* = 1), topical corticosteroid ointment (*n* = 1), and facial treatment self-drying silicone gel (*n* = 1). 5% imiquimod cream for keloid can achieve a very low recurrence rate (0–25%) (108, 109). Clobetasol 0.05% propionate cream has fewer adverse reactions than triamcinolone (110). However, the topical corticosteroid ointment group had the same effect as the untreated ointment group [MVSS:2.5 (0–11) vs. 4 (1–10)] (105).

### Other monotherapy

In addition to the above treatment regimens, we also identified seven other studies related to monotherapy. Seven studies included 6 different rare treatment options: continuous tension reduction (111), hyperbaric oxygen therapy (112), intralesional botulinum toxin type A (BTX-A) (113, 114), cold atmospheric-pressure plasma (CAP) (115), interferon alfa-2b and topical (116) and intralesional mitomycin C (117), intralesional vitamin D injection (29), small interfering RNA microneedle patches (118), and intralesional injection of umbilical cord mesenchymal stem cells (30) (Table 15).

**Table 15:** Other monotherapy.

Study	Intervention of the experimental group	Intervention of the controlled group	Outcomes	Indicators of outcome (experimental group)	Indicators of outcome (controlled group)
Chen 2020 (111)	Continuous tension reduction	\	Recurrence rate	7.90%	\
SONG 2018 (112)	hyperbaric oxygen therapy	Surgical excision and radiotherapy	Recurrence rate	5.97%	14.15%
Shaarawy 2014 (113)	Intralesional botulinum toxin type A	Intralesional steroid	efficacy	0.827	0.792
Suwanchinda 2022 (115)	Cold atmospheric-pressure plasma	Untreated	efficacy	0.944	\
Neinaa 2021 (114)	Intralesional injection of botulinum toxin type-A (5 IU/injection point)	Intralesional injection of platelet rich plasma (0.1 mL/injection point)/Intralesional injection of triamcinolone acetonide (20 mg/session)	efficacy	0.85	85%/35%
Berman 1997 (116)	Interferon alfa-2b	excision alone/injection with triamcinolone acetonide	Recurrence rate	0.187	51.2%/58.5%
Seo 2011 (117)	Topical and intralesional mitomycin C (1 mg/mL)	\	VSS	\	\
Pazyar 2024 (196)	Intralesional vitamin D injection	Intralesional triamcinolone injection	VSS	6.91 ± 2.5	4.59 ± 1.4
Harsono 2023 (30)	Intralesional injection of umbilical cord Mesenchymal stem cells	Intralesional injection of triamcinolone acetonide	POSAS	3.5(2–4)	1(0–3)
Lim 2024 (118)	Small interfering RNA microneedle patches	Silicone sheets	Percentage reduction in scar volume	83.78 ± 15.39	74.11 ± 21.6

Continuous tension reduction and hyperbaric oxygen therapy both achieved lower recurrence rates (7.8 and 5.97%) (111, 112). The CAP technique can provide mild to moderate scarring improvement with minimal side effects (115). Compared to traditional TA injections, BTX-A has better cosmetic results in the treatment of keloids (113, 114). Interferon alfa-2b injection at the site of keloidectomy can be an effective postoperative adjuvant therapy (116). Recent studies have investigated the efficacy of innovative therapies for keloids, demonstrating that siRNA microneedle patches can significantly reduce scar volume (118). Additionally, intralesional injection of umbilical cord mesenchymal stem cells has demonstrated potential effectiveness in the treatment of keloids; however, further studies with extended follow-up periods are necessary to assess its cost-effectiveness (30). Limited supporting evidence for these interventions limits their applicability for clinical guidance.

### Triple combination therapy

Considering the propensity of keloid and hypertrophic scars for recurrence and the current absence of a gold standard therapy, the study results demonstrated that multimodal combination therapy achieves long-term remission (119). Therefore, with the advent of multiple treatment schemes, multimodal combined treatment schemes emerged. The pertinent treatments identified in this study can be classified into two categories: triple therapy incorporating surgical resection and intralesional corticosteroid injection (*n* = 5), and triple therapy involving intralesional corticosteroid injection in conjunction with 5-FU (*n* = 5).

There were 4 triple treatments based on surgical resection combined with intralesional corticosteroid injection included in this review, and they were combined with three different physiotherapy methods: ablative CO_2_ laser (*n* = 1) (119), shaving (*n* = 1) (120) and local pressure (*n* = 2) (121, 122), respectively. As an adjunctive treatment after surgery, they all helped patients achieve good efficacy and recurrence control (efficacy: 0.824/0.9167; recurrence rate: 12.5%) (119–122), but the scheme to combine local pressure was seen as too inefficient and a lengthy and time-consuming process for both doctors and patients (122).

The construction concept of the triple therapy regimen, based on intrafocal corticosteroid injection combined with 5-FU, is to reduce the recurrence of small keloids after local injection of triamcinolone and 5-fluorouracil (123). Therefore, the combination regimen of radiotherapy (Strontium-90 brachytherapy) (123) and laser therapy [1,064-nm Nd: YAG laser (124) or PDL (3)] was chosen. Combined radiotherapy reduced the recurrence rate of TA combined with local injection of 5-FU by about 40% (44.4% vs. 85.7%) (123).

## Discussion

This review included 162 articles evaluating therapeutic interventions for keloid and hypertrophic scars. The included studies described and compared multiple monotherapy and combination regimens, including intralesional corticosteroid injection, optical therapies, radiotherapy, 5-FU therapy, bleomycin, verapamil, surgical excision, cryotherapy, topical treatments, other monotherapies, and triple-modality regimens. This qualitative systematic review synthesizes nearly all available clinical treatment studies for keloids and hypertrophic scars since 1981. Unfortunately, due to the lack of a gold standard in this area, the included studies are highly personalized, and quantitative comparison of the efficacy of various treatment methods is not supported at this time. Even so, through a systematic review, we can still find that, with the advancement of technology, the treatment options for this disease are gradually expanding, but various combination treatment options still center on local corticosteroid injection, excision surgery, laser, and radiation therapy. At present, the main difficulty in treating keloids and hypertrophic scars is controlling recurrence. Studies have shown that multimodal combined therapy is more effective than monotherapy in solving this problem.

The mechanisms of physical interventions such as phototherapy and surgical procedures for keloid and hypertrophic scar treatment are relatively straightforward. Intralesional corticosteroids (e.g., TA) exert anti-inflammatory and antifibrotic effects by suppressing fibroblast proliferation and collagen synthesis (31, 89). Antimetabolites such as 5-fluorouracil inhibit fibroblast growth through interference with DNA synthesis (41, 47), while bleomycin induces DNA strand breaks, arresting cell division (89, 125–127). Verapamil, a calcium-channel blocker, may modulate extracellular matrix remodeling by influencing collagenase activity (37). Physical modalities, including laser/IPL (6, 13, 14), radiotherapy (78, 128), cryotherapy (6, 13), and surgical excision (11), act via direct tissue ablation, inhibition of proliferating cells, or physical removal of scar tissue. The observed superiority of combination regimens likely stems from complementary mechanisms that simultaneously target different pathways in scar pathogenesis. Therefore, this discussion elaborates on disease recurrence mechanisms and the pharmacological principles of major therapeutic agents (intralesional TA, 5-FU, bleomycin, and verapamil). Post-treatment recurrence rates represent a primary impetus for investigating intralesional TA as an alternative therapeutic strategy. Long-term follow-up investigations indicate that recurrence rates associated with TA monotherapy or combination therapy range from 5 to 7% over 2–5 years (31, 89).

Keloid and hypertrophic scars, as pathological scar tissue secondary to skin trauma or spontaneous overgrowth, demonstrate therapeutic resistance and elevated post-treatment recurrence rates similar to neoplastic diseases (129). Clinical studies have shown that mono-mode treatment and inadequate treatment (not according to the course of treatment or insufficient dosage) are high-risk factors for keloid recurrence (89). The researchers found that, during keloid treatment, the drug has a temporary effect and is difficult to penetrate the scar. The recurrence rate of keloid with a larger scar area and thicker hyperplasia is higher than that of a smaller scar. This may be caused by inadequate administration or insufficient coverage of medications (89). The recurrence rate of keloid with a larger scar area and thicker hyperplasia is higher than that of a smaller scar. This may be caused by inadequate administration of the drug in some areas or insufficient coverage of the drug. The high recurrence rate after treatment is also the main reason the search for alternatives to intralesional TA has never stopped, despite its widespread use. Furthermore, researchers speculate that the above dilemma may be caused by high heterogeneity among cases, and differences in race, local tissue tension, infections, and even different endocrine environments may affect the traits and prognosis of the disease (130–132). Thus, several studies have provided further insight into the pathogenesis and possible biomarkers of the disease. Recently, S1P-induced signal transduction has been associated with increased collagen synthesis in keloid tissues via S1PR-mediated signaling pathways (17). In terms of genetic and epigenetic aspects of keloid, studies have found that sHLA-E (18), PAI-1, and VDR (19) can effectively be used as diagnostic markers to assess the risk of keloid formation, among which sHLA-E has the potential to be a prognostic marker for clinical outcomes of localized treatment (18). Molecular mechanism studies have shown that EGFR/miR-370-3p/ LCC-GLB1L-1, ITGB5/miR-204/ LCC-Casp9-3 (23), and Hsa_circ_0043688 (25) may be involved in the pathogenesis of keloid. By analyzing the differences in gene expression between keloid and normal dermal fibroblasts through gene profiling, we found that differential expression of the HOX gene (24) and downregulation of Smad7 via MeCP2 may play a role in keloid development (27). Recent studies have also found that five central genes, CHI3L1, IL1RN, MMP7, TNFAIP3, and TNFAIP6, are associated with keloid recurrence (26).

Keloid and hypertrophic scar are tumor-like diseases, and the attempt of antitumor drugs to treat these diseases is also a topic of interest to researchers. As an intralesional TA combination regimen, bleomycin has been found to reduce keloid volume and inhibit keloid recurrence when used in combination with bleomycin (89). Bleomycin can bind to the DNA of the cell, break the DNA chain, and block cell division and proliferation. The M-stage cells are the most sensitive to bleomycin (125, 126). Bleomycin’s role in disrupting cell division in fibroblasts has also been demonstrated *in vitro* (127).

The pyrimidine analogue 5-FU has also been selected as a TA combination therapy, and its therapeutic effect on keloid and hypertrophic scar is also derived from the inhibition of fibroblast proliferation (41). Studies have shown that the combination of 5-fluorouracil and corticosteroids has a faster clinical response and fewer side effects than single-drug therapy (47). Meanwhile, long-term follow-up results indicated that the two-year recurrence rate of intralesional 5-FU monotherapy could be reduced to 3% (133). At present, the discussion on the administration ratio and regimen of combination therapy is the focus of relevant studies, and the regimen with a 5-FU ratio of TA equal to 3:1 is the conclusion that researchers generally tend to reach (41, 47).

Finally, verapamil is a calcium channel blocker that promotes collagen breakdown by reducing the expression of MMP-9 and vascular endothelial growth factor in keloid tissues, preventing the synthesis and secretion of collagen and fibronectin (extracellular matrix molecules) (37). However, the research results we retrieved only support the application of verapamil as an adjuvant therapy, and the efficacy of a single drug in the treatment of scar remains doubtful (91, 95). Furthermore, the clinical research results of some new therapies, such as botulinum toxin type A, biologics (dupilumab), and interferon (interferon alfa-2b), are also very encouraging. Due to the small number of studies and sample sizes, it is still unable to shake the status of mainstream treatment. However, until the gold standard of care for keloids and hypertrophic scars is established, designing stability and cost-effective protocols to compare traditional treatment (relative to corticosteroid therapy and excision) and new therapies remains the focus of head-to-head trials.

It is imperative to note that previous studies have reported concerning long-term recurrence rates associated with physics-based radiotherapy, surgical resection, and laser therapies. Six studies included in the analysis reported recurrence rates ranging from 2 to 10 years for both combined modalities of radiotherapy and surgery or as standalone treatments. Notably, the recurrence rate following carbon dioxide laser ablation alone may reach as high as 100% within a 2-year period (61). Furthermore, the implementation of continuous tension reduction has been shown to decrease the recurrence rate by 92.1% (111). The recurrence rate for radiotherapy was documented at 38% within 2 years (78), escalating to between 60 and 80% over a 5 to 10-year timeframe (128, 134). Previous studies have indicated that the declining trend in local control rates of lesions following physical interventions is associated with the presence of multiple lesions and the delineation of scar boundaries. This phenomenon parallels the relationship observed between drug dosage and therapeutic efficacy in localized pharmacological treatments. Consequently, future research should undertake a comprehensive investigation into the impact of the local treatment area on both the therapeutic outcomes and recurrence rates of keloids and hypertrophic scars.

As for combination therapy, the ‘S2k guidelines for the therapy of pathological scars (hypertrophic scars and keloids) – Update 2020’ provided a set of treatment algorithms based on scar size, treatment difficulty, and erythema persistence. Non-active flat keloid scars with small areas can be treated with TA and/or cryosurgery. A combination of TA and cryosurgery and 5FU or surgery with follow-up treatment is recommended for further refractory keloid. On this basis, the pulsed dye laser should be considered for persistent erythema. For narrow-base refractory keloids, excision and mandatory follow-up treatment (compression /TA/ cryosurgery/radiation, plus appropriate conservative local treatment) or local cryosurgery are recommended. Local treatment was currently the main treatment for the disseminated and confluent refractory keloids (135). Although this guideline puts forward a detailed treatment algorithm, a number of reviewed studies, including the guideline itself, believed that the preferred treatment method for keloid scars cannot be standardized (136). The need for keloid treatment and the issue of personalized therapeutic goals limited the design and development of standardized clinical trials to a certain extent. At present, there are few high-quality, large-sample RCTs evaluating different treatment methods of keloids. Most evidence supporting keloid therapy consists of retrospective cohorts, prospective cohorts, and systematic reviews. Designing reliable trials to investigate keloid treatment modalities requires a standardized set of experimental methods. Such an approach requires that studies accurately identify keloid populations, design consistent indicators for evaluating treatment quality, and apply validated scar assessment tools. Therefore, the accumulation of treatment plans based on high-quality studies with reproducible outcomes is the difficulty and direction of progress in the field of keloid diagnosis and treatment.

A critical appraisal reveals marked heterogeneity across studies in design, patient populations, interventions, outcome measures, and follow-up duration, which limits pooled comparison and weakens causal inference. Evidence strength is highest for intralesional corticosteroids, radiotherapy as a surgical adjuvant, and several laser modalities because multiple controlled studies and long-term cohorts support their efficacy; however, many trials report variable endpoints and incomplete reporting. Combination regimens consistently outperform monotherapies, yet optimal combinations, dosing ratios, and sequencing remain undefined. Future trials must standardize diagnostic criteria, adopt validated scar scales (e.g., VSS, POSAS), ensure adequate follow-up, and prioritize randomized, adequately powered designs to reduce heterogeneity and strengthen evidence.

Optimal multimodal therapy may vary by anatomical site. For example, earlobe keloids, given their small size and low tension, often respond well to surgical excision combined with adjuvant intralesional steroids or radiation. Conversely, keloids on the chest or shoulders may require tension-relieving techniques in addition to antiproliferative therapies. Regarding specific laser and steroid parameters, current evidence does not define a single optimal protocol; studies report wide variations in laser settings (e.g., fluence, density) and triamcinolone concentrations (10–40 mg/mL) (42, 58, 70). Future studies should aim to standardize these parameters to establish more precise treatment guidelines.

## Limitation

This systematic review integrates a substantial portion of the existing literature and employs the MINOR and ROB2 checklist criteria for quality assessment; however, several potential limitations warrant acknowledgment. First, the study’s inclusion criteria were restricted to English-language publications, which may introduce bias into the comprehensive evaluation results, particularly given that the incidence of keloids and hypertrophic scars is influenced by racial factors. Secondly, conference abstracts, which frequently appear in contemporary research on treatment approaches, were excluded, potentially resulting in the omission of some of the latest therapeutic modalities. Thirdly, there is a notable lack of discussion of the potential short- and long-term side effects of treating keloids and hypertrophic scars. This oversight may be attributed to the localized nature of the interventions employed. Nevertheless, given the possible adverse effects of long-term treatments, such as corticosteroids, it is essential that future research more thoroughly examine the potential safety risks involved. Moreover, the review has limited analytical depth and lacks a quantitative synthesis of the findings, making it challenging to draw definitive conclusions regarding the effectiveness of various treatment strategies. Furthermore, in the absence of a gold standard for the treatment of keloids and hypertrophic scars, there is considerable variability in treatment modalities and outcome variables across different studies, which hinders the quantitative synthesis of analytical results. This study, therefore, provides only a qualitative summary of the evidence. Although a range of treatment methods has been reviewed, a uniform conclusion regarding their efficacy remains elusive. Consequently, the findings presented in this study should be interpreted with caution.

## Conclusion

This systematic review synthesizes existing literature on treatment modalities and outcomes for keloid and hypertrophic scars. Following a qualitative analysis of 162 studies, we conclude that intralesional glucocorticoid injection, excisional surgery, and laser therapy represent the three most frequently employed treatment strategies. Multimodal combination therapy is often regarded as superior to monotherapy for mitigating recurrence rates. Proper administration and adherence to treatment protocols are crucial for ensuring favorable prognoses. Currently, standardized management guidelines for keloid treatment remain absent. The development of standardized trial protocols for keloid treatment, informed by quantitative evidence-based medical investigation, is essential for accumulating robust evidence in future studies.

## Data Availability

The original contributions presented in the study are included in the article/Supplementary material, further inquiries can be directed to the corresponding author/s.
