# Supplementary material for: Therapeutic methods and effect on keloid and hypertrophic scars: a systematic review
Source: Front Med (Lausanne). 2026 Mar 11;13:1702697. doi: 10.3389/fmed.2026.1702697 (PMC13013025; doi:10.3389/fmed.2026.1702697)
Supplement: Supplementary file 1 [file Table_1.docx]

**Search strategy of PubMed**

| NO. | Search Details | Results |
| --- | --- | --- |
| #7 | #1 and (#2 or #3 or #4 or #5) Filters: Humans | 3,512 |
| #6 | #1 and (#2 or #3 or #4 or #5) | 3,578 |
| #5 | ((((((((((((((((((((((((((((((((((((((((((((((((((((((((((((((((((((((((((((((((((((((((((((((((((((((((((((((((((((((((((((((((((((((((((((((((((((((((((((((((((((((((((((((((((((((((((((((((((((((((((((((((((((((((((((((((((((((((((((Occlusive Dressing) OR (Occlusive Bandage)) OR (Occlusive Bandages)) OR (Spray-On Dressing)) OR (Spray-On Dressings)) OR (occlusive dressings)) OR (Compressive therapy)) OR (Intralesional steroids)) OR (Steroid)) OR (Catatoxic Steroids)) OR (cyclosteroids)) OR (steroid compound)) OR (steroid derivative)) OR (steroidal compound)) OR (steroids)) OR (S 26308)) OR (R 837)) OR (Zyclara)) OR (Aldara)) OR (Mitomycin C)) OR (Mitocin C)) OR (NSC 26980)) OR (Ametycine)) OR (Mutamycin)) OR (ameticine)) OR (ametycin)) OR (datisan)) OR (jelmyto)) OR (metomit)) OR (mitocyn c)) OR (mitocyna)) OR (mitomicina-c)) OR (mitomycin-c kyowa)) OR (mitomycine)) OR (mitomycine c)) OR (mitosol)) OR (mitozytrex)) OR (mixandex)) OR (mmc)) OR (mytocine)) OR (mytomicin c)) OR (mytomycin c)) OR (mytozytrex)) OR (vetio)) OR (mitomycin)) OR (5-FU)) OR (5 Fluorouracil)) OR (Fluoruracil)) OR (5 FU Lederle)) OR (5 FU Medac)) OR (5 HU Hexal)) OR (Adrucil)) OR (Carac)) OR (Efudix)) OR (Fluoro Uracile ICN)) OR (Efudex)) OR (Fluoroplex)) OR (Flurodex)) OR (Fluorouracil Mononitrate)) OR (Fluorouracil Monopotassium Salt)) OR (Fluorouracil Monosodium Salt)) OR (Fluorouracil Potassium Salt)) OR (Fluorouracil GRY)) OR (Fluorouracile Dakota)) OR (Fluorouracilo Ferrer Far)) OR (Fluracedyl)) OR (Haemato FU)) OR (Neofluor)) OR (Onkofluor)) OR (Ribofluor)) OR (5 Fluorouracil Biosyn)) OR (5 fluoruracil)) OR (accusite)) OR (actino-hermal)) OR (agicil)) OR (cinkef u)) OR (effluderm)) OR (efurix)) OR (eurofluor)) OR (fivoflu)) OR (fluoro uracil)) OR (fluoroblastin)) OR (fluorouracil 5)) OR (fluorouracil sodium)) OR (fluorouracile)) OR (fluorouracilo)) OR (fluouracil)) OR (fluoxan)) OR (flurablastin)) OR (fluracil)) OR (fluracilium)) OR (fluril)) OR (fluro uracil)) OR (fluroblastin)) OR (fluroblastine)) OR (ifacil)) OR (nsc 18913)) OR (nsc 19893)) OR (oncofu)) OR (tolak)) OR (uflahex)) OR (uraciflor)) OR (utoral)) OR (fluorouracil)) OR (Interferon)) OR (cl 884)) OR (endogenous interferon)) OR (exogenic interferon)) OR (ifn)) OR (interferon type i)) OR (interferone)) OR (interferonogen)) OR (interferons)) OR (interferron)) OR (Bleomycins)) OR (BLEO cell)) OR (Bleolem)) OR (Bléomycine Bellon)) OR (Bleomycin Sulfate)) OR (Bleomycinum Mack)) OR (Blenoxane)) OR (Blanoxan)) OR (Bleomicina)) OR (bileco)) OR (bl 19125)) OR (blenamax)) OR (bleo)) OR (bleocin)) OR (bleocina)) OR (bleocris)) OR (bleomycin analog)) OR (bleomycin derivative)) OR (bleomycin sulphate)) OR (bleomycine)) OR (bleomycinum)) OR (blexit)) OR (blocamicina)) OR (nsc 125066)) OR (bleomycin)) OR (Operative Procedures)) OR (Operative Procedure)) OR (Operative Surgical Procedures)) OR (Surgical Procedures)) OR (Surgical Procedure)) OR (Operative Surgical Procedure)) OR (Ghost Surgery)) OR (operation)) OR (operation care)) OR (operative intervention)) OR (operative repair)) OR (operative restoration)) OR (operative surgery)) OR (operative treatment)) OR (research surgery)) OR (resection)) OR (surgical care)) OR (surgical correction)) OR (surgical diagnosis)) OR (surgical diagnostic techniques)) OR (surgical exposure)) OR (surgical intervention)) OR (surgical management)) OR (surgical operation)) OR (surgical practice)) OR (surgical repair)) OR (surgical research)) OR (surgical restoration)) OR (surgical service)) OR (surgical speciality)) OR (surgical specialties)) OR (surgical specialty)) OR (surgical therapy)) OR (surgical treatment)) OR (surgery)) OR (Cryotherapies)) OR (Cold Therapy)) OR (Cold Therapies)) OR (bath, cold)) OR (cold bath)) OR (cryogenic therapy)) OR (cryothermy)) OR (cryotreatment)) OR (cryotherapy)) OR (Radiotherapies)) OR (Radiation Therapy)) OR (Radiation Therapies)) OR (Radiation Treatment)) OR (Radiation Treatments)) OR (Targeted Radiotherapies)) OR (Targeted Radiotherapy)) OR (Targeted Radiation Therapy)) OR (Targeted Radiation Therapies)) OR (bioradiant therapy)) OR (bucky irradiation)) OR (bucky radiation)) OR (bucky radiotherapy)) OR (bucky ray)) OR (bucky ray radiation)) OR (bucky therapy)) OR (fractionated radiotherapy)) OR (hemibody irradiation)) OR (hypophysis irradiation)) OR (hypophysis radiation)) OR (irradiation therapy)) OR (irradiation treatment)) OR (lymphatic irradiation)) OR (pituitary irradiation)) OR (radiation beam centration)) OR (radiation repair)) OR (radio therapy)) OR (radio treatment)) OR (radiohypophysectomy)) OR (radiotreatment)) OR (roentgen therapy)) OR (roentgen treatment)) OR (rontgen therapy)) OR (therapeutic radiology)) OR (x radiotherapy)) OR (x ray therapy)) OR (x ray treatment)) OR (radiotherapy)) OR (Dye Laser)) OR (Tunable Dye Lasers)) OR (Tunable Dye Laser)) OR (Dye Lasers)) OR (Pulsed Dye Lasers)) OR (Pulsed Dye Laser)) OR (dye laser device)) OR (tunable dye laser device)) OR (Ablative laser)) OR (Laser-assisted drug delivery)) OR (LADD)) OR (Platelet Rich Plasma)) OR (platelet-rich plasma)) OR (thrombocyte rich plasma)) OR (stem cell therapy)) OR (gene therapy) | 19,334,854 |
| #4 | ((((((((((("Occlusive Dressings"[Mesh]) OR "Imiquimod"[Mesh]) OR "Steroids"[Mesh]) OR "Mitomycin"[Mesh]) OR "Fluorouracil"[Mesh]) OR "Interferons"[Mesh]) OR "Bleomycin"[Mesh]) OR "Surgical Procedures, Operative"[Mesh]) OR "Cryotherapy"[Mesh]) OR "Radiotherapy"[Mesh]) OR "Lasers, Dye"[Mesh]) OR "Platelet-Rich Plasma"[Mesh] | 4,927,232 |
| #3 | ((((((((((((((((((((((((CD Antigens) OR (CD Antigen)) OR (Cluster of Differentiation Antigens)) OR (Cluster of Differentiation Marker)) OR (Differentiation Marker Cluster)) OR (Cluster of Differentiation Markers)) OR (Cluster of Differentiation Antigen)) OR (Differentiation Antigen Cluster)) OR (Leukocyte Differentiation Antigens, Human)) OR (leucocyte antigen)) OR (leukocyte antigen)) OR (MicroRNA)) OR (miRNAs)) OR (Micro RNA)) OR (miRNA)) OR (Primary MicroRNA)) OR (Primary miRNA)) OR (pri-miRNA)) OR (pri miRNA)) OR (stRNA)) OR (Small Temporal RNA)) OR (pre-miRNA)) OR (pre miRNA)) OR (microRNAs)) OR (KASS) | 702,539 |
| #2 | ("Antigens, CD"[Mesh]) OR "MicroRNAs"[Mesh] | 235,818 |
| #1 | "Keloid"[Mesh] | 4,653 |

**Search strategy of EMBASE**

| No. | Query | Results |
| --- | --- | --- |
| #19 | #18 AND 'human'/de | 5771 |
| #18 | #1 AND (#2 OR #3 OR #4 OR #5 OR #6 OR #7 OR #8 OR #9 OR #10 OR #11 OR #12 OR #13 OR #14 OR #15 OR #16 OR #17) | 6363 |
| #17 | 'occlusive dressing':ti,ab,kw OR 'occlusive bandage':ti,ab,kw OR 'occlusive bandages':ti,ab,kw OR 'spray-on dressing':ti,ab,kw OR 'spray-on dressings':ti,ab,kw OR 'occlusive dressings':ti,ab,kw OR 'compressive therapy':ti,ab,kw OR 'intralesional steroids':ti,ab,kw OR 'steroid':ti,ab,kw OR 'catatoxic steroids':ti,ab,kw OR 'cyclosteroids':ti,ab,kw OR 'steroid compound':ti,ab,kw OR 'steroid derivative':ti,ab,kw OR 'steroidal compound':ti,ab,kw OR 'steroids':ti,ab,kw OR 's 26308':ti,ab,kw OR 'r 837':ti,ab,kw OR 'zyclara':ti,ab,kw OR 'aldara':ti,ab,kw OR 'mitomycin c':ti,ab,kw OR 'mitocin c':ti,ab,kw OR 'nsc 26980':ti,ab,kw OR 'ametycine':ti,ab,kw OR 'mutamycin':ti,ab,kw OR 'ameticine':ti,ab,kw OR 'ametycin':ti,ab,kw OR 'datisan':ti,ab,kw OR 'jelmyto':ti,ab,kw OR 'metomit':ti,ab,kw OR 'mitocyn c':ti,ab,kw OR 'mitocyna':ti,ab,kw OR 'mitomicina-c':ti,ab,kw OR 'mitomycin-c kyowa':ti,ab,kw OR 'mitomycine':ti,ab,kw OR 'mitomycine c':ti,ab,kw OR 'mitosol':ti,ab,kw OR 'mitozytrex':ti,ab,kw OR 'mixandex':ti,ab,kw OR 'mmc':ti,ab,kw OR 'mytocine':ti,ab,kw OR 'mytomicin c':ti,ab,kw OR 'mytomycin c':ti,ab,kw OR 'mytozytrex':ti,ab,kw OR 'vetio':ti,ab,kw OR 'mitomycin':ti,ab,kw OR '5-fu':ti,ab,kw OR '5 fluorouracil':ti,ab,kw OR 'fluoruracil':ti,ab,kw OR '5 fu lederle':ti,ab,kw OR '5 fu medac':ti,ab,kw OR '5 hu hexal':ti,ab,kw OR 'adrucil':ti,ab,kw OR 'carac':ti,ab,kw OR 'efudix':ti,ab,kw OR 'fluoro uracile icn':ti,ab,kw OR 'efudex':ti,ab,kw OR 'fluoroplex':ti,ab,kw OR 'flurodex':ti,ab,kw OR 'fluorouracil mononitrate':ti,ab,kw OR 'fluorouracil monopotassium salt':ti,ab,kw OR 'fluorouracil monosodium salt':ti,ab,kw OR 'fluorouracil potassium salt':ti,ab,kw OR 'fluorouracil gry':ti,ab,kw OR 'fluorouracile dakota':ti,ab,kw OR 'fluorouracilo ferrer far':ti,ab,kw OR 'fluracedyl':ti,ab,kw OR 'haemato fu':ti,ab,kw OR 'neofluor':ti,ab,kw OR 'onkofluor':ti,ab,kw OR 'ribofluor':ti,ab,kw OR '5 fluorouracil biosyn':ti,ab,kw OR '5 fluoruracil':ti,ab,kw OR 'accusite':ti,ab,kw OR 'actino-hermal':ti,ab,kw OR 'agicil':ti,ab,kw OR 'cinkef u':ti,ab,kw OR 'effluderm':ti,ab,kw OR 'efurix':ti,ab,kw OR 'eurofluor':ti,ab,kw OR 'fivoflu':ti,ab,kw OR 'fluoro uracil':ti,ab,kw OR 'fluoroblastin':ti,ab,kw OR 'fluorouracil 5':ti,ab,kw OR 'fluorouracil sodium':ti,ab,kw OR 'fluorouracile':ti,ab,kw OR 'fluorouracilo':ti,ab,kw OR 'fluouracil':ti,ab,kw OR 'fluoxan':ti,ab,kw OR 'flurablastin':ti,ab,kw OR 'fluracil':ti,ab,kw OR 'fluracilium':ti,ab,kw OR 'fluril':ti,ab,kw OR 'fluro uracil':ti,ab,kw OR 'fluroblastin':ti,ab,kw OR 'fluroblastine':ti,ab,kw OR 'ifacil':ti,ab,kw OR 'nsc 18913':ti,ab,kw OR 'nsc 19893':ti,ab,kw OR 'oncofu':ti,ab,kw OR 'tolak':ti,ab,kw OR 'uflahex':ti,ab,kw OR 'uraciflor':ti,ab,kw OR 'utoral':ti,ab,kw OR 'fluorouracil':ti,ab,kw OR 'interferon':ti,ab,kw OR 'cl 884':ti,ab,kw OR 'endogenous interferon':ti,ab,kw OR 'exogenic interferon':ti,ab,kw OR 'ifn':ti,ab,kw OR 'interferon type i':ti,ab,kw OR 'interferone':ti,ab,kw OR 'interferonogen':ti,ab,kw OR 'interferons':ti,ab,kw OR 'interferron':ti,ab,kw OR 'bleomycins':ti,ab,kw OR 'bleo cell':ti,ab,kw OR 'bleolem':ti,ab,kw OR 'bl茅omycine bellon':ti,ab,kw OR 'bleomycin sulfate':ti,ab,kw OR 'bleomycinum mack':ti,ab,kw OR 'blenoxane':ti,ab,kw OR 'blanoxan':ti,ab,kw OR 'bleomicina':ti,ab,kw OR 'bileco':ti,ab,kw OR 'bl 19125':ti,ab,kw OR 'blenamax':ti,ab,kw OR 'bleo':ti,ab,kw OR 'bleocin':ti,ab,kw OR 'bleocina':ti,ab,kw OR 'bleocris':ti,ab,kw OR 'bleomycin analog':ti,ab,kw OR 'bleomycin derivative':ti,ab,kw OR 'bleomycin sulphate':ti,ab,kw OR 'bleomycine':ti,ab,kw OR 'bleomycinum':ti,ab,kw OR 'blexit':ti,ab,kw OR 'blocamicina':ti,ab,kw OR 'nsc 125066':ti,ab,kw OR 'bleomycin':ti,ab,kw OR 'operative procedures':ti,ab,kw OR 'operative procedure':ti,ab,kw OR 'operative surgical procedures':ti,ab,kw OR 'surgical procedures':ti,ab,kw OR 'surgical procedure':ti,ab,kw OR 'operative surgical procedure':ti,ab,kw OR 'ghost surgery':ti,ab,kw OR 'operation':ti,ab,kw OR 'operation care':ti,ab,kw OR 'operative intervention':ti,ab,kw OR 'operative repair':ti,ab,kw OR 'operative restoration':ti,ab,kw OR 'operative surgery':ti,ab,kw OR 'operative treatment':ti,ab,kw OR 'research surgery':ti,ab,kw OR 'resection':ti,ab,kw OR 'surgical care':ti,ab,kw OR 'surgical correction':ti,ab,kw OR 'surgical diagnosis':ti,ab,kw OR 'surgical diagnostic techniques':ti,ab,kw OR 'surgical exposure':ti,ab,kw OR 'surgical intervention':ti,ab,kw OR 'surgical management':ti,ab,kw OR 'surgical operation':ti,ab,kw OR 'surgical practice':ti,ab,kw OR 'surgical repair':ti,ab,kw OR 'surgical research':ti,ab,kw OR 'surgical restoration':ti,ab,kw OR 'surgical service':ti,ab,kw OR 'surgical speciality':ti,ab,kw OR 'surgical specialties':ti,ab,kw OR 'surgical specialty':ti,ab,kw OR 'surgical therapy':ti,ab,kw OR 'surgical treatment':ti,ab,kw OR 'surgery':ti,ab,kw OR 'cryotherapies':ti,ab,kw OR 'cold therapy':ti,ab,kw OR 'cold therapies':ti,ab,kw OR 'bath, cold':ti,ab,kw OR 'cold bath':ti,ab,kw OR 'cryogenic therapy':ti,ab,kw OR 'cryothermy':ti,ab,kw OR 'cryotreatment':ti,ab,kw OR 'cryotherapy':ti,ab,kw OR 'radiotherapies':ti,ab,kw OR 'radiation therapy':ti,ab,kw OR 'radiation therapies':ti,ab,kw OR 'radiation treatment':ti,ab,kw OR 'radiation treatments':ti,ab,kw OR 'targeted radiotherapies':ti,ab,kw OR 'targeted radiotherapy':ti,ab,kw OR 'targeted radiation therapy':ti,ab,kw OR 'targeted radiation therapies':ti,ab,kw OR 'bioradiant therapy':ti,ab,kw OR 'bucky irradiation':ti,ab,kw OR 'bucky radiation':ti,ab,kw OR 'bucky radiotherapy':ti,ab,kw OR 'bucky ray':ti,ab,kw OR 'bucky ray radiation':ti,ab,kw OR 'bucky therapy':ti,ab,kw OR 'fractionated radiotherapy':ti,ab,kw OR 'hemibody irradiation':ti,ab,kw OR 'hypophysis irradiation':ti,ab,kw OR 'hypophysis radiation':ti,ab,kw OR 'irradiation therapy':ti,ab,kw OR 'irradiation treatment':ti,ab,kw OR 'lymphatic irradiation':ti,ab,kw OR 'pituitary irradiation':ti,ab,kw OR 'radiation beam centration':ti,ab,kw OR 'radiation repair':ti,ab,kw OR 'radio therapy':ti,ab,kw OR 'radio treatment':ti,ab,kw OR 'radiohypophysectomy':ti,ab,kw OR 'radiotreatment':ti,ab,kw OR 'roentgen therapy':ti,ab,kw OR 'roentgen treatment':ti,ab,kw OR 'rontgen therapy':ti,ab,kw OR 'therapeutic radiology':ti,ab,kw OR 'x radiotherapy':ti,ab,kw OR 'x ray therapy':ti,ab,kw OR 'x ray treatment':ti,ab,kw OR 'radiotherapy':ti,ab,kw OR 'dye laser':ti,ab,kw OR 'tunable dye lasers':ti,ab,kw OR 'tunable dye laser':ti,ab,kw OR 'dye lasers':ti,ab,kw OR 'pulsed dye lasers':ti,ab,kw OR 'pulsed dye laser':ti,ab,kw OR 'dye laser device':ti,ab,kw OR 'tunable dye laser device':ti,ab,kw OR 'ablative laser':ti,ab,kw OR 'laser-assisted drug delivery':ti,ab,kw OR 'ladd':ti,ab,kw OR 'platelet rich plasma':ti,ab,kw OR 'platelet-rich plasma':ti,ab,kw OR 'thrombocyte rich plasma':ti,ab,kw OR ' stem cell therapy':ti,ab,kw or ' gene therapy ':ti,ab,kw | 4435981 |
| #16 | 'thrombocyte rich plasma'/exp | 23051 |
| #15 | 'dye laser'/exp | 4602 |
| #14 | 'radiotherapy'/exp | 760044 |
| #13 | 'cryotherapy'/exp | 66974 |
| #12 | 'surgery'/exp | 6738468 |
| #11 | 'bleomycin'/exp | 58500 |
| #10 | 'interferon'/exp | 768389 |
| #9 | 'fluorouracil'/exp | 171450 |
| #8 | 'mitomycin'/exp | 52458 |
| #7 | 'steroid'/exp | 2100517 |
| #6 | 'imiquimod'/exp | 12539 |
| #5 | 'occlusive dressing'/exp | 1479 |
| #4 | 'cd antigens':ti,ab,kw OR 'cd antigen':ti,ab,kw OR 'cluster of differentiation antigens':ti,ab,kw OR 'cluster of differentiation marker':ti,ab,kw OR 'differentiation marker cluster':ti,ab,kw OR 'cluster of differentiation markers':ti,ab,kw OR 'cluster of differentiation antigen':ti,ab,kw OR 'differentiation antigen cluster':ti,ab,kw OR 'leukocyte differentiation antigens, human':ti,ab,kw OR 'leucocyte antigen':ti,ab,kw OR 'leukocyte antigen':ti,ab,kw OR 'microrna':ti,ab,kw OR 'mirnas':ti,ab,kw OR 'micro rna':ti,ab,kw OR 'mirna':ti,ab,kw OR 'primary microrna':ti,ab,kw OR 'primary mirna':ti,ab,kw OR 'pri-mirna':ti,ab,kw OR 'pri mirna':ti,ab,kw OR 'strna':ti,ab,kw OR 'small temporal rna':ti,ab,kw OR 'pre-mirna':ti,ab,kw OR 'pre mirna':ti,ab,kw OR 'micrornas':ti,ab,kw OR 'kass':ti,ab,kw | 245863 |
| #3 | 'microrna'/exp | 261234 |
| #2 | 'leukocyte antigen'/exp | 1178732 |
| #1 | 'keloid'/exp | 9140 |

**Search strategy of Cochrane Library**

| NO. | Search deatiles | Hits |
| --- | --- | --- |
| #1 | MeSH descriptor: [Keloid] explode all trees | 235 |
| #2 | MeSH descriptor: [Antigens, CD] explode all trees | 1265 |
| #3 | MeSH descriptor: [MicroRNAs] explode all trees | 563 |
| #4 | (CD Antigens):ti,ab,kw OR (CD Antigen):ti,ab,kw OR (Cluster of Differentiation Antigens):ti,ab,kw OR (Cluster of Differentiation Marker):ti,ab,kw OR (Differentiation Marker Cluster):ti,ab,kw OR (Cluster of Differentiation Markers):ti,ab,kw OR (Cluster of Differentiation Antigen):ti,ab,kw OR (Differentiation Antigen Cluster):ti,ab,kw OR (Leukocyte Differentiation Antigens, Human):ti,ab,kw OR (leucocyte antigen):ti,ab,kw OR (leukocyte antigen):ti,ab,kw OR (MicroRNA):ti,ab,kw OR (miRNAs):ti,ab,kw OR (Micro RNA):ti,ab,kw OR (miRNA):ti,ab,kw OR (Primary MicroRNA):ti,ab,kw OR (Primary miRNA):ti,ab,kw OR (pri-miRNA):ti,ab,kw OR (pri miRNA):ti,ab,kw OR (stRNA):ti,ab,kw OR (Small Temporal RNA):ti,ab,kw OR (pre-miRNA):ti,ab,kw OR (pre miRNA):ti,ab,kw OR (microRNAs):ti,ab,kw OR (KASS):ti,ab,kw | 4684 |
| #5 | MeSH descriptor: [Occlusive Dressings] explode all trees | 530 |
| #6 | MeSH descriptor: [Imiquimod] explode all trees | 327 |
| #7 | MeSH descriptor: [Steroids] explode all trees | 74627 |
| #8 | MeSH descriptor: [Mitomycins] in all MeSH products | 1568 |
| #9 | MeSH descriptor: [Fluorouracil] explode all trees | 8159 |
| #10 | MeSH descriptor: [Interferons] explode all trees | 7063 |
| #11 | MeSH descriptor: [Bleomycin] explode all trees | 1033 |
| #12 | MeSH descriptor: [Surgical Procedures, Operative] explode all trees | 172165 |
| #13 | MeSH descriptor: [Cryotherapy] explode all trees | 2265 |
| #14 | MeSH descriptor: [Radiotherapy] explode all trees | 9855 |
| #15 | MeSH descriptor: [Lasers, Dye] explode all trees | 130 |
| #16 | MeSH descriptor: [Platelet-Rich Plasma] explode all trees | 1237 |
| #17 | (Occlusive Dressing):ti,ab,kw OR (Occlusive Bandage):ti,ab,kw OR (Occlusive Bandages):ti,ab,kw OR (Spray-On Dressing):ti,ab,kw OR (occlusive dressings):ti,ab,kw OR (Compressive therapy):ti,ab,kw OR (Intralesional steroids):ti,ab,kw OR (Steroid):ti,ab,kw OR (Catatoxic Steroids):ti,ab,kw OR (cyclosteroids):ti,ab,kw OR (steroid compound):ti,ab,kw OR (steroid derivative):ti,ab,kw OR (steroidal compound):ti,ab,kw OR (steroids):ti,ab,kw OR (S 26308):ti,ab,kw OR (R 837):ti,ab,kw OR (Zyclara):ti,ab,kw OR (Aldara):ti,ab,kw OR (Mitomycin C):ti,ab,kw OR (Mitocin C):ti,ab,kw OR (NSC 26980):ti,ab,kw OR (Ametycine):ti,ab,kw OR (Mutamycin):ti,ab,kw OR (ameticine):ti,ab,kw OR (ametycin):ti,ab,kw OR (datisan):ti,ab,kw OR (jelmyto):ti,ab,kw OR (metomit):ti,ab,kw OR (mitocyn c):ti,ab,kw OR (mitocyna):ti,ab,kw OR (mitomicina-c):ti,ab,kw OR (mitomycin-c kyowa):ti,ab,kw OR (mitomycine):ti,ab,kw OR (mitomycine c):ti,ab,kw OR (mitosol):ti,ab,kw OR (mitozytrex):ti,ab,kw OR (mixandex):ti,ab,kw OR (mmc):ti,ab,kw OR (mytocine):ti,ab,kw OR (mytomicin c):ti,ab,kw OR (mytomycin c):ti,ab,kw OR (mytozytrex):ti,ab,kw OR (vetio):ti,ab,kw OR (mitomycin):ti,ab,kw OR (5 Fluorouracil):ti,ab,kw OR (Fluoruracil):ti,ab,kw OR (5 FU Lederle):ti,ab,kw OR (5 FU Medac):ti,ab,kw OR (5 HU Hexal):ti,ab,kw OR (Adrucil):ti,ab,kw OR (Carac):ti,ab,kw OR (Efudix):ti,ab,kw OR (Fluoro Uracile ICN):ti,ab,kw OR (Efudex):ti,ab,kw OR (Fluoroplex):ti,ab,kw OR (Flurodex):ti,ab,kw OR (Fluorouracil Mononitrate):ti,ab,kw OR (Fluorouracil Monopotassium Salt):ti,ab,kw OR (Fluorouracil Monosodium Salt):ti,ab,kw OR (Fluorouracil Potassium Salt):ti,ab,kw OR (Fluorouracil GRY):ti,ab,kw OR (Fluorouracile Dakota):ti,ab,kw OR (Fluorouracilo Ferrer Far):ti,ab,kw OR (Fluracedyl):ti,ab,kw OR (Haemato FU):ti,ab,kw OR (Neofluor):ti,ab,kw OR (Onkofluor):ti,ab,kw OR (Ribofluor):ti,ab,kw OR (5 Fluorouracil Biosyn):ti,ab,kw OR (5 fluoruracil):ti,ab,kw OR (accusite):ti,ab,kw OR (actino-hermal):ti,ab,kw OR (agicil):ti,ab,kw OR (cinkef u):ti,ab,kw OR (effluderm):ti,ab,kw OR (efurix):ti,ab,kw OR (eurofluor):ti,ab,kw OR (fivoflu):ti,ab,kw OR (fluoro uracil):ti,ab,kw OR (fluoroblastin):ti,ab,kw OR (fluorouracil 5):ti,ab,kw OR (fluorouracil sodium):ti,ab,kw OR (fluorouracile):ti,ab,kw OR (fluorouracilo):ti,ab,kw OR (fluouracil):ti,ab,kw OR (fluoxan):ti,ab,kw OR (flurablastin):ti,ab,kw OR (fluracil):ti,ab,kw OR (fluracilium):ti,ab,kw OR (fluril):ti,ab,kw OR (fluro uracil):ti,ab,kw OR (fluroblastin):ti,ab,kw OR (fluroblastine):ti,ab,kw OR (ifacil):ti,ab,kw OR (nsc 18913):ti,ab,kw OR (nsc 19893):ti,ab,kw OR (oncofu):ti,ab,kw OR (tolak):ti,ab,kw OR (uflahex):ti,ab,kw OR (uraciflor):ti,ab,kw OR (utoral):ti,ab,kw OR (fluorouracil):ti,ab,kw OR (Interferon):ti,ab,kw OR (cl 884):ti,ab,kw OR (endogenous interferon):ti,ab,kw OR (exogenic interferon):ti,ab,kw OR (ifn):ti,ab,kw OR (interferon type i):ti,ab,kw OR (interferone):ti,ab,kw OR (interferonogen):ti,ab,kw OR (interferons):ti,ab,kw OR (interferron):ti,ab,kw OR (Bleomycins):ti,ab,kw OR (BLEO cell):ti,ab,kw OR (Bleolem):ti,ab,kw OR (Bléomycine Bellon):ti,ab,kw OR (Bleomycin Sulfate):ti,ab,kw OR (Bleomycinum Mack):ti,ab,kw OR (Blenoxane):ti,ab,kw OR (Blanoxan):ti,ab,kw OR (Bleomicina):ti,ab,kw OR (bileco):ti,ab,kw OR (bl 19125):ti,ab,kw OR (blenamax):ti,ab,kw OR (bleo):ti,ab,kw OR (bleocin):ti,ab,kw OR (bleocina):ti,ab,kw OR (bleocris):ti,ab,kw OR (bleomycin analog):ti,ab,kw OR (bleomycin derivative):ti,ab,kw OR (bleomycin sulphate):ti,ab,kw OR (bleomycine):ti,ab,kw OR (bleomycinum):ti,ab,kw OR (blexit):ti,ab,kw OR (blocamicina):ti,ab,kw OR (nsc 125066):ti,ab,kw OR (bleomycin):ti,ab,kw OR (Operative Procedures):ti,ab,kw OR (Operative Procedure):ti,ab,kw OR (Operative Surgical Procedures):ti,ab,kw OR (Surgical Procedures):ti,ab,kw OR (Surgical Procedure):ti,ab,kw OR (Operative Surgical Procedure):ti,ab,kw OR (Ghost Surgery):ti,ab,kw OR (operation):ti,ab,kw OR (operation care):ti,ab,kw OR (operative intervention):ti,ab,kw OR (operative repair):ti,ab,kw OR (operative restoration):ti,ab,kw OR (operative surgery):ti,ab,kw OR (operative treatment):ti,ab,kw OR (research surgery):ti,ab,kw OR (resection):ti,ab,kw OR (surgical care):ti,ab,kw OR (surgical correction):ti,ab,kw OR (surgical diagnosis):ti,ab,kw OR (surgical diagnostic techniques):ti,ab,kw OR (surgical exposure):ti,ab,kw OR (surgical intervention):ti,ab,kw OR (surgical management):ti,ab,kw OR (surgical operation):ti,ab,kw OR (surgical practice):ti,ab,kw OR (surgical repair):ti,ab,kw OR (surgical research):ti,ab,kw OR (surgical restoration):ti,ab,kw OR (surgical service):ti,ab,kw OR (surgical speciality):ti,ab,kw OR (surgical specialties):ti,ab,kw OR (surgical specialty):ti,ab,kw OR (surgical therapy):ti,ab,kw OR (surgical treatment):ti,ab,kw OR (surgery):ti,ab,kw OR (Cryotherapies):ti,ab,kw OR (Cold Therapy):ti,ab,kw OR (Cold Therapies):ti,ab,kw OR (bath, cold):ti,ab,kw OR (cold bath):ti,ab,kw OR (cryogenic therapy):ti,ab,kw OR (cryothermy):ti,ab,kw OR (cryotreatment):ti,ab,kw OR (cryotherapy):ti,ab,kw OR (Radiotherapies):ti,ab,kw OR (Radiation Therapy):ti,ab,kw OR (Radiation Therapies):ti,ab,kw OR (Radiation Treatment):ti,ab,kw OR (Radiation Treatments):ti,ab,kw OR (Targeted Radiotherapies):ti,ab,kw OR (Targeted Radiotherapy):ti,ab,kw OR (Targeted Radiation Therapy):ti,ab,kw OR (Targeted Radiation Therapies):ti,ab,kw OR (bioradiant therapy):ti,ab,kw OR (bucky irradiation):ti,ab,kw OR (bucky radiation):ti,ab,kw OR (bucky radiotherapy):ti,ab,kw OR (bucky ray):ti,ab,kw OR (bucky ray radiation):ti,ab,kw OR (bucky therapy):ti,ab,kw OR (fractionated radiotherapy):ti,ab,kw OR (hemibody irradiation):ti,ab,kw OR (hypophysis irradiation):ti,ab,kw OR (hypophysis radiation):ti,ab,kw OR (irradiation therapy):ti,ab,kw OR (irradiation treatment):ti,ab,kw OR (lymphatic irradiation):ti,ab,kw OR (pituitary irradiation):ti,ab,kw OR (radiation beam centration):ti,ab,kw OR (radiation repair):ti,ab,kw OR (radio therapy):ti,ab,kw OR (radio treatment):ti,ab,kw OR (radiohypophysectomy):ti,ab,kw OR (radiotreatment):ti,ab,kw OR (roentgen therapy):ti,ab,kw OR (roentgen treatment):ti,ab,kw OR (rontgen therapy):ti,ab,kw OR (therapeutic radiology):ti,ab,kw OR (x radiotherapy):ti,ab,kw OR (x ray therapy):ti,ab,kw OR (x ray treatment):ti,ab,kw OR (radiotherapy):ti,ab,kw OR (Dye Laser):ti,ab,kw OR (Tunable Dye Lasers):ti,ab,kw OR (Tunable Dye Laser):ti,ab,kw OR (Dye Lasers):ti,ab,kw OR (Pulsed Dye Lasers):ti,ab,kw OR (Pulsed Dye Laser):ti,ab,kw OR (dye laser device):ti,ab,kw OR (tunable dye laser device):ti,ab,kw OR (Ablative laser):ti,ab,kw OR (Laser-assisted drug delivery):ti,ab,kw OR (LADD):ti,ab,kw OR (Platelet Rich Plasma):ti,ab,kw OR (platelet-rich plasma):ti,ab,kw OR (thrombocyte rich plasma):ti,ab,kw OR (stem cell therapy): ti,ab,kw OR (gene therapy):ti,ab,kw | 469503 |
| #18 | #1 AND (#2 OR #3 OR #4 OR #5 OR #6 OR #7 OR #8 OR #9 OR #10 OR #11 OR #12 OR #13 OR #14 OR #15 OR #16 OR #17) | 195 |

**Search strategy of Web of science**

| NO. | Search deatiles | Hits |
| --- | --- | --- |
| #1 | TS=(Keloids) | 9249 |
| #2 | (((((((((((((((((((((((TS=(CD Antigens) OR TS=(CD Antigen)) OR TS=(Cluster of Differentiation Antigens)) OR TS=(Cluster of Differentiation Marker)) OR TS=(Differentiation Marker Cluster)) OR TS=(Cluster of Differentiation Markers)) OR TS=(Cluster of Differentiation Antigen)) OR TS=(Differentiation Antigen Cluster)) OR TS=(Leukocyte Differentiation Antigens, Human)) OR TS=(leucocyte antigen)) OR TS=(leukocyte antigen)) OR TS=(MicroRNA)) OR TS=(miRNAs)) OR TS=(Micro RNA)) OR TS=(miRNA)) OR TS=(Primary MicroRNA)) OR TS=(Primary miRNA)) OR TS=(pri-miRNA)) OR TS=(pri miRNA)) OR TS=(stRNA)) OR TS=(Small Temporal RNA)) OR TS=(pre-miRNA)) OR TS=(pre miRNA)) OR TS=(microRNAs)) OR TS=(KASS) | 513298 |
| #3 | (((((((((((((((((((((((((((((((((((((((((((((((((((((((((((((((((((((((((((((((((((((((((((((((((((((((((((((((((((((((((((((((((((((((((((((((((((((((((((((((((((((((((((((((((((((((((((((((((((((((((((((((((((((((((((((((((((((((((((TS=(Occlusive Dressing) OR TS=(Occlusive Bandage)) OR TS=(Occlusive Bandages)) OR TS=(Spray-On Dressing)) OR TS=(Spray-On Dressings)) OR TS=(occlusive dressings)) OR TS=(Compressive therapy)) OR TS=(Intralesional steroids)) OR TS=(Steroid)) OR TS=(Catatoxic Steroids)) OR TS=(cyclosteroids)) OR TS=(steroid compound)) OR TS=(steroid derivative)) OR TS=(steroidal compound)) OR TS=(steroids)) OR TS=(S 26308)) OR TS=(R 837)) OR TS=(Zyclara)) OR TS=(Aldara)) OR TS=(Mitomycin C)) OR TS=(Mitocin C)) OR TS=(NSC 26980)) OR TS=(Ametycine)) OR TS=(Mutamycin)) OR TS=(ameticine)) OR TS=(ametycin)) OR TS=(datisan)) OR TS=(jelmyto)) OR TS=(metomit)) OR TS=(mitocyn c)) OR TS=(mitocyna)) OR TS=(mitomicina-c)) OR TS=(mitomycin-c kyowa)) OR TS=(mitomycine)) OR TS=(mitomycine c)) OR TS=(mitosol)) OR TS=(mitozytrex)) OR TS=(mixandex)) OR TS=(mmc)) OR TS=(mytocine)) OR TS=(mytomicin c)) OR TS=(mytomycin c)) OR TS=(mytozytrex)) OR TS=(vetio)) OR TS=(mitomycin)) OR TS=(5-FU)) OR TS=(5 Fluorouracil)) OR TS=(Fluoruracil)) OR TS=(5 FU Lederle)) OR TS=(5 FU Medac)) OR TS=(5 HU Hexal)) OR TS=(Adrucil)) OR TS=(Carac)) OR TS=(Efudix)) OR TS=(Fluoro Uracile ICN)) OR TS=(Efudex)) OR TS=(Fluoroplex)) OR TS=(Flurodex)) OR TS=(Fluorouracil Mononitrate)) OR TS=(Fluorouracil Monopotassium Salt)) OR TS=(Fluorouracil Monosodium Salt)) OR TS=(Fluorouracil Potassium Salt)) OR TS=(Fluorouracil GRY)) OR TS=(Fluorouracile Dakota)) OR TS=(Fluorouracilo Ferrer Far)) OR TS=(Fluracedyl)) OR TS=(Haemato FU)) OR TS=(Neofluor)) OR TS=(Onkofluor)) OR TS=(Ribofluor)) OR TS=(5 Fluorouracil Biosyn)) OR TS=(5 fluoruracil)) OR TS=(accusite)) OR TS=(actino-hermal)) OR TS=(agicil)) OR TS=(cinkef u)) OR TS=(effluderm)) OR TS=(efurix)) OR TS=(eurofluor)) OR TS=(fivoflu)) OR TS=(fluoro uracil)) OR TS=(fluoroblastin)) OR TS=(fluorouracil 5)) OR TS=(fluorouracil sodium)) OR TS=(fluorouracile)) OR TS=(fluorouracilo)) OR TS=(fluouracil)) OR TS=(fluoxan)) OR TS=(flurablastin)) OR TS=(fluracil)) OR TS=(fluracilium)) OR TS=(fluril)) OR TS=(fluro uracil)) OR TS=(fluroblastin)) OR TS=(fluroblastine)) OR TS=(ifacil)) OR TS=(nsc 18913)) OR TS=(nsc 19893)) OR TS=(oncofu)) OR TS=(tolak)) OR TS=(uflahex)) OR TS=(uraciflor)) OR TS=(utoral)) OR TS=(fluorouracil)) OR TS=(Interferon)) OR TS=(cl 884)) OR TS=(endogenous interferon)) OR TS=(exogenic interferon)) OR TS=(ifn)) OR TS=(interferon type i)) OR TS=(interferone)) OR TS=(interferonogen)) OR TS=(interferons)) OR TS=(interferron)) OR TS=(Bleomycins)) OR TS=(BLEO cell)) OR TS=(Bleolem)) OR TS=(Bléomycine Bellon)) OR TS=(Bleomycin Sulfate)) OR TS=(Bleomycinum Mack)) OR TS=(Blenoxane)) OR TS=(Blanoxan)) OR TS=(Bleomicina)) OR TS=(bileco)) OR TS=(bl 19125)) OR TS=(blenamax)) OR TS=(bleo)) OR TS=(bleocin)) OR TS=(bleocina)) OR TS=(bleocris)) OR TS=(bleomycin analog)) OR TS=(bleomycin derivative)) OR TS=(bleomycin sulphate)) OR TS=(bleomycine)) OR TS=(bleomycinum)) OR TS=(blexit)) OR TS=(blocamicina)) OR TS=(nsc 125066)) OR TS=(bleomycin)) OR TS=(Operative Procedures)) OR TS=(Operative Procedure)) OR TS=(Operative Surgical Procedures)) OR TS=(Surgical Procedures)) OR TS=(Surgical Procedure)) OR TS=(Operative Surgical Procedure)) OR TS=(Ghost Surgery)) OR TS=(operation)) OR TS=(operation care)) OR TS=(operative intervention)) OR TS=(operative repair)) OR TS=(operative restoration)) OR TS=(operative surgery)) OR TS=(operative treatment)) OR TS=(research surgery)) OR TS=(resection)) OR TS=(surgical care)) OR TS=(surgical correction)) OR TS=(surgical diagnosis)) OR TS=(surgical diagnostic techniques)) OR TS=(surgical exposure)) OR TS=(surgical intervention)) OR TS=(surgical management)) OR TS=(surgical operation)) OR TS=(surgical practice)) OR TS=(surgical repair)) OR TS=(surgical research)) OR TS=(surgical restoration)) OR TS=(surgical service)) OR TS=(surgical speciality)) OR TS=(surgical specialties)) OR TS=(surgical specialty)) OR TS=(surgical therapy)) OR TS=(surgical treatment)) OR TS=(surgery)) OR TS=(Cryotherapies)) OR TS=(Cold Therapy)) OR TS=(Cold Therapies)) OR TS=(bath, cold)) OR TS=(cold bath)) OR TS=(cryogenic therapy)) OR TS=(cryothermy)) OR TS=(cryotreatment)) OR TS=(cryotherapy)) OR TS=(Radiotherapies)) OR TS=(Radiation Therapy)) OR TS=(Radiation Therapies)) OR TS=(Radiation Treatment)) OR TS=(Radiation Treatments)) OR TS=(Targeted Radiotherapies)) OR TS=(Targeted Radiotherapy)) OR TS=(Targeted Radiation Therapy)) OR TS=(Targeted Radiation Therapies)) OR TS=(bioradiant therapy)) OR TS=(bucky irradiation)) OR TS=(bucky radiation)) OR TS=(bucky radiotherapy)) OR TS=(bucky ray)) OR TS=(bucky ray radiation)) OR TS=(bucky therapy)) OR TS=(fractionated radiotherapy)) OR TS=(hemibody irradiation)) OR TS=(hypophysis irradiation)) OR TS=(hypophysis radiation)) OR TS=(irradiation therapy)) OR TS=(irradiation treatment)) OR TS=(lymphatic irradiation)) OR TS=(pituitary irradiation)) OR TS=(radiation beam centration)) OR TS=(radiation repair)) OR TS=(radio therapy)) OR TS=(radio treatment)) OR TS=(radiohypophysectomy)) OR TS=(radiotreatment)) OR TS=(roentgen therapy)) OR TS=(roentgen treatment)) OR TS=(rontgen therapy)) OR TS=(therapeutic radiology)) OR TS=(x radiotherapy)) OR TS=(x ray therapy)) OR TS=(x ray treatment)) OR TS=(radiotherapy)) OR TS=(Dye Laser)) OR TS=(Tunable Dye Lasers)) OR TS=(Tunable Dye Laser)) OR TS=(Dye Lasers)) OR TS=(Pulsed Dye Lasers)) OR TS=(Pulsed Dye Laser)) OR TS=(dye laser device)) OR TS=(tunable dye laser device)) OR TS=(Ablative laser)) OR TS=(Laser-assisted drug delivery)) OR TS=(LADD)) OR TS=(Platelet Rich Plasma)) OR TS=(platelet-rich plasma)) OR TS=(thrombocyte rich plasma)) OR TS=( stem cell therapy)) OR TS= (gene therapy) | 10414776 |
| #4 | #1 AND (#2 OR #3) | 4572 |
| #5 | #1 AND (#2 OR #3) and Article (Document Types) | 4093 |
