# Supplementary material for: Therapeutic methods and effect on keloid and hypertrophic scars: a systematic review
Source: Front Med (Lausanne). 2026 Mar 11;13:1702697. doi: 10.3389/fmed.2026.1702697 (PMC13013025; doi:10.3389/fmed.2026.1702697)
Supplement: Supplementary file 2 [file Table_2.docx]

Table S2 Quality assessment results of the included RCTs

| **Study** | **D1** | **D2** | **D3** | **D4** | **D5** | **Overall** |
| --- | --- | --- | --- | --- | --- | --- |
| Daoud 2019 | Low | Low | Low | Low | Low | Low |
| MANUSKIATTI 2021 | Low | Low | Low | Low | Low | Low |
| Ahuja 2013 | Low | Low | Some concerns | Low | Low | Some concerns |
| Al-Mohamady 2016 | Low | Low | Low | Low | Some concerns | Some concerns |
| Tawfic 2020 | Low | Low | Low | Low | Some concerns | Some concerns |
| Hatamipour 2009 | Low | Low | Low | Low | Low | Low |
| Saha 2012 | Low | Low | Low | Low | Low | Low |
| Khattab 2019 | Low | Low | Low | Low | Low | Low |
| Kaushal 2020 | Low | Low | Low | Low | Low | Low |
| Sabry 2020 | Low | Low | Low | Some concerns | Low | Some concerns |
| Abedini 2018 | Low | Low | Low | Low | Low | Low |
| ASILIAN 2006 | Low | Low | Low | Low | Low | Low |
| Behera 2016 | Low | Low | Low | Low | Low | Low |
| Bijlard 2018 | Low | Low | Low | Low | Low | Low |
| Monteiro 2022 | Low | Low | Low | Low | Low | Low |
| Li 2022 | Low | Low | Some concerns | Low | Low | Some concerns |
| Darougheh 2007 | Low | Low | Some concerns | Low | Low | Some concerns |
| Davari 2012 | Low | Low | Low | Low | Low | Low |
| Moravej 2022 | Low | Low | Some concerns | Low | Low | Some concerns |
| Saki 2019 | Low | Low | Some concerns | Low | Low | Some concerns |
| Chen 2017 | Low | Low | Some concerns | Low | Low | Some concerns |
| Friedman 2020 | Low | Low | Low | Low | Low | Low |
| SONG 2018 | Low | Low | Low | Some concerns | Low | Some concerns |
| Burusapat 2021 | Low | Low | Low | Some concerns | Low | Some concerns |
| Hewedy 2020 | Low | Low | Low | Low | Low | Low |
| Hietanen 2018 | Low | Low | Low | Low | Low | Low |
| Tawaranurak 2022 | Low | Low | Some concerns | Low | Low | Some concerns |
| Shaarawy 2014 | Low | Low | Low | Low | Low | Low |
| Ramadan 2021 | Low | Low | Low | Low | Low | Low |
| Suwanchinda 2022 | Low | Low | Some concerns | Low | Low | Some concerns |
| Neinaa 2021 | Low | Low | Low | Low | Low | Low |
| Nor 2016 | Low | Low | Low | Low | Low | Low |
| Noruri 2003 | Low | Low | Low | Low | Low | Low |
| Khalid 2018 | Low | Low | Low | Low | Low | Low |
| Khan 2019 | Low | Low | Low | Low | Low | Low |
| Khedr 2019 | Low | Low | Low | Low | Low | Low |
| Manuskiatti 2021 | Low | Low | Some concerns | Low | Low | Some concerns |
| Erlendsson 2022 | Low | Low | Low | Low | Low | Low |
| Deng 2021 | Low | Low | Low | Low | Low | Low |
| Rasaii 2018 | Low | Low | Low | Low | Low | Low |
| Disphanurat 2023 | Low | Low | Low | Low | Low | Low |
| Wittenberg 1999 | Low | Low | Low | Some concerns | Low | Some concerns |
| Srivastava 2018 | Low | Low | Low | Low | Low | Low |
| Sunil 2018 | Low | Low | Low | Low | Low | Low |
| Zouboulis 2020 | Low | Low | Some concerns | Low | Low | Some concerns |
| Sabry 2019 | Low | Low | Low | Low | Low | Low |
| SADEGHINIA 2012 | Low | Low | Low | Low | Low | Low |
| Hou 2023 | Low | Low | Low | Low | Low | Low |
| D1: Bias arising from the randomization process;  D2: Bias due to deviations from intended intervention;  D3: Bias due to missing outcome data;  D4: Bias in measurement of the outcome;  D5: Bias in selection of the reported redult; | | | | | | |
