# Supplementary material for: Therapeutic methods and effect on keloid and hypertrophic scars: a systematic review
Source: Front Med (Lausanne). 2026 Mar 11;13:1702697. doi: 10.3389/fmed.2026.1702697 (PMC13013025; doi:10.3389/fmed.2026.1702697)
Supplement: Supplementary file 3 [file Table_3.docx]

Table S3 Quality assessment results of the included non-RCTs

| **Study** | **T1** | **T2** | **T3** | **T4** | **T5** | **T6** | **T7** | **T8** | **T9** | **T10** | **T11** | **T12** | **Total** |
| --- | --- | --- | --- | --- | --- | --- | --- | --- | --- | --- | --- | --- | --- |
| Son 2014 | 2 | 2 | 2 | 1 | 2 | 2 | 2 | 2 |  |  |  |  | 15 |
| Lee 2015 | 2 | 2 | 2 | 2 | 2 | 2 | 2 | 2 |  |  |  |  | 16 |
| MARTIN-GARCIA 2005 | 2 | 2 | 2 | 2 | 2 | 2 | 2 | 2 |  |  |  |  | 16 |
| Kant 2018 | 2 | 2 | 2 | 2 | 2 | 0 | 2 | 2 |  |  |  |  | 14 |
| Li 2020 | 2 | 2 | 2 | 2 | 2 | 0 | 2 | 2 |  |  |  |  | 14 |
| Wen 2021 | 2 | 2 | 2 | 2 | 2 | 2 | 2 | 2 |  |  |  |  | 16 |
| Daurade 2020 | 2 | 2 | 2 | 1 | 2 | 2 | 2 | 2 |  |  |  |  | 15 |
| Chen 2020 | 2 | 2 | 2 | 2 | 2 | 2 | 2 | 2 |  |  |  |  | 16 |
| Wang 2020 | 2 | 2 | 2 | 2 | 2 | 2 | 2 | 2 |  |  |  |  | 16 |
| Levenberg 2020 | 2 | 2 | 2 | 2 | 2 | 2 | 2 | 2 |  |  |  |  | 16 |
| Bonnardeaux 2019 | 2 | 2 | 2 | 2 | 2 | 2 | 2 | 2 |  |  |  |  | 16 |
| Seo 2011 | 2 | 2 | 2 | 2 | 2 | 2 | 2 | 2 |  |  |  |  | 16 |
| Arnault 2009 | 2 | 2 | 2 | 2 | 2 | 0 | 2 | 2 |  |  |  |  | 14 |
| Yii 1996 | 2 | 2 | 2 | 2 | 2 | 0 | 2 | 2 |  |  |  |  | 14 |
| Bijlard 2017 | 2 | 2 | 2 | 2 | 2 | 0 | 2 | 2 |  |  |  |  | 14 |
| Bischof 2007 | 2 | 2 | 2 | 2 | 2 | 2 | 2 | 2 |  |  |  |  | 16 |
| Francisco 2013 | 2 | 2 | 2 | 2 | 2 | 0 | 2 | 2 |  |  |  |  | 14 |
| Cho 2010 | 2 | 2 | 2 | 2 | 2 | 2 | 2 | 2 |  |  |  |  | 16 |
| Chopinaud 2014 | 2 | 2 | 2 | 2 | 2 | 0 | 2 | 2 |  |  |  |  | 14 |
| Clavere 1997 | 2 | 2 | 2 | 2 | 2 | 0 | 2 | 2 |  |  |  |  | 14 |
| Copcu 2004 | 2 | 2 | 2 | 2 | 2 | 2 | 2 | 2 |  |  |  |  | 16 |
| CARETA 2013 | 2 | 2 | 2 | 2 | 2 | 0 | 2 | 2 |  |  |  |  | 14 |
| CARVALHAES 2015 | 2 | 2 | 2 | 2 | 2 | 2 | 2 | 2 |  |  |  |  | 16 |
| Espana 2001 | 2 | 2 | 2 | 2 | 2 | 0 | 2 | 2 |  |  |  |  | 14 |
| Weshahy 2012 | 2 | 2 | 2 | 1 | 2 | 2 | 2 | 2 |  |  |  |  | 15 |
| Manjunath 2021 | 2 | 2 | 2 | 2 | 2 | 2 | 2 | 2 |  |  |  |  | 16 |
| Alhamzawi 20221 | 2 | 2 | 2 | 2 | 2 | 0 | 2 | 2 |  |  |  |  | 14 |
| Erol 2008 | 2 | 2 | 2 | 2 | 2 | 0 | 2 | 2 |  |  |  |  | 14 |
| Escarmant 1993 | 2 | 2 | 2 | 2 | 2 | 2 | 2 | 2 |  |  |  |  | 16 |
| Annabathula 2017 | 2 | 2 | 2 | 2 | 2 | 0 | 2 | 2 |  |  |  |  | 14 |
| BERMAN 2020 | 2 | 2 | 2 | 2 | 2 | 2 | 2 | 2 |  |  |  |  | 16 |
| Barragan 2022 | 2 | 2 | 2 | 2 | 2 | 2 | 2 | 2 |  |  |  |  | 16 |
| Jiang 2015 | 2 | 2 | 2 | 2 | 2 | 0 | 2 | 2 |  |  |  |  | 14 |
| Jiang 2017 | 2 | 2 | 2 | 2 | 2 | 2 | 2 | 2 |  |  |  |  | 16 |
| Guix 2001 | 2 | 2 | 2 | 2 | 2 | 2 | 2 | 2 |  |  |  |  | 16 |
| Hafkamp 2017 | 2 | 2 | 2 | 2 | 2 | 2 | 2 | 2 |  |  |  |  | 16 |
| Meymandi 2014 | 2 | 2 | 2 | 2 | 2 | 0 | 2 | 2 |  |  |  |  | 14 |
| Stephanides 2011 | 2 | 2 | 2 | 2 | 2 | 0 | 2 | 2 |  |  |  |  | 14 |
| Choi 2020 | 2 | 2 | 2 | 2 | 2 | 2 | 2 | 2 |  |  |  |  | 16 |
| Muneuchi 2009 | 2 | 2 | 2 | 2 | 2 | 2 | 2 | 2 |  |  |  |  | 16 |
| Kim 2015 | 2 | 2 | 2 | 2 | 2 | 0 | 2 | 2 |  |  |  |  | 14 |
| Kim 2022 | 2 | 2 | 2 | 1 | 2 | 2 | 2 | 2 |  |  |  |  | 15 |
| George 2005 | 2 | 2 | 2 | 2 | 2 | 2 | 2 | 2 |  |  |  |  | 16 |
| Hye 2015 | 2 | 2 | 2 | 2 | 2 | 0 | 2 | 2 |  |  |  |  | 14 |
| Maemoto 2020 | 2 | 2 | 2 | 2 | 2 | 2 | 2 | 2 |  |  |  |  | 16 |
| Berman 2002 | 2 | 2 | 2 | 2 | 2 | 2 | 2 | 2 |  |  |  |  | 16 |
| Ogawa 2002 | 2 | 2 | 2 | 1 | 2 | 2 | 2 | 2 |  |  |  |  | 15 |
| Aljodah 2021 | 2 | 2 | 2 | 2 | 2 | 2 | 2 | 2 |  |  |  |  | 16 |
| Park 2012 | 2 | 2 | 2 | 2 | 2 | 2 | 2 | 2 |  |  |  |  | 16 |
| Acosta 2016 | 2 | 2 | 2 | 2 | 2 | 2 | 2 | 2 |  |  |  |  | 16 |
| Ogawa 2014 | 2 | 2 | 2 | 2 | 2 | 2 | 2 | 2 |  |  |  |  | 16 |
| Ollstein 1981 | 2 | 2 | 2 | 2 | 2 | 2 | 2 | 2 |  |  |  |  | 16 |
| Reinholz 2020 | 2 | 2 | 2 | 1 | 2 | 2 | 2 | 2 |  |  |  |  | 15 |
| Song 2014 | 2 | 2 | 2 | 2 | 2 | 2 | 2 | 2 |  |  |  |  | 16 |
| Shen 2015 | 2 | 2 | 2 | 2 | 2 | 2 | 2 | 2 |  |  |  |  | 16 |
| Ragoowansi 2002 | 2 | 2 | 2 | 2 | 2 | 2 | 2 | 2 |  |  |  |  | 16 |
| Weshay 2015 | 2 | 2 | 2 | 2 | 2 | 2 | 2 | 2 |  |  |  |  | 16 |
| Agbenorku 2000 | 2 | 2 | 2 | 2 | 2 | 2 | 2 | 2 |  |  |  |  | 16 |
| Ahmad 2017 | 2 | 2 | 2 | 2 | 2 | 2 | 2 | 2 |  |  |  |  | 16 |
| Saray 2005 | 2 | 2 | 2 | 2 | 2 | 2 | 2 | 2 |  |  |  |  | 16 |
| Sruthi 2017 | 2 | 2 | 2 | 2 | 2 | 2 | 2 | 2 |  |  |  |  | 16 |
| Son 2020 | 2 | 2 | 2 | 2 | 2 | 2 | 2 | 2 |  |  |  |  | 16 |
| Song 2018 | 2 | 2 | 2 | 2 | 2 | 0 | 2 | 2 |  |  |  |  | 14 |
| Luo 2023 | 2 | 2 | 2 | 2 | 2 | 2 | 2 | 2 |  |  |  |  | 16 |
| Stern 1989 | 2 | 2 | 2 | 1 | 2 | 0 | 2 | 2 |  |  |  |  | 13 |
| Stewart 2006 | 2 | 2 | 2 | 2 | 2 | 2 | 2 | 2 |  |  |  |  | 16 |
| Leeuwen 2014 | 2 | 2 | 2 | 2 | 2 | 2 | 2 | 2 |  |  |  |  | 16 |
| VIANI 2009 | 2 | 2 | 2 | 2 | 2 | 2 | 2 | 2 |  |  |  |  | 16 |
| Wang 2020 | 2 | 2 | 2 | 2 | 2 | 2 | 2 | 2 |  |  |  |  | 16 |
| Davison 2009 | 2 | 2 | 2 | 2 | 2 | 2 | 2 | 2 | 1 | 1 | 1 | 1 | 20 |
| Dai 2021 | 2 | 2 | 2 | 2 | 2 | 2 | 2 | 2 | 1 | 1 | 1 | 1 | 20 |
| ANG 2013 | 2 | 2 | 2 | 2 | 2 | 2 | 2 | 2 | 1 | 1 | 1 | 1 | 20 |
| Nishi 2022 | 2 | 2 | 2 | 2 | 2 | 0 | 2 | 2 | 1 | 1 | 1 | 1 | 18 |
| Sharma 2021 | 2 | 2 | 2 | 2 | 2 | 0 | 2 | 2 | 1 | 1 | 1 | 1 | 18 |
| Abdel-Meguid 2014 | 2 | 2 | 2 | 2 | 2 | 0 | 2 | 2 | 1 | 1 | 1 | 1 | 18 |
| Lv 2021 | 2 | 2 | 2 | 2 | 2 | 0 | 2 | 2 | 1 | 1 | 1 | 1 | 18 |
| Meymandi 2016 | 2 | 2 | 2 | 2 | 2 | 0 | 2 | 2 | 1 | 1 | 1 | 1 | 18 |
| Yosipovitch 2009 | 2 | 2 | 2 | 1 | 2 | 0 | 2 | 2 | 1 | 1 | 1 | 1 | 17 |
| Emad 2010 | 2 | 2 | 2 | 2 | 2 | 0 | 2 | 2 | 1 | 1 | 1 | 1 | 18 |
| Hoang 2016 | 2 | 2 | 2 | 2 | 2 | 2 | 2 | 2 | 1 | 1 | 1 | 1 | 20 |
| Berman 1997 | 2 | 2 | 2 | 2 | 2 | 0 | 2 | 2 | 1 | 1 | 1 | 1 | 18 |
| Albalat 2021 | 2 | 2 | 2 | 2 | 2 | 0 | 2 | 2 | 1 | 1 | 1 | 1 | 18 |
| Payapvipapong 2014 | 2 | 2 | 2 | 2 | 2 | 2 | 2 | 2 | 1 | 1 | 1 | 1 | 20 |
| Dina 2021 | 2 | 2 | 2 | 2 | 2 | 0 | 2 | 2 | 1 | 1 | 1 | 1 | 18 |
| Stromps 2013 | 2 | 2 | 2 | 1 | 2 | 2 | 2 | 2 | 1 | 1 | 1 | 1 | 19 |
| Alexander 2018 | 2 | 2 | 2 | 2 | 2 | 0 | 2 | 2 | 1 | 1 | 1 | 1 | 18 |
| Gamil 2019 | 2 | 2 | 2 | 1 | 2 | 2 | 2 | 2 | 1 | 1 | 1 | 1 | 19 |
| Dogahe 2023 | 2 | 2 | 2 | 2 | 2 | 2 | 2 | 2 | 1 | 1 | 1 | 1 | 20 |
| Shin 2019 | 2 | 2 | 2 | 2 | 2 | 0 | 2 | 2 | 1 | 1 | 1 | 1 | 18 |
| Sharma 2007 | 2 | 2 | 2 | 1 | 2 | 2 | 2 | 2 | 1 | 1 | 1 | 1 | 19 |
| Gamil 2018 | 2 | 2 | 2 | 2 | 2 | 0 | 2 | 2 | 1 | 1 | 1 | 1 | 18 |
| Cicco 2013 | 2 | 2 | 2 | 2 | 2 | 2 | 2 | 2 | 1 | 1 | 1 | 1 | 20 |
| Chernoff 2007 | 2 | 2 | 2 | 2 | 2 | 0 | 2 | 2 | 1 | 1 | 1 | 1 | 18 |
| Tsai 2019 | 2 | 2 | 2 | 2 | 2 | 2 | 2 | 2 | 1 | 1 | 1 | 1 | 20 |
| Tawfik 2019 | 2 | 2 | 2 | 1 | 2 | 0 | 2 | 2 | 1 | 1 | 1 | 1 | 17 |
| Liu 2023 | 2 | 2 | 2 | 2 | 2 | 0 | 2 | 2 | 1 | 1 | 1 | 1 | 18 |
| Lee 2008 | 2 | 2 | 2 | 2 | 2 | 0 | 2 | 2 | 1 | 1 | 1 | 1 | 18 |
| Zawahry 2015 | 2 | 2 | 2 | 2 | 2 | 2 | 2 | 2 | 1 | 1 | 1 | 1 | 20 |
| Meseci 2019 | 2 | 2 | 2 | 2 | 2 | 2 | 2 | 2 | 1 | 1 | 1 | 1 | 20 |
| Qiao 2017 | 2 | 2 | 2 | 2 | 2 | 2 | 2 | 2 | 1 | 1 | 1 | 1 | 20 |
| Francesca 2010 | 2 | 2 | 2 | 1 | 2 | 0 | 2 | 2 | 1 | 1 | 1 | 1 | 17 |
| T1: A stated aim of the study  T2: Inclusion of consecutive patients  T3: Prospective collection of data  T4: Endpoint appropriate to the study aim  T5: Unbiased evaluation of endpoints  T6: Follow-up period appropriate to the major endpoint  T7: Loss to follow up not exceeding 5%  T8: Prospective calculation of the sample size  T9: A control group having the gold standard intervention  T10: Contemporary groups  T11: Baseline equivalence of groups  T12: Statistical analyses adapted to the study design | | | | | | | | | | | | | |
